# Exploring the excited state mechanisms of a viscosity-sensitive zeromethine merocyanine dye emitter

**DOI:** 10.1039/d6sc05772a

**Published:** 2026-10-12

**Authors:** Julian Fichtner, Preetika Verma, Menyhárt Sárosi, Matthias Stolte, Yapamanu Adithya Lakshmanna, Frank Würthner

**Affiliations:** a Institut für Organische Chemie, Julius-Maximilians-Universität Würzburg Am Hubland 97074 Würzburg Germany frank.wuerthner@uni-wuerzburg.de; b School of Chemistry, Indian Institute of Science Education and Research Thiruvananthapuram Vithura Thiruvananthapuram 695551 India adithya@iisertvm.ac.in; c Center for Nanosystems Chemistry (CNC), Julius-Maximilians-Universität Würzburg Theodor-Boveri-Weg 97074 Würzburg Germany

## Abstract

Merocyanine (MC) dyes are among the most versatile and tunable chromophores owing to their structural flexibility. However, their excited state dynamics, which often lead to fluorescence quenching by ultrafast non-radiative decay pathways such as photoisomerization or relaxation through conical intersections, are poorly understood for most MCs. Herein, we elucidate the excited state mechanisms of a zeromethine MC dye (PyBA), which represents a special case of the donor–acceptor architecture featuring two directly connected heterocycles. The analysis was achieved by combining steady-state measurements under varying temperature, viscosity and polarity conditions, time-resolved ultrafast spectroscopy, and quantum-chemical calculations. Our studies show that PyBA upon photoexcitation develops a twisted intramolecular charge transfer (TICT) state in dynamic environments (*e.g.*, CH_2_Cl_2_), whereas this process is restricted in rigid environments (PMMA matrix), leading to a significant luminescence turn-on with the quantum yield (*Φ*_PL_) increasing from 0.7% to 55%. Moreover, the measurements performed in the PMMA matrix reveal room temperature phosphorescence (RTP) even under ambient conditions, *i.e.* even without external stimuli, which becomes more intense at cryogenic temperatures under an argon atmosphere, reaching a phosphorescence lifetime of 705 ms. Furthermore, this work introduces a simple and time-efficient strategy for screening small-molecule fluorescence probes.

## Abstract 2

A viscosity-sensitive zeromethine merocyanine dye displays multifaceted environment-dependent photoluminescence consisting of TICT emission, fluorescence and phosphorescence, depending on the accessibility to torsional motions.

## Graphical abstract

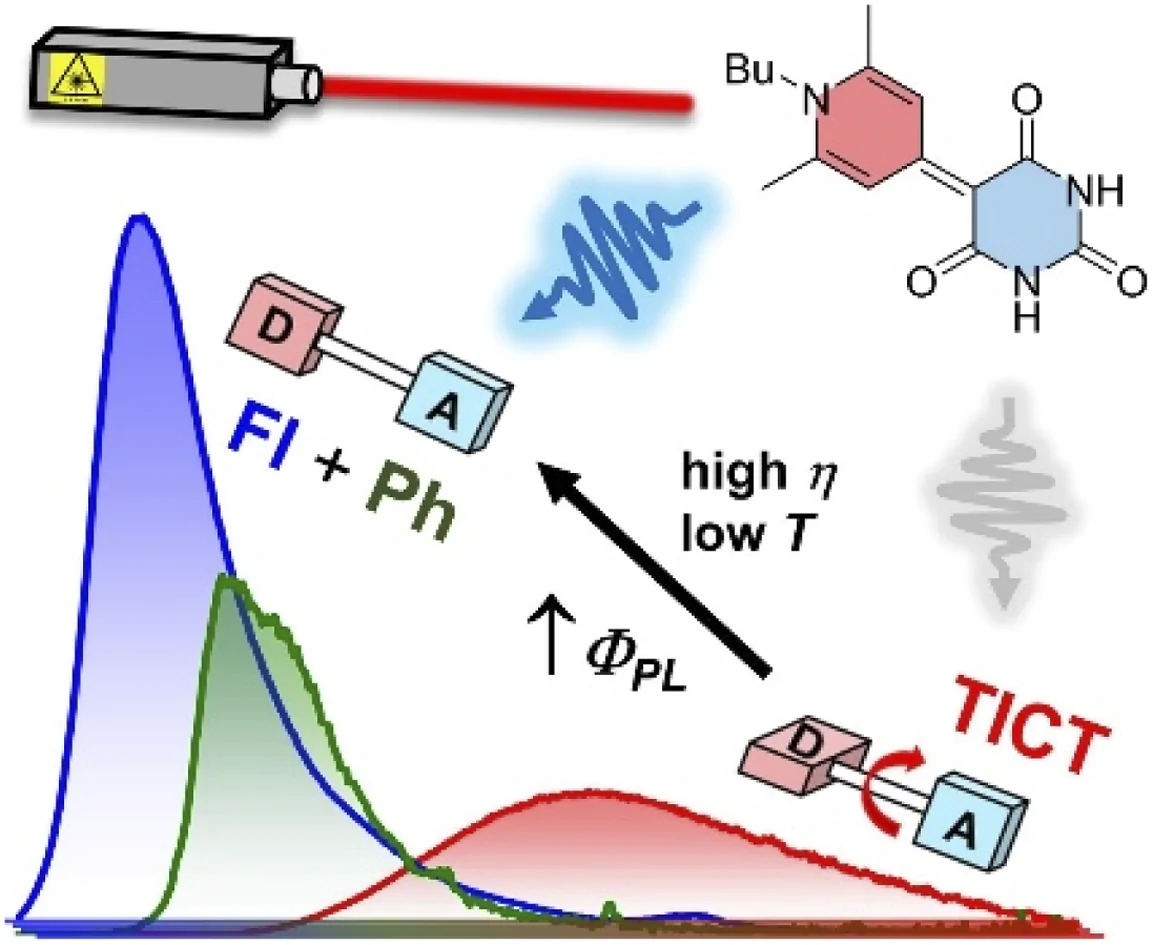

## Introduction

Compared to their relatives, donor–acceptor substituted polyenes/arylenes,^1^*e.g. p*-dimethylaminobenzonitrile DMABN,^2,3^ or (hemi-)cyanines, *e.g.* thiazole orange TO,^4,5^ merocyanine (MC) dyes have received less attention among the widely applied fluorophores.^6–8^ Indeed, the majority of MC dyes^9,10^ show only weak fluorescence and only a few reached the level required for wider applications.^11^ Representative examples are MC540 as a fluorescent membrane probe,^12,13^DCM as a laser dye^14^ and MCs with tricyanofuran acceptor groups such as TCF for single molecule spectroscopy (Fig. 1a).^15,16^ Considering the fact that no other class of dyes affords a wider tunability of optical properties (wavelength and bandwidth) than is possible for MC dyes by simple variation of donor and acceptor groups, length of the polymethine chain, as well as embedment of the latter within carbo- or heterocyclic aromatic or quinonoid units,^17–19^ it is indeed surprising that the excited state dynamics have been elucidated in detail for only a few prominent MC derivatives using ultrafast spectroscopy techniques combined with in-depth quantum chemical analyses. Among them, the DCM-related MC dye DCBT has been studied by some of us due to its unusual solvent dependence, showing weak fluorescence in non-polar media (*Φ*_PL_ = 1% in methylcyclohexane) and strong emission in polar media (*Φ*_PL_ = 67% in dimethylsulfoxide).^20^ Whilst this MC dye with its long polymethine chain was shown to exhibit several carbon–carbon bonds prone to rotational motion leading to conical intersections (CIs) upon photoexcitation, in the present study, we shed light on the excited state dynamics of another extreme case, the “zeromethine” 5-(1-butyl-2,6-dimethylpyridin-4(1*H*)-ylidene)-pyrimidine-2,4,6(1*H*,3*H*,5*H*)-trione PyBA, which consists of two directly connected heterocyclic subunits (Fig. 1b). Although the term zeromethine was established almost two decades ago,^21^ only a limited number of studies on this subclass of MCs have been reported so far,^22–24^ and even fewer provide a comprehensive analysis of the excited state properties. Moreover, PyBA is expected to be a promising candidate as a fluorescence molecular probe for biomacromolecules owing to its compact molecular architecture, its ability to engage in hydrogen bonding *via* the barbituric acid moiety, and its potential for (weak) π–π interactions through its heteroaromatic framework. These features provide a rational basis to further advance and expand upon our previous work, in which barbituric acid-containing MC dyes were incorporated into nucleic acid structures^25^ and larger self-assembled supramolecular systems.^26,27^ Motivated by these considerations, we sought to investigate this chromophore in greater detail, with particular emphasis on elucidating its competing non-radiative decay pathways.

**Fig. 1:**
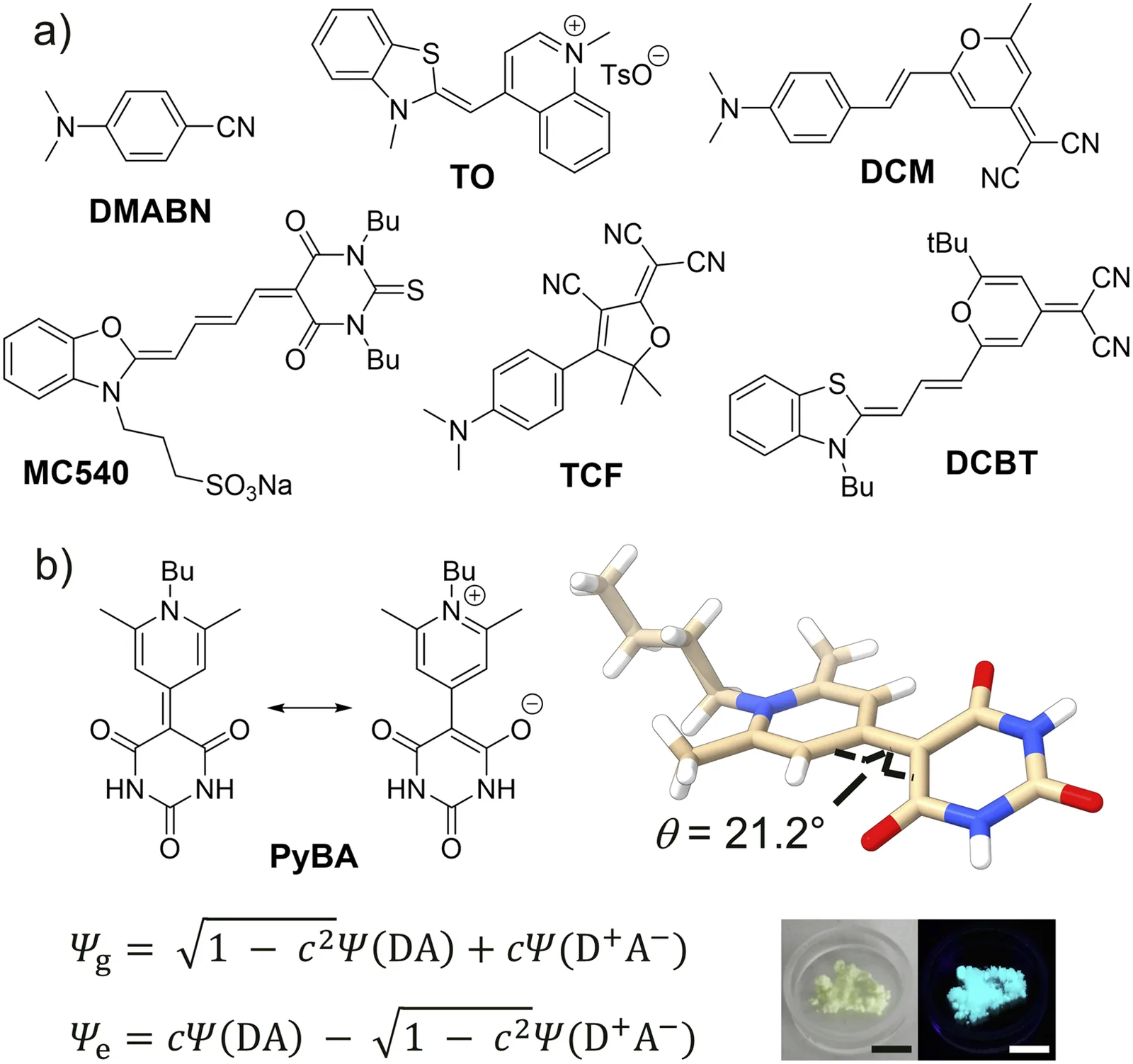
Molecular structures of (a) common polymethine fluorophores and (b) the herein investigated MC dye PyBA, as well as its crystal structure showing the pre-twisted geometry in the ground state with the torsional angle *θ*. Photographs of PyBA under ambient and UV light (*λ*_ex_ = 365 nm) are given, as well as the formulae to calculate the resonance parameter *c*^2^. The scale bar provided in the photographs equals 5 mm.

## Results

### Synthesis, structure and ground state properties

The dihydropyridine barbituric acid dye PyBA was readily obtained as a pale-yellow solid exhibiting blue emission under UV light from barbituric acid, 2,6-dimethyl-4*H*-pyran-4-one and *n*-butylamine in two synthetic steps (Scheme S1, SI). Single crystals grown from a methanol solution were subjected to X-ray crystallography (CCDC 2544677), revealing a pre-twisted structure in the ground state with a torsional angle of 21.2° around the central donor–acceptor double bond, indicative of partial single bond character (Fig. 1b). The crystal packing (Fig. S7, SI) further indicates that PyBA can engage in hydrogen bonding with its environment, as it is cross-linked to co-crystallized methanol *via* the barbituric acid moieties. Consequently, optical measurements were mainly restricted to aprotic solvents, in which hydrogen bonding and specific solvent effects are absent, since earlier studies demonstrated their strong influence on the photoluminescence of MC dyes constructed with carbonyl-containing acceptor units.^28^ Furthermore, an electro-optical absorption measurement (EOAM) in 1,4-dioxane suggests a charge-separated zwitterionic character of PyBA in its ground state with a *c*^2^ value of 0.64 (Fig. S8 and Table S5, SI), consistent with its partial single bond character and propensity for torsional motions. The UV-Vis absorption spectra of PyBA are broad and unstructured, lacking vibrational fine structure. The respective absorption maxima (*λ*_max_) are positioned at the border between UV and visible regions, spanning only a narrow range of about 1100 cm^−1^ (362–377 nm) across all investigated environments in this work (Fig. S9, SI). Furthermore, a hypsochromic shift upon increasing solvent polarity indicates negative solvatochromism in accordance with the literature,^29^ as well as results from EOAM. This observation corroborates the more charge-separated structure of PyBA in its ground state, which is also supported by the smaller excited state dipole moment of 6.1 D (S_1_^FC^) with respect to the S_0_ ground state with 9.8 D as determined by EOAM (Table S5, SI). The molar extinction coefficients in all solvents studied are around 44 × 10^3^ M^−1^ cm^−1^, as exemplified by CH_2_Cl_2_ (Fig. 2c).

**Fig. 2:**
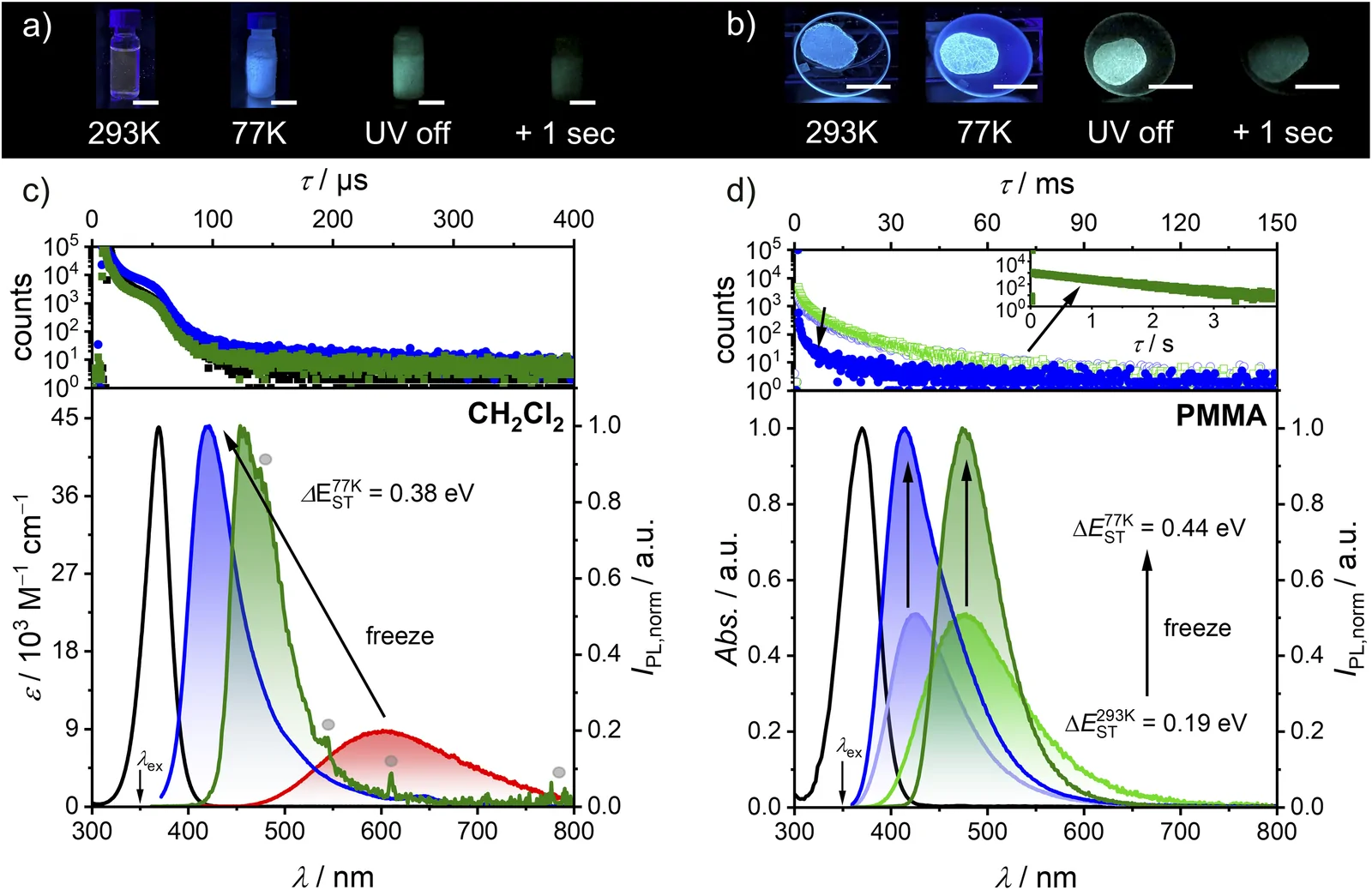
Photographs of PyBA under UV excitation (*λ*_ex_ = 365 nm) in (a) CH_2_Cl_2_ (*c* = 2 × 10^−5^ mM) and (b) a PMMA thin film (<1 wt%) at 293 K and 77 K, respectively, illustrating the fluorescence turn-on effect upon freezing and the persistent phosphorescence at 77 K that remains visible as an afterglow once the UV light is switched off. The scale bar provided next to each photograph corresponds to 10 mm. (c) Absorption (black, *c* ≈ 2 × 10^−5^ M) and emission spectra of PyBA in CH_2_Cl_2_ (*c* ≈ 1 × 10^−6^ M) at 293 K under ambient conditions, showing TICT emission (red), while at 77 K under an argon atmosphere they reveal prompt fluorescence (blue) and phosphorescence (green, measured separately with a time-gated detector). The intensity of the TICT spectrum has been scaled by 0.2 for better visualization of the turn-on effect. Signals marked with a grey circle are attributed to solvent artefacts arising from scattering phenomena, which are visible due to the low signal intensity of the gated measurement. The corresponding time-resolved decays collected at 410 nm (blue) and 460 nm (green) by MCS with a microsecond flash lamp are shown in the upper panel, together with the corresponding instrument response function (IRF, black). (d) Absorption (black) and emission spectra of PyBA in a PMMA thin film (<1 wt%) with prompt fluorescence in blue and phosphorescence in green (measured separately with a gated detector). Measurements performed at 293 K are shown in lighter colours, whereas those recorded at 77 K are shown in darker colours. To highlight the fluorescence turn-on upon freezing, the spectra have been scaled relative to the ratio of their integrated intensities. The corresponding decays recorded by MCS with a microsecond flash lamp are displayed above, using open symbols for the measurements at 293 K collected at 430 nm (light blue) and 500 nm (light green), and filled symbols for the measurements at 77 K collected at 420 nm (dark blue) and 530 nm (dark green), with arrows indicating delayed fluorescence.

### Solvent- and matrix-dependent photophysical properties

The variation in the excited state properties of PyBA is most clearly observed in CH_2_Cl_2_, where three distinct pathways emerge depending on temperature (Fig. 2c). At 293 K, a broad and far red-shifted emission band with its maximum (*λ*_Fl_) located at 603 nm is observed, readily indicating the existence of an energetically relaxed twisted intramolecular charge transfer (TICT) state. Upon cooling to 190 K, deliberately remaining in the condensed phase, the relative intensity of the red-shifted emission band decreases whilst a narrower emission band appears at 420 nm (Fig. S22, SI), suggesting the presence of an activation barrier and partial suppression of the twisting motion at lower temperatures towards the TICT state. The full inhibition of this rotation and thus also of the emergence of the proposed TICT emission was then however only achieved upon freezing the sample. Although the frozen-state experiment is not directly comparable to measurements in solution, it still provides valuable mechanistic insight. At 77 K, prompt emission is observed as a narrow band with a maximum at 420 nm, presumably associated with fluorescence from the ^1^LE state. When time-gated detection is applied, a second emission band becomes apparent at 457 nm, which can presumably be assigned to phosphorescence and thus gives rise to a singlet–triplet gap of 0.38 eV determined from the onsets of the photoluminescence (PL) bands (Fig. 2c). For details regarding the singlet–triplet gap determination, see Table S6, SI. Time-resolved decays collected at both maxima with a microsecond flashlamp *via* multiple channel scaling (MCS) reveal similar profiles and the existence of long-lived components along both emissive pathways with lifetimes of up to 76 µs, which is indicative of delayed fluorescence in addition to phosphorescence. Analogous experiments were conducted in a poly(methyl methacrylate) (PMMA) thin film at <1 wt% of PyBA (Fig. 2d). Sample preparation details are provided in the SI. In PMMA at r.t., the strongly red-shifted emission band associated with the TICT state is not present. Instead, fluorescence and phosphorescence occur already at 293 K, with maxima at 427 nm (Fl) and 476 nm (Ph), respectively, and a singlet–triplet gap of 0.19 eV and thus highlight room temperature phosphorescence (RTP), which was detected separately by applying a time-gated detector. Time-resolved decays recorded at both bands with a microsecond flashlamp *via* MCS again show similar profiles and long-lived components along both emissive pathways with time constants of 16.8 ms. The average prompt fluorescence lifetime, however, was found to be 3.26 ns *via* time-correlated single photon counting (TCSPC) with an EPL laser diode, indicating that delayed fluorescence contributes to the overall excited state dynamics in PMMA. Cooling the sample to 77 K doubles the luminescence intensity under identical measurement conditions and causes slight spectral shifts of both bands, with fluorescence and phosphorescence maxima at 414 nm and 474 nm, respectively. Furthermore, the singlet–triplet gap increases to 0.44 eV, mainly caused by narrowing of both PL spectra. The time-resolved decays collected for both bands at 77 K with a microsecond flashlamp *via* MCS diverge markedly from the ones measured at 293 K. Whilst the long-lived time component along the fluorescence pathway decreases to 2.6 ms, which is indicative of thermally activated delayed fluorescence (TADF), the phosphorescence lifetime increases drastically to 705 ms. Notably, all luminescence spectra shown in Fig. 2 were measured under an argon atmosphere. However, the PMMA thin film measurement at 293 K was additionally repeated under ambient conditions, demonstrating RTP even in the presence of oxygen with a lifetime of 9.04 ms. Furthermore, coating the PMMA thin film with an oxygen barrier consisting of poly(vinyl alcohol) (PVA) further increases the RTP performance with a lifetime of 25.2 ms (Fig. S18, SI). Photographs of the CH_2_Cl_2_ solution under UV light (Fig. 2a) show the turn-on effect upon freezing the sample and finally the persistent phosphorescence after switching off the UV light, giving rise to the afterglow effect. The same applies to the photographs of the PMMA thin film, which differ only in that fluorescence is already visible at 293 K (Fig. 2b). Notably, these photographs were taken under ambient conditions.

To probe the viscosity dependence of PyBA's excited state dynamics, a series of measurements was conducted in environments of increasing viscosity at constant temperature (Fig. 3a). Starting with conventional organic solvents such as CH_2_Cl_2_ and dimethylsulfoxide (DMSO), the series extended to polymeric liquids such as poly(ethylene glycol) 400 (PEG400) and poly(tetrahydrofuran) (P-THF), the highly viscous glycerol (Gly) and finally to a solid solution in PMMA. Although the comparability of solution and solid-state samples is limited, we thereby intended to demonstrate the limits of our rigidity series and to assess a screening method that is still suitable for viscosity-sensitive dyes. Notably, PyBA also exhibits solid-state PL with a PL quantum yield (*Φ*_PL_) of 24% as an amorphous powder (Fig. S14, SI). In low-viscosity regimes, *Φ*_PL_ of PyBA remains below 1% with average fluorescence lifetimes of less than 1 ns, mainly governed by efficient TICT-state formation. With increasing viscosity in the mid-to-high-viscosity range, best demonstrated by a measurement series conducted in binary mixtures of methanol (MeOH) and Gly (Fig. S17, SI), internal rotation is hindered and fluorescence from ^1^LE becomes dominant, resulting in a *Φ*_PL_ of 10.7% in pure Gly. The Förster–Hoffmann analysis from 30.6 to 1317 mPa s yielded an almost linear viscosity dependence with a dye-dependent constant *x* of 0.92, which is a reasonable value for molecular rotor-type dyes.^30^ Moreover, this result suggests that the quantum yield would further increase in more rigid environments. Accordingly, a drastic increase to 55% is observed upon incorporation of PyBA into a PMMA thin film (<1 wt%), along with an elongation of the average lifetime to 3.26 ns. This is also accompanied by a hypsochromic shift of the emission maximum from 603 nm in CH_2_Cl_2_ to 427 nm in PMMA, as well as band narrowing, demonstrating the similarity of the effects resulting from both thin film embedment and decreasing temperatures.

**Fig. 3:**
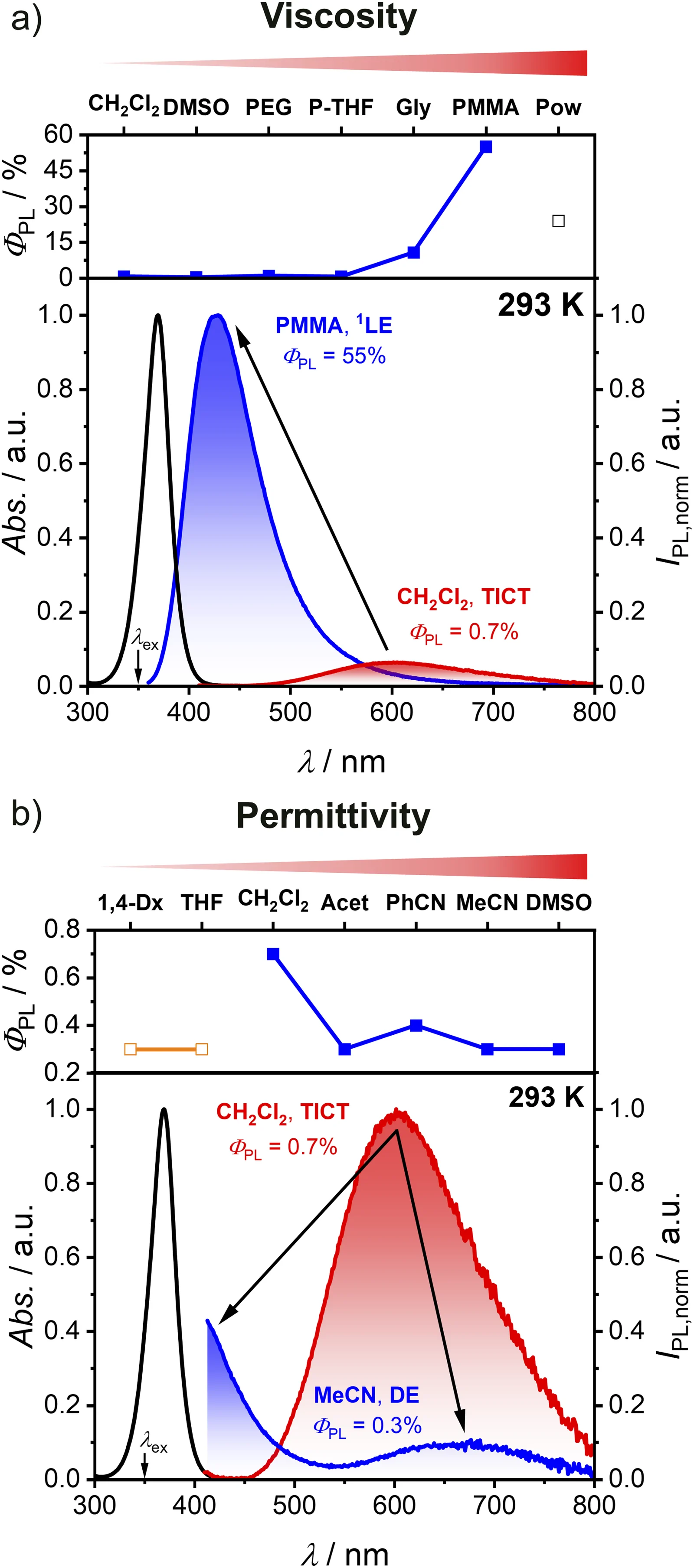
(a) Absorption in CH_2_Cl_2_ (black, *c* ≈ 2 × 10^−5^ M) and emission spectra of PyBA in CH_2_Cl_2_ (red, *c* ≈ 1 × 10^−6^ M) and PMMA (blue, <1 wt%), demonstrating TICT emission and ^1^LE fluorescence, respectively. The emission spectra have been scaled to *Φ*_PL_ with the TICT intensity multiplied by 5 for improved visibility. The plot of *Φ*_PL_ against the environments of different viscosities is provided above, with the solvent- and matrix-dependent ones as blue filled symbols and the powder as an empty black symbol. (b) Absorption in CH_2_Cl_2_ (black, *c* ≈ 2 × 10^−5^ M) and emission spectra of PyBA in CH_2_Cl_2_ (red, *c* ≈ 1 × 10^−6^ M) and MeCN (blue, *c* ≈ 1 × 10^−6^ M), demonstrating TICT emission and dual emission with ^1^LE fluorescence and TICT emission, respectively. The emission spectra have been scaled to *Φ*_PL_. The plot of *Φ*_PL_ against the environments of different polarities is provided above, with the ones measured for PyBA given in filled blue symbols, whereas the ones in less polar solvents measured for PymBA are given as empty orange symbols.

Another series of experiments was performed to elucidate the influence of polarity on the excited state dynamics of PyBA, again at constant temperature (Fig. 3b). The relative permittivity was chosen as the polarity parameter. Accordingly, PyBA was dissolved in CH_2_Cl_2_, acetone (Acet), benzonitrile (PhCN), acetonitrile (MeCN) and DMSO. Due to the limited solubility of PyBA, solvents of lower polarity than CH_2_Cl_2_ could not be included in the screening. To circumvent this limitation and enable a broader polarity range, the methylated derivative PymBA (see SI for more details), which shows otherwise identical optical properties, was prepared and investigated in the less polar solvents 1,4-dioxane (1,4-Dx) and tetrahydrofuran (THF). Interestingly, polarity appears to have only a minor impact on the *Φ*_PL_ of PyBA/PymBA, which remains below 1% across all investigated solvents, with the highest value of 0.7% observed in CH_2_Cl_2_. The average fluorescence lifetime stays similarly low with values of less than 1 ns. However, in all other low-viscous solvents except for CH_2_Cl_2_, pronounced dual emission (DE) was observed, giving rise to both a narrow blue-shifted emission band and a broad red-shifted one, presumably originating from fluorescence of the ^1^LE-state and TICT emission, respectively (Fig. S12 and S15, SI). In this regard, polarity does seem to influence PyBA's behaviour as the relative intensity of the TICT band with regard to fluorescence was usually higher in more polar solvents such as MeCN. Furthermore, to assess the relevance of hydrogen bonding for the excited state dynamics of PyBA, measurements in MeOH were conducted. Surprisingly, all spectral profiles and properties are comparable to other solvents of similar polarity, indicating that hydrogen bonding does not strongly influence the emissive character of PyBA (Fig. S12 and Table S7, SI). The exact solvent parameters used to classify the viscosity and permittivity series are provided in the SI. All photophysical parameters of PyBA discussed in the main manuscript and derived from steady-state spectroscopy or TCSPC/MCS are listed in Table 1, while supplementary ones are provided in the SI (Tables S6–S8).

**Table 1:** Photophysical parameters of PyBA in CH_2_Cl_2_, MeCN and PMMA, obtained from steady-state spectroscopy, giving values for the absorption maximum (*λ*_abs_), fluorescence maximum (*λ*_Fl_), phosphorescence maximum (*λ*_Ph_), Stokes shift (Δ*ν̃*_St_), fluorescence lifetime (*τ*_Fl_), amplitude averaged fluorescence lifetime (*τ*_Fl,avg_), phosphorescence lifetime (*τ*_Ph_), photoluminescence quantum yield (*Φ*_PL_), radiative rate (*k*_r_) and non-radiative rate (*k*_nr_) at both 293 K and 77 K, where available. Notably, the reported rate constants relate to all radiative or non-radiative processes. In MeCN, the decay curves along both fluorescence and TICT pathways were recorded and thus two sets of lifetime parameters are provided (compare Fig. S15)

Solvent	*T*/K	*λ*_abs_/nm	*λ*_Fl_/nm	*λ*_Ph_/nm	Δ*ν̃*_St_/cm	*τ*_Fl,1_ a/ns	*τ*_Fl,2_ a/ns	*τ*_Fl,avg_ a/ns	*τ*_Ph_/ms	*Φ*_PL_/%	*k*_r_ b/10^7^ s^−1^	*k*_nr_ b/10^7^ s^−1^
CH_2_Cl_2_	293	369	603	n.o^c^	10 520	0.45 (56%)	0.79 (44%)	0.55	n.o^c^	0.7	1.27	180
	77	n.d^c^	420	457	n.d^c^	n.d^c^	—	n.d^c^	0.076	n.d^c^	n.d^c^	n.d^c^
MeCN^d^	293	365	407	n.o^c^	2830	<0.20 (97%)	2.38 (3%)	<0.20	n.o^c^	0.3	>1.50	>499
			(+660)^e^		(+12 250)^e^	0.23 (80%)	1.35 (20%)	0.27			1.10	366
PMMA	293	369	427	476	3680	2.63 (42%)	3.93 (58%)	3.26	16.8	55	16.9	13.8
	77	n.d^c^	414	474	n.d^c^	n.d^c^	—	n.d^c^	705	≈100^f^	n.d^c^	n.d^c^

a *τ*_Fl_ was usually best fitted using a bi-exponential fit function, thus giving two components, whose relative contribution to the overall intensity of the decay is given in brackets after each value. Accordingly, the amplitude averaged fluorescence lifetime *τ*_Fl,avg_ was calculated to provide rate constants.

b Determined according to *k*_r_ = *Φ*_PL_*τ*_avg_^−1^ and *k*_nr_ = *τ*_avg_^−1^ − *k*_r_.

c n.d. = not determined; n.o. = not observed.

d The denoted lifetime values in this solvent can only be recognized as upper limits, as such short-lived components are below the instrument response time of the TCSPC setup. Accordingly, the calculated rate constants reflect only lower limits.

e Values corresponding to the TICT emission band. Note that the Stokes shift is formally not correct to calculate with the TICT maximum; it is just provided for comparison with CH_2_Cl_2_.

f Determined by the relative integral ratio of both emission spectra upon excitation at the isosbestic point under identical measurement parameters at different temperatures. This value is therefore estimated based on the relative increase in emission intensity.

### Time-resolved optical spectroscopy

The excited state dynamics of PyBA was investigated using time-resolved fs-transient absorption (fs-TA) spectroscopy in CH_2_Cl_2_ solution and in a PMMA thin film (Fig. 4). In CH_2_Cl_2_, the fs-TA spectra exhibit an initial negative band at ∼500 nm, which decays almost immediately following photoexcitation. Concurrently, excited state absorption (ESA) appears at 650 nm. The negative band is assigned to stimulated emission (SE) from the initially formed excited state. This transient SE band subsequently undergoes a progressive redshift to 600 nm and eventually to ∼670 nm between 0.8 ps and 20 ps as the excited state evolves through structural and solvent relaxation in solution, while ESA is then observed at 480–500 nm. Thereafter, both bands decay gradually within <1 ns. The underlying kinetic processes were analysed using singular-value decomposition (SVD) followed by a global fitting procedure. The fs-TA data were best described with three distinct species, characterized by decay-associated spectra (DAS) with time constants of 0.9, 19 and 450 ps, respectively. Single wavelength kinetics at selected probe wavelengths confirm these time constants (Fig. S26, SI). To further resolve the evolution of ultrafast emissive states, fs-fluorescence up-conversion measurements were performed for PyBA in CH_2_Cl_2_. The decay profiles were best fitted with bi-exponential functions, yielding time constants of 0.4 ps and 62 ps (Fig. S27, SI). In contrast, the fs-TA measurement in PMMA reveals distinct spectral dynamics, characterized by a persistent SE band centred at 470 nm and a weak, broad ESA spanning 550–700 nm that persists throughout the experimental time window (3.5 ns), without noticeable spectral shifts. Global analysis yields bi-exponential kinetics with a fast component of 5 ps and a long component of >3 ns. The blue-shifted and persistent SE band indicates that rotational motion and subsequent structural relaxation are significantly restricted in the rigid matrix. Consequently, the excited state remains in a less-relaxed configuration compared to that in CH_2_Cl_2_, where rapid relaxation gives rise to the progressive redshift of the SE band.

**Fig. 4:**
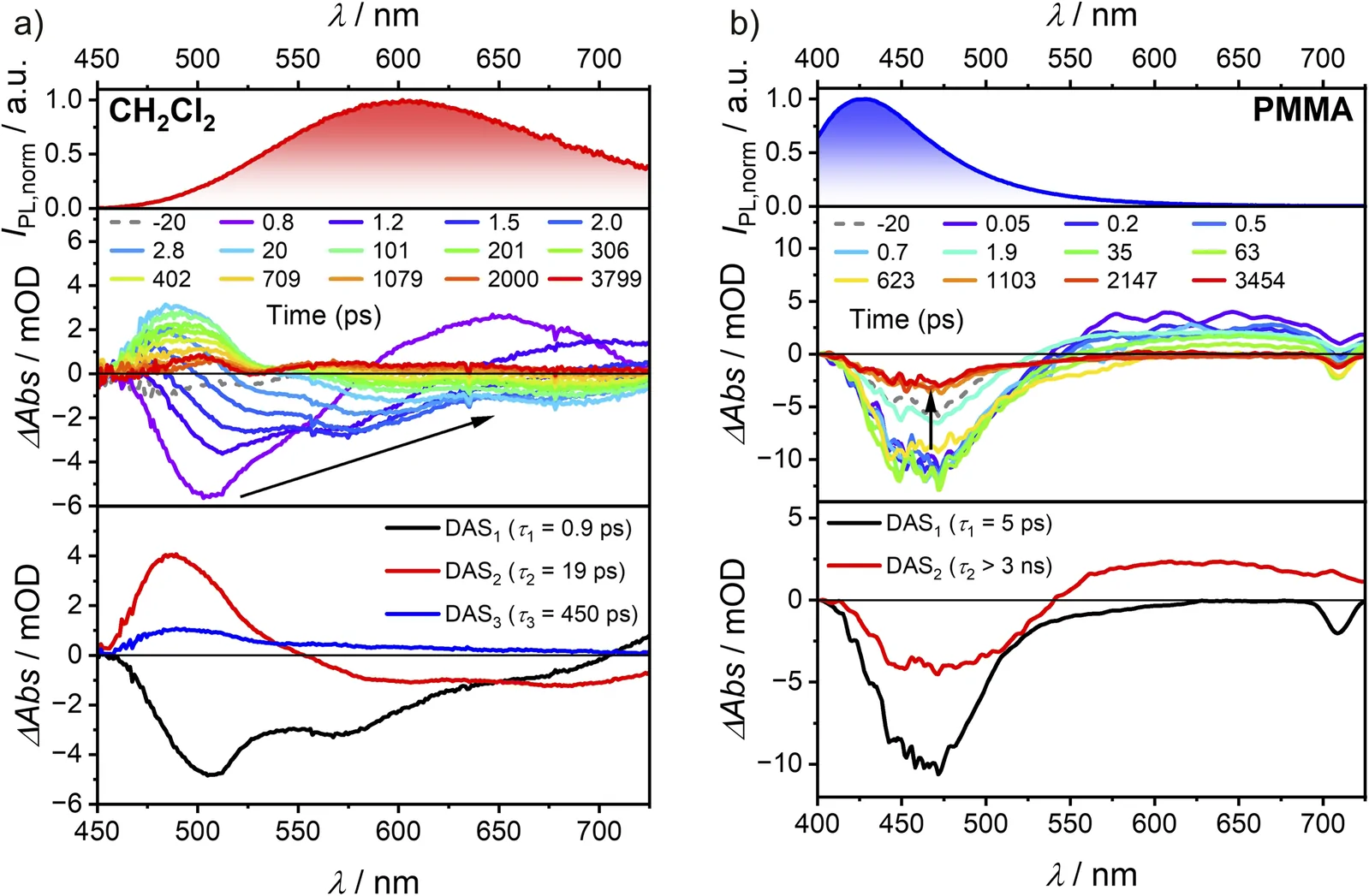
Steady-state emission spectra upon excitation at 350 nm (top panel), fs-TA spectra upon excitation at 370 nm with the respective time delay steps (middle panel) and decay-associated spectra (DAS) with the respective time constants (bottom panel) of PyBA in (a) CH_2_Cl_2_ solution (*c* ≈ 5 × 10^−4^ mM) and (b) PMMA thin film, recorded at 293 K under ambient conditions. The arrows indicate the progression of the stimulated emission bands.

### Quantum-chemical calculations

Ground state structures were first optimized using DFT calculations with ORCA 6 ^31^ at the ωB97X-D3/def2-TZVP level of theory for comparison with the experimentally derived crystal structure and to validate the computational model. This yielded a slightly pre-twisted ground state geometry with a torsional angle (*θ*) of 18° around the central donor–acceptor double bond as the thermodynamically most stable arrangement, which is in good agreement with the experiment. Notably, all calculations employed a conductor-like polarizable continuum model (CPCM) in CH_2_Cl_2_, consistent with the primary experimental solvent used. Furthermore, TD-DFT calculations at the same level of theory were performed to optimize the S_1_ states, revealing a perpendicular arrangement of the two heterocycles constituting PyBA with a torsional angle of 90° around the central double bond, indicating TICT formation. Additionally, an energy gap of 3.37 eV between S_0_ and S_1_ was observed in the almost planar ground-state geometry, which decreases to ∼2 eV in the twisted TICT geometry (Fig. 5, Fig. S28, SI). The (TD)-DFT calculated dipole moments of the ground and excited state structures (16.9 and 11.4 D for S_0_ and S_1_^FC^, respectively) corroborate the trend of the earlier discussed EOAM studies, namely a reduction of the dipole moment upon photoexcitation, which is commonly found for molecules possessing negative solvatochromism.^32^ The same trend was reproduced at a CASSCF(8,8) level of theory (Table S5, SI). The TICT geometry, however, exhibits a more pronounced separation of electron and hole density in the natural transition orbital (NTO) analysis (Fig. S30, SI), consistent with its CT character. Accordingly, the bond length alternation (BLA) analysis along the conjugated chain revealed values of −0.02 and −0.06 Å for the ground and excited states, respectively, suggesting a subtle change in PyBA's electronic structure from the partially zwitterionic resonance structure (Fig. 1b) to a more charge-separated form upon photoexcitation, consistent with the TICT picture. To approximate either low-temperature conditions or viscous environments like a PMMA matrix, the S_1_ geometry of PyBA was constrained to a torsional angle of 30°, whilst relaxing all other degrees of freedom, which was then found to be the model for the emissive state for fluorescence, effectively mimicking rigidification. Notably, as this is only an *ab initio* calculation, the torsional angle of the true emitting species can be anywhere between the Franck–Condon geometry (*θ* = 18°) and the approximated model system (*θ* = 30°). Choosing the angle of 30° is rationalized by the fully relaxed T_1_ geometry, optimized at the same level of theory, which was found to exhibit a torsional angle of 33°. Since phosphorescence was only observed in constrained environments, it was presumed that this is the limit of possible rotational motion under restricted conditions. Otherwise, the TICT geometry is always the thermodynamically most stable conformation in the singlet excited state.

**Fig. 5:**
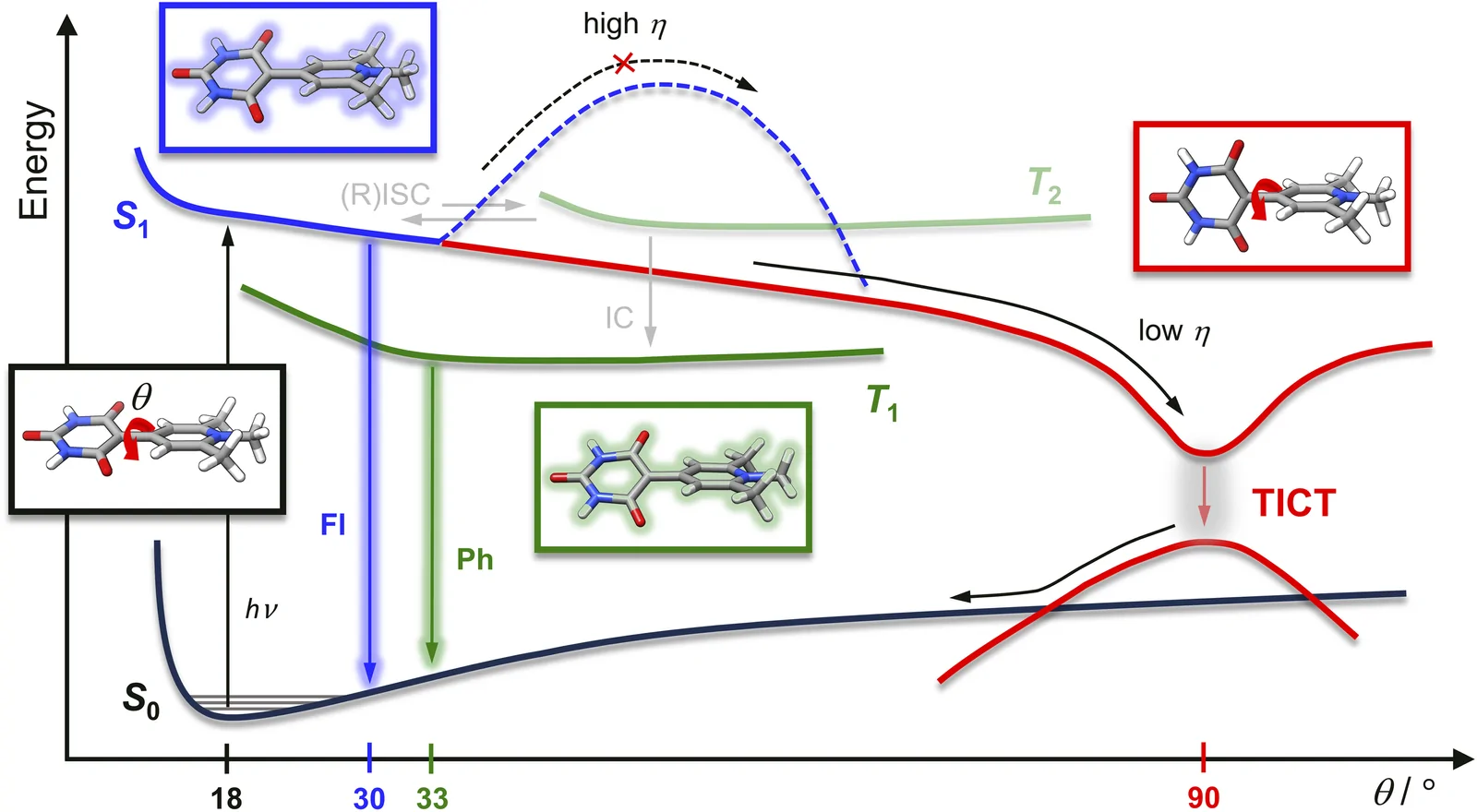
Simplified schematic illustration of the potential energy landscape of PyBA as a function of its torsional angle *θ*. The relative energy level ordering of the three states S_1_, T_1_ and TICT is according to the experimental values extracted from steady-state spectroscopy and values obtained by quantum-chemical calculations but does not depict the absolute values. The corresponding structures of each state are marked with respectively coloured boxes and were obtained by (TD)-DFT. Notably, the structures have been optimized in CH_2_Cl_2_, whilst the energy landscape illustrates the general mechanisms, reliable in all environments. Furthermore, the position and shape of the T_2_ potential is only illustrative and was not verified experimentally or computationally. The potential energy barrier at around 33° torsion towards formation of the TICT state is dependent on the viscosity *η* of the environment.

All four structures were subsequently refined and labelled with the respective energy levels using SA-CASSCF(8,8)/def2-SVP//NEVPT2 with CPCM-CH_2_Cl_2_ solvation. This analysis led to a conclusive picture of the excited state energy ordering with singlet–triplet gaps of 0.25–0.4 eV, depending on the torsional angle, with decreasing energy gaps for more tilted geometries (Fig. S28, SI). Notably, the absolute energy of S_1_ decreases with increasing torsional angle of PyBA with its minimum located at 90° torsion, while S_0_ slightly increases in energy, effectively reducing the S_0_/S_1_ energy gap in tilted geometries. In contrast, the T_1_ state has its energy minimum at 33° torsion, which then increases with increasing torsional angle, revealing an isoenergetic S_1_–T_1_ minimum energy crossing point at the fully perpendicular arrangement of PyBA. To assess the possibility of S_1_–S_0_ non-radiative decay *via* a CI, a minimum energy conical intersection (MECI) point was optimized using mixed-reference spin-flip (MRSF) TD-DFT with the GAMESS 2024 R2 software package^33^ at the BH&HLYP/6-31 + G* level of theory in the gas phase. This analysis reveals that a MECI point between S_1_ and S_0_ can indeed be accessed by PyBA through rotation around the central double bond and pyramidalization of several sp^2^ carbon centres. However, the energy level of this point is more than 0.7 eV above the otherwise highest S_1_ energy, rendering this pathway very unlikely. For more details regarding the above-mentioned energy levels and structures not already depicted in Fig. 5, see SI (Fig. S28).

## Discussion

The preceding steady-state and time-resolved measurements revealed versatile emission behaviour of PyBA, tunable by the viscosity, polarity and temperature of its environment. Three distinct emission bands were identified with prompt fluorescence arising at 410–430 nm, phosphorescence at 460–470 nm and a broad red-shifted feature of a TICT state at 600–650 nm with the exact spectral shift being dependent on the respective solvent. Increasing viscosity and/or decreasing temperature generally resulted in a luminescence turn-on effect, characterized by enhanced quantum yields and longer lifetimes, whereas polarity primarily affected only the spectral position. This behaviour was further investigated with fs-TA spectroscopy, giving rise to matching spectral properties and details on the kinetics of each process. In addition, quantum-chemical calculations established geometries for all emissive states and assigned corresponding energy levels. Together, these results allow us to propose a potential energy scheme for PyBA's excited state dynamics (Fig. 5), which is elaborated below.

### Excited state mechanisms

Upon photoexcitation, PyBA relaxes from its excited Franck–Condon state to the S_1_ minimum, from which an almost barrierless transition to the excited TICT state occurs in low viscosity environments by torsional motion. The existence of this TICT state is confirmed by multiple experiments conducted according to established methods on this subject.^2,3^ First, the quantum-chemical calculations revealed the fully perpendicular arrangement of PyBA with a 90° torsional angle between donor and acceptor to be the most stable geometry in the S_1_ state (Fig. 5). Additionally, the temperature-dependent fluorescence study conducted in CH_2_Cl_2_ in the range of 190–293 K, deliberately remaining in the condensed phase and avoiding freezing for a better comparison of temperature effects, revealed a progression from the TICT emission towards ^1^LE emission, accompanied by an increase in *Φ*_PL_ to 3% at 190 K and band narrowing (Fig. S22, SI). This result explicitly demonstrates the existence of an energetic barrier and the relevance of twisting motions for the excited state dynamics of PyBA. Finally, time-resolved spectroscopy in CH_2_Cl_2_ resolved the TICT formation dynamics (Fig. 4). The SE band centred at 500 nm with a time constant of 0.9 ps in DAS1 corresponds to the formation of an initial intramolecular charge transfer (ICT) state. The subsequent progression of the SE band towards the range of 550–700 nm in DAS2 with a time constant of 19 ps, accompanied by pronounced ESA at 480 nm, is consistent with the formation of a TICT state, which is in agreement with previous reports on donor–acceptor systems exhibiting TICT formation.^34,35^ This interpretation is further substantiated by the pronounced emission broadening in steady-state fluorescence spectra and the appearance of dual emission in MeCN and other solvents (Fig. 3).^36^ Such spectral signatures are characteristic for CT-mediated excited-state relaxation pathways.^37–39^ DAS3 exhibits a weak ESA band with a time constant of 450 ps, corresponding to the lifetime of the relaxed TICT (S_1_) state. The time constant is consistent with the lifetime component of 450 ps that was determined by TCSPC. Furthermore, fs-TA data recorded in MeCN (Fig. S25, SI) additionally supports the TICT picture, as similar spectral profiles have been observed. However, the overall excited state lifetime of PyBA decreases from 450 ps in CH_2_Cl_2_ to 52 ps in MeCN, corroborating the strong influence of solvent polarity on CT dynamics.

On the potential energy surface (PES) of PyBA, an energy barrier is encountered along the relaxation pathway from the S_1_ state to the TICT state, depicted schematically at a torsional angle of around 33° (Fig. 5). The position of this barrier is rationalized by the fact that phosphorescence only occurs in constrained environments, and this torsional angle is still accessible by the relaxed T_1_ geometry. However, the barrier can also be located at higher degrees of torsion. This barrier emerges only in rigid environments like a PMMA matrix and/or at low temperatures, dictating whether TICT formation is possible due to restricted rotational motions. Consequently, in corresponding measurements, PyBA was “trapped” in its constrained S_1_ state with *θ* ≈ 30° and fluorescence from ^1^LE (and phosphorescence, delayed fluorescence) was observed rather than emission from the TICT state (Fig. 2 and 3). This observation is consistent with the temperature-dependent emission measurements in CH_2_Cl_2_, the measurements conducted in the MeOH/Gly series and the fs-TA spectra in the PMMA thin film, which show no SE band progression indicative of TICT formation (Fig. 4). Interestingly, steady-state measurements in PMMA reveal two additional phenomena. The observed blue shift of the emission bands upon freezing likely results from increased polarity of the medium and concomitant stabilization of the emissive state, as decreasing temperatures typically result in increasing permittivities.^10^ Notably, fluorescence is more strongly affected than phosphorescence, indicating greater stabilization of S_1_ relative to T_1_. Furthermore, the increase in luminescence quantum yield upon freezing suggests that some torsional and vibrational motions remain possible in the PMMA matrix at elevated temperatures, consistent with the free volume concept.^40,41^ Consequently, only the combination of matrix rigidification and cryogenic cooling fully suppresses large amplitude motions responsible for non-radiative decay.

Polarity, on the other hand, also affects the PES of PyBA as it shifts the energetic balance between the S_1_ and TICT minima. It thereby determines the relative contributions of their fluorescence, which manifests as dual emission at 410–450 nm and 600–700 nm in low-viscosity media, respectively (compare Fig. 3 and S12, SI). More polar solvents such as MeCN or PhCN likely stabilize the more charge-separated TICT state more strongly than the S_1_ state, resulting in more pronounced TICT emission. The exceptions observed in this study are DMSO, where fluorescence from ^1^LE remains dominant despite the high polarity, and CH_2_Cl_2_, where almost exclusively TICT emission is detected. The former may be rationalized by the increased viscosity and strong specific solvation of DMSO, which together can reduce the efficiency of twisting motions. In contrast, the behaviour observed in CH_2_Cl_2_ may arise from its distinct solvation characteristics, including comparatively strong dispersion interactions and high polarizability.

In addition, emission originating from the triplet manifold following intersystem crossing (ISC) at cryogenic temperature or in PMMA plays a significant role in the excited state dynamics of PyBA, if the otherwise ultrafast TICT transition is inhibited. The emission band arising at 460–470 nm (Fig. 2) is unambiguously assigned to phosphorescence, as the spectral separation from fluorescence exceeds that expected for delayed fluorescence. Furthermore, experimentally determined singlet–triplet gaps agree well with the theoretically calculated ones, supporting this assignment. The mechanism underlying ISC was further elucidated by computing the squared reduced spin–orbit coupling matrix elements (SOCME) and difference densities of relevant singlet and triplet states. In the S_0_ geometry, T_2_ is energetically close to S_1_ and the squared SOCME between these states is not zero with 11 (cm^−1^)^2^. Furthermore, the El-Sayed rule is respected between them, with the orbital and transition character changing from mixed LE/CT and an n → π* transition in S_1_ to predominantly LE and a π → π* transition in T_2_ (Fig. S29a, SI). In addition, the carbonyl groups of the acceptor unit seem to play an important role in enabling ISC as well, having perpendicularly oriented p-orbitals in S_1_ and T_2_. In contrast, S_1_ and T_1_ have practically the same character and zero SOC was calculated between them. The same trends were obtained in the relaxed T_1_ geometry, featuring even a slightly higher SOC between S_1_ and T_2_ (Fig. S29b, SI). Notably, SOC between S_1_ and T_2_ is again zero in the perpendicular TICT arrangement. Consequently, in a rigid environment that constrains the geometry of PyBA close to the ground state or T_1_ structure and suppresses other non-radiative deactivation pathways, ISC to T_2_ and subsequent phosphorescence from T_1_ can occur. Delayed fluorescence does seem to contribute as well to the excited state dynamics of PyBA, as long-lived components are observed along both fluorescence and phosphorescence decay pathways, indicating reverse ISC. This observation was substantiated with temperature-dependent emission and lifetime measurements in PMMA (Fig. S23, SI). The experiment illustrates broadening of the gated emission spectra upon heating, which indicates a stronger contribution of delayed fluorescence to the overall emission profile, while the relative intensity of phosphorescence decreases. Furthermore, temperature-dependent decay profiles measured between 293 K and 77 K demonstrate increased lifetimes for delayed fluorescence upon heating, while the phosphorescence lifetime drastically decreases. Such a phenomenon is common for chromophores exhibiting thermally activated delayed fluorescence (TADF).^42^ The ISC rate was further modulated by addition of ethyl iodide to CH_2_Cl_2_, exploiting the heavy-atom effect to enhance spin–orbit coupling. The measurements indeed revealed a strong increase in phosphorescence intensity, accompanied by an extended lifetime from 76 µs in pure CH_2_Cl_2_ to 318 ms in the ethyl iodide mixture at cryogenic temperatures under an argon atmosphere (Fig. S20, SI). However, at 293 K, only TICT emission was observed, confirming that the triplet state is accessible only when ultrafast TICT formation is fully prohibited, even with enhanced ISC. The experimental result of PyBA exhibiting RTP in PMMA, even under ambient conditions, and (weak) phosphorescence in a frozen CH_2_Cl_2_ solution is particularly interesting, as phosphorescence of MC dyes was previously only achieved in the crystalline solid state^43^ or realized with the help of external stimuli like Dexter-type triplet–triplet energy transfer from a suitable host,^44,45^ thereby circumventing the otherwise often inefficient ISC pathway.

### Comparison with other fluorophores

For most MC dyes, non-radiative relaxation occurs *via* CIs, typically leading to photoisomerization to a different product or direct tunnelling from S_1_ to S_0_, as demonstrated *e.g.* for DCBT,^20^DCM,^46^ and MC540,^47,48^ (Fig. 1). This behaviour often appears counterintuitive with respect to the energy gap law, something they have in common with stilbenoid-type compounds.^49^ Such excited state dynamics are often triggered by the inherent “floppy” nature of these molecules and have been covered in a comprehensive review article.^50^ Owing to its unique structure, PyBA does not follow these mechanisms due to the absence of the flexible polymethine chain connecting donor and acceptor moieties. Instead, it exhibits spectral features typically observed for TICT dyes like the archetypical DMABN,^2^ but also other MC dyes like TCF,^51^ the broader class of fluorescent molecular rotors^52^ and even some hemicyanines.^53^ Especially the latter two classes of compounds, including prominent candidates like BODIPY dyes,^54,55^ DCDHF derivatives,^56,57^ or Thioflavin T and X (ThT and ThX),^58,59^ that are structurally comparable to PyBA, emphasize the generality of the concept presented in this work, as they also exhibit ultrafast decay in non-viscous environments *via* rotational motions, which are hindered in constrained environments and thus result in a fluorescence turn-on. What distinguishes PyBA, however, is its pronounced tunability, the existence of an emissive triplet state and its ability to cover a broad range of the visible spectrum in emission. Moreover, with its strong fluorescence turn-on effect and high photostability (compare Fig. S21, SI), PyBA is expected to constitute a promising candidate for probing and imaging applications, as has been demonstrated for other TICT chromophores.^60^ In this context, the measurements conducted in MeOH provide a useful reference point, since protic solvents and hydrogen-bonding environments appear to have only a limited influence on the excited state dynamics of PyBA. Lastly, for some MC dyes, aggregation-induced emission enhancement (AIEE)^61–63^ is of major relevance. However, this was deemed to be negligible for PyBA, as it did not show any signs of aggregation even at a 2 mM concentration in a CH_2_Cl_2_ or MeCN solution (Fig. S11, SI).

## Conclusions

Here, we have introduced a zeromethine merocyanine dye PyBA with exciting multimodal emission properties and resolved its excited state landscape, revealing tunable and versatile luminescence spanning (delayed) fluorescence, TICT emission and phosphorescence depending on its environment and accessibility to torsional motions. Especially the latter distinguishes PyBA from other MC dyes, as RTP is observable even under ambient conditions in a restricted environment like a PMMA matrix. These findings position PyBA as a promising scaffold for the further exploration of structure–property relationships in MC dyes with accessible triplet-state emission. Furthermore, the strong fluorescence turn-on effect up to 55% quantum yield at r.t. in viscous media, coupled with <1 ps ICT formation in dynamic environments and thus high photostability due to reduced photochemical degradation *via* reactive oxygen species, positions PyBA as an interesting probe for bioimaging. The latter was elucidated by a photodegradation assay while irradiating at 370 nm over the course of 11 h in CH_2_Cl_2_ solution (Fig. S21, SI). Consequently, ongoing work targets incorporation of PyBA into oligonucleotides to serve as a luminescent DNA label, potentially expanding RTP applications in live-cell imaging.

## Author contributions

F. W. conceived the project and acquired funding. J. F. performed the synthesis and characterization, as well as steady-state spectroscopy and data interpretation. P. V. and A. L. performed time-resolved optical spectroscopy and acquired and scrutinized the corresponding data. Me. S. carried out the quantum-chemical calculations. Ma. S. performed the electro-optical absorption measurements and organized the thin-film preparation. J. F. and F. W. drafted the original version of the manuscript with the input and approval of all authors.

## Conflicts of interest

There are no conflicts to declare.

## Supplementary Material

SC-OLF-D6SC05772A-s001

## Data Availability

The data underlying this study are available in the Supporting Information and in Zenodo, an open research repository, at https://doi.org/10.5281/zenodo.19470049. CCDC 2544677 contains the supplementary crystallographic data for this paper.^64^ Supplementary information (SI) is available. See DOI: https://doi.org/10.1039/d6sc05772a.
