# Supplementary material for: Exploring the excited state mechanisms of a viscosity-sensitive zeromethine merocyanine dye emitter
Source: Chem Sci. 2026 Oct 12. Online ahead of print. doi: 10.1039/d6sc05772a (PMC13686152; doi:10.1039/d6sc05772a)
Supplement: Supplementary file 1 [file SC-OLF-D6SC05772A-s001.pdf]

Supplementary Information (SI) for Chemical Science.
This journal is © The Royal Society of Chemistry

Electronic Supplementary Information

Exploring the Excited State Mechanisms of a Viscosity-Sensitive Zeromethine Merocyanine Dye Emitter

Julian Fichtner,[a] Preetika Verma,[b] Menyhárt Sárosi,[a,c] Matthias Stolte,[a,c] Yapamanu Adithya Lakshmanna*[b] and Frank Würthner*[a,c]

[a] Institut für Organische Chemie, Julius-Maximilians-Universität Würzburg, Am Hubland, 97074 Würzburg, Germany

[b] School of Chemistry, Indian Institute of Science Education and Research Thiruvananthapuram, Vithura, Thiruvananthapuram 695551, India.

[c] Center for Nanosystems Chemistry (CNC), Julius-Maximilians-Universität Würzburg, Theodor-Boveri-Weg, 97074 Würzburg, Germany.

E-mail: adithya@iisertvm.ac.in; frank.wuerthner@uni-wuerzburg.de

Table of Contents

1 Materials and Methods S2
2 Synthetic Procedures S6
3 NMR Spectroscopy S7
4 Mass Spectrometry S9
5 Structural Analysis S10
5.1 Single Crystal X-Ray Crystallography S10
5.2 Electro-Optical Absorption Measurements S13
6 Optical Spectroscopy S14
6.1 UV-Vis Absorption S14
6.2 Steady State Fluorescence Spectroscopy S16
6.3 Time-resolved fs-Transient Absorption S27
6.4 Time-resolved fs-Fluorescence Up-Conversion S28
7 Quantum-Chemical Calculations S28
8 Supporting References S30

1 Materials and Methods

Chemicals and Solvents

All standard chemicals and solvents were obtained from commercial suppliers and used without further purification, if not stated otherwise. Organic solvents for spectroscopic studies were of spectroscopic grade and used as received, except for poly(ethylene glycol) 400 (PEG400), poly(tetrahydrofuran) (P-THF) with an average molecular weight of 1000 g mol^{-1} and glycerol, which were of technical, chromatography and molecular biology grade, respectively. The poly(methyl methacrylate) (PMMA) utilised in this work had an average molecular weight of 15000 g mol^{-1}. Notably, dichloromethane used in phosphorescence studies was distilled prior to use to exclude luminescence phenomena arising from impurities and stabilizers. For classification of the solvents in the viscosity and polarity series, the parameters listed below in Table S1 were applied.

Table S1. Summary of the dynamic viscosities (η), dielectric constants (ε_r) and refractive indices (n) of the used solvents. The values have been extracted either from a list of Sigma-Aldrich[S1] or from other references, which are then given after each corresponding value. 1,4-Dx = 1,4-dioxane, THF = tetrahydrofuran, Acet = acetone, PhCN = benzonitrile, MeCN = acetonitrile, DMSO = dimethylsulfoxide, MeOH = methanol, Gly = glycerol.

Solvent	η^a / mPa s	ε_r^a	n^a
1,4-Dx	1.44 (15 °C)	2.21 (25 °C)	1.422
THF	0.55	7.60	1.407
CH_2Cl_2	0.45 (15 °C)	9.08	1.424
Acet	0.32	20.7 (25 °C)	1.359
PhCN	1.24 (25 °C)	26.0[S2]	1.528
MeOH	0.55	32.6 (25 °C)	1.329
MeCN	0.37	37.5	1.344
DMSO	2.24	46.7[S2]	1.479
PEG400	~120[S3]	11.6[S4]	1.470[S5]
P-THF	~440 (30 °C)[S6]	~5.00[S7]	1.466[S8]
Gly	1317[S9]	42.5[S9]	1.474[S2]

^{a} T = 20 °C, unless specified otherwise.

Synthetic Details

For monitoring of the reaction progress, thin layer chromatography (TLC) was carried out, using silica gel pre-coated TLC plates ALUGRAM®Xtra SIL G/UV254 (layer thickness: 0.2 mm) purchased from *Macherey-Nagel*. Visualization was accomplished by irradiation with UV light at 254 nm and 365 nm. Melting points were measured with the melting point apparatus SMP50 by *Cole-Parmer Stuart* with an applied heating rate of 2 K min^{-1} and are uncorrected.

Thin Film Preparation

Preparation of the PMMA thin films was achieved by drop-casting 80 µL of a 1:1 mixture of a solution of the respective dye in dichloromethane ($c = 2 \times 10^{-5}$ M) and a solution of PMMA in dichloromethane (c = 0.05 g mL^{-1}) on quartz glass substrates from *Hellma*, resulting in a highly diluted doping ratio of <1wt% dye in PMMA matrix. The solutions were filtered through a 0.22 µm syringe filter prior to use.

While drop-casting, the substrates were heated to 80 °C by a precision heating stage from *Harry Gestigkeit GmbH* to quickly evaporate the solvent. The samples used for fluorescence spectroscopy were drop-casted once (film thickness ≈ 1.2–1.3 µm), while the samples for measurements of the absolute quantum yield were drop-casted five times to achieve different thickness values of the samples and thus, different optical densities. Notably, if drop-casting once, a smooth and even film is produced. Contrary, when drop-casting multiple times, the film morphology changes due to non-uniform evaporation and resolvation, which is also why these films have been used only for determining the photoluminescence quantum yield.

15 thin films have been prepared independently by this method to ensure good sample-to-sample reproducibility, always yielding identical absorption profiles and fluorescence properties.

The oxygen-blocking barrier was prepared by first dissolving poly(vinyl alcohol) (PVA, M_{avg} ≈ 85000 – 120000 g mol^{-1}) in water by stirring at 70 °C for 2 h (10 mg mL^{-1}). The PMMA film was then evenly coated with the PVA solution and dried at 95 °C for 1 h. To remove residual oxygen underneath the barrier before performing the luminescence studies, the film was illuminated with a UV flashlamp (λ_{ex} = 365 nm) for 1 min.

NMR Spectroscopy

All NMR spectra were measured at 295 K if not otherwise mentioned. ^{1}H and ^{13}C spectra were measured at Avance III HD 400 spectrometers. Chemical shifts δ are given in ppm and were referenced to the residual solvent signals as internal standards (DMSO-d_6: ^{1}H = 2.50, ^{13}C = 39.52).[S10] Coupling constants *J* were reported in Hz. For all multiplicities, following abbreviations were used: s = singlet, t = triplet, se = sextet and m = multiplet. Spectral assignments were verified by 2D NMR experiments, including HSQC, HMBC and COSY.

Mass Spectrometry

High resolution mass spectra were measured on a Bruker micrOTOF-Q III spectrometer from *Bruker Daltonics GmbH* using the atmospheric pressure chemical ionization – direct insertion probe (APCI-DIP) technique in positive-ion mode.

X-Ray Crystallography

Single crystals of **PyBA** suitable for X-ray crystallographic analysis were obtained by slow evaporation of methanol from a highly concentrated solution. Single crystal X-ray data were collected at 220 K on a Bruker D8Quest Kappa Diffractometer with a Photon II CPAD are detector and multi-layered mirror monochromated CuKα radiation. The structures were solved using SHELXT,[S11] expanded with Fourier techniques and refined with the SHELX software package.[S12] All non-hydrogen atoms were refined anisotropically. Hydrogen atoms were included in the structure factor calculation on geometrically idealized positions.

UV-Vis Spectroscopy

UV-Vis absorption spectra were measured at 293 K with V-670 and V-770 spectrophotometers from *Jasco* equipped with a JASCO PAC-743R Auto Peltier 6/8-cell changer system for temperature control. Standard quartz glass cuvettes with 1 or 10 mm path length of *Hellma* were used. Thin film samples were measured at a Perkin-Elmer Lambda 950 spectrometer using appropriate sample holders.

Electro-Optical Absorption Measurements (EOAM):

Dipole moments of the ground state μ_g and the dipole moment differences $\Delta\mu = \mu_e - \mu_g$ (μ_e = excited state dipole moment) of chromophores were determined by EOAM with a home-built instrument.[S13] The single beam spectrometer is composed of a Xenon lamp as light source in combination with a double monochromator (*Horiba Jobin Yvon*) followed by a Hanle depolarizer as well as a Glan polarizer to obtain linear polarized light with appropriate spectral bandwidth ($\Delta\lambda$ < 1 nm). This polarized light passes through a sample solution ($OD \leq 1.2$) in a stainless-steel sample cell, with two flat electrodes at a distance of about 3 mm. The difference of absorption of a solution with ($\varepsilon^E (\varphi,\tilde{\nu})$) and without ($\varepsilon (\tilde{\nu})$) an externally applied electric field **E** was measured with light parallely ($\varphi = 0°$) and perpendicularly ($\varphi = 90°$) polarized to the direction of **E**.[S14,S15] For uniaxial phases, induced in a solution by both an alternating and a constant electric field of about 3×10^6 V m^{-1}, the dichroism "$\varepsilon^E (\varphi,\tilde{\nu}) - \varepsilon (\tilde{\nu})$" depends on the orientational order of the molecules due to their ground state dipole moment μ_g, the shift of the absorption band proportional to the dipole moments difference $\Delta\mu$, and on the electric field dependence of the electric transition dipole moment $\mu_{eg}(\mathbf{E})$. The linear combination L_p^{tu}, corrected by the contribution of the first ($t(\tilde{\nu})$) and second ($u(\tilde{\nu})$) derivative of the UV-Vis-NIR spectrum, should be independent of $\tilde{\nu}$ and proportional to μ_g^2 in case of a uniformly polarized transition band. UV-vis-NIR spectra, required for the evaluation of the integral absorption (μ_{eg}^2) as well as the determination of $t(\tilde{\nu})$ and $u(\tilde{\nu})$, were recorded with a *Perkin-Elmer Lambda 950* spectrophotometer at 298 K. All EOA measurements were carried out in anhydrous 1,4-dioxane.

Fluorescence Spectroscopy

Fluorescence studies were carried out on a FLS980-D2D2-ST spectrometer from *Edinburgh Instruments Ltd., Inc.* and corrected against the photomultiplier sensitivity and lamp intensity. All spectra were recorded under magic angle conditions (54.7°) at 293 K and a 90° geometry, if not stated otherwise, using a standard sample holder with Peltier element and high precision quartz glass cuvettes with a path length of 10 mm from *Hellma*. The samples were prepared by the high dilution method (OD ≤ 0.05) to minimize reabsorption and inner-filter effects. Thin film and powder samples were measured at a 22.5° geometry using the front-face setup without magic angle conditions. Delayed fluorescence and phosphorescence spectra were measured with a microsecond flash lamp µF2 upon gating of the detector with a gate delay of 0.1 ms and a gate width of 9.9 ms. Short PL lifetimes (<50 ns) were determined by *Time Correlated Single Photon Counting* (TCSPC) using an EPL picosecond pulsed laser diode with an excitation wavelength of 378 nm and a pulse width of 78 ps under magic angle conditions. For long PL lifetimes (>50 ns), a microsecond flash lamp µF2 was used for the *Multichannel Scaling* (MCS) method without magic angle conditions and the usage of polarizers, as long-lived components arose only in frozen or solid-state samples. The fitting of the data was carried out using the tail-fit-routine supplied by *Edinburgh Instruments Ltd., Inc.* Values below 1 ns have also been corrected against the instrument response function (IRF), which was collected by using Ludox as a scatterer, employing the recursive-fit-routine of the FAST software supplied by *Edinburgh Instruments Ltd., Inc.*. Temperature-dependent emission spectra were recorded with the Oxford Instruments cryostat Optistat DN with the temperature controller Mercury iTC under an argon atmosphere. The samples were not degassed prior to freezing.

Quantum Yield Determination

Quantum yields were measured absolutely on a Hamamatsu Absolute PL Quantum Yield Measurement System *CC9920-02*. Liquid samples were prepared with an $OD \approx 1$. Values greater than 1% were corrected for reabsorption following the procedure introduced by Ahn *et al*.[S16]

Femtosecond Transient Absorption Spectroscopy

The fs-transient absorption (fs-TA) experiments were carried out using a Ti:sapphire amplifier system (*Spectra-Physics*), which produces fundamental laser pulses centred at 800 nm, with a pulse duration of 100 fs, pulse energy of approximately 5 mJ, and a repetition rate of 1 kHz. The output beam was split into two parts: a major portion was used to pump an optical parametric amplifier (OPA) to generate excitation pulses, while a minor fraction was focused onto a sapphire plate to produce a white-light continuum. This continuum was further divided into two beams: a probe beam and a reference beam. The OPA provided excitation pulses in the UV region, centred at 370 nm, with a pulse duration of ~100 fs and an energy output of ~10 µJ per pulse at 1 kHz. The UV pump excites the sample to an excited state. In the fs-TA setup, the pump and probe beams were spatially and temporally overlapped in a 1.0 mm path-length quartz cuvette mounted on a computer-controlled XY translation stage (Z being the beam axis), allowing continuous sample translation to mitigate photodamage. The pump polarization was kept at a magic angle relative to the probe to eliminate polarization anisotropy. The transmitted probe light was dispersed by a spectrometer and detected by a dual–channel photodiode array. To extract the excited state absorption signal, the pump beam was modulated with a mechanical chopper at 500 Hz, enabling differential measurements at each delay time. All measurements were performed using pump pulses with approximately 500 nJ of energy, focused to a ~100 µm spot on the sample.[S17]

Femtosecond Fluorescence Up-Conversion

Fluorescence up-conversion measurements were performed using a commercial FOG-100 setup (*CDP Systems*). The excitation source was the second harmonic (at 400 nm) of the fundamental output from a Mai Tai–HP laser, which delivers pulses centred at 800 nm with a duration of ~100 fs and operates at a repetition rate of 80 MHz. Upon excitation, the sample's fluorescence was spatially and temporally overlapped with the residual 800 nm beam in a nonlinear β-barium borate (BBO) crystal to generate a sum–frequency signal, representing the time-resolved up-converted fluorescence. This up-converted signal was directed through a double monochromator for spectral selection and is subsequently detected by a highly sensitive photomultiplier tube (PMT) coupled to a photon-counting system.

Computational Details

Ground and excited state geometries were optimized at the ωB97X-D3/def2-TZVP level of theory within the density functional theory (DFT) framework. Relaxed and constrained S_1 structures as well as the relaxed T_1 geometry were optimized within the time-dependent (TD) framework. Using the resulting structures, SA-CASSCF(8,8)/def2-SVP//NEVPT2 single-point calculations were carried out to refine the ground and excited sate energy levels. A conductor-like polarizable continuum model (CPCM) was used throughout to approximate CH_2Cl_2 solvent effects. These calculations were carried out with ORCA 6.[S18] The S_0/S_1 minimum energy conical intersection (MECI) was located with mixed-reference spin-flip (MRSF) TD-DFT at the BH&HLYP/6-31+G* level in the gas phase, as implemented in GAMESS 2024 R2.[S19] Molecular structures and natural transition orbitals (NTOs) were rendered and analysed using UCSF ChimeraX.[S20]

2 Synthetic Procedures

The target compounds **PyBA** and **PymBA** were synthesized according to a literature-known procedure[S21] by adjusting the alkylamine reagent to the desired length of the alkyl chain, as demonstrated in Scheme S1. Whilst starting material **1** is commercially available and was used as received, **2** had to be synthesized prior to usage as described elsewhere.[S22] Intermediate pyran derivatives **3**[S23] and **4**[S24] are literature-known as well and were prepared accordingly.

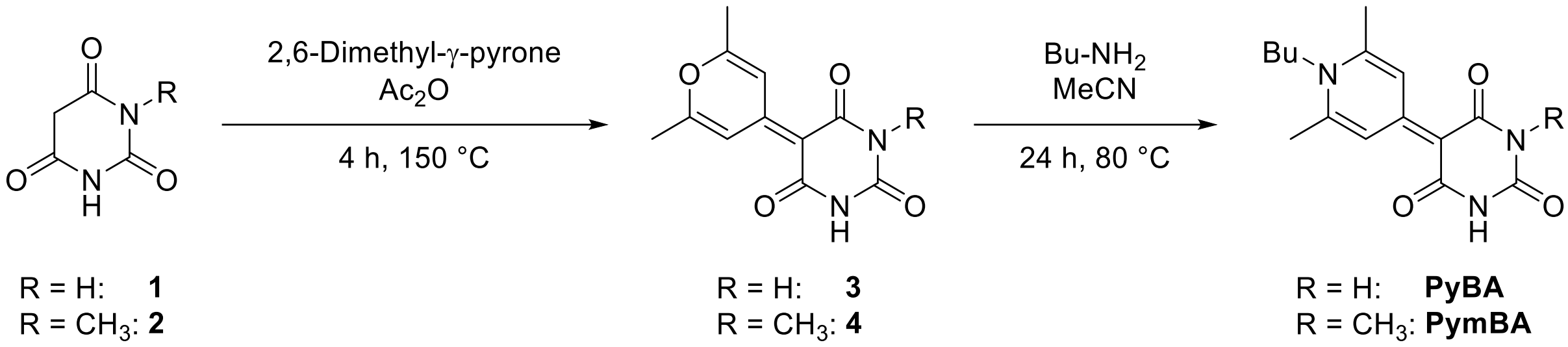

Scheme S1. General synthetic scheme for the preparation of **PyBA** and **PymBA**.

5-(1-Butyl-2,6-dimethylpyridin-4(1*H*)-ylidene)pyrimidine-2,4,6(1*H*,3*H*,5*H*)-trione (PyBA)

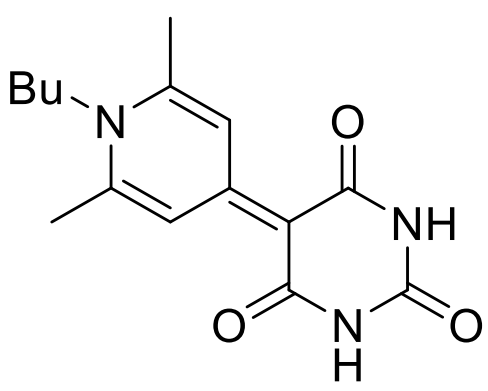

PyBA

Pyran derivative **3** (500 mg, 2.13 mmol, 1.00 eq.) and *n*-butylamine (632 µL, ρ = 740 mg/mL, 6.40 mmol, 3.00 eq.) were suspended in acetonitrile (10 mL) and stirred for 24 h at 80 °C. After cooling to r.t., the precipitate was filtrated and washed with ethanol (50 mL). The product was dried for 5 h at 45 °C under high vacuum and obtained as a pale-yellow solid.

Yield: 564 mg (1.95 mmol, 91%) of a pale-yellow solid.

M.p.: decomp. at 328 °C.

^{1}H NMR (400 MHz, DMSO-d_6, 295 K): δ = 9.90 (s, 2H), 8.82 (s, 2H), 4.16 – 4.12 (m, 2H), 2.59 (s, 6H), 1.72 – 1.64 (m, 2H), 1.41 (se, ^3J = 7.3 Hz, 2H), 0.95 (t, ^3J = 7.3 Hz, 3H) ppm.

^{13}C NMR (101 MHz, DMSO-d_6, 295 K): δ = 165.2, 153.5, 150.3, 148.8, 119.4, 86.6, 49.3, 30.3, 20.6, 19.4, 13.4 ppm.

HRMS (APCI-DIP, pos. mode): (*m*/*z*) calculated for $C_{15}H_{20}N_3O_3^+$ [(M+H)$^+$]: 290.1499, found: 290.1498.

5-(1-Butyl-2,6-dimethylpyridin-4(1*H*)-ylidene)-1-methylpyrimidine-2,4,6(1*H*,3*H*,5*H*)-trione (PymBA)

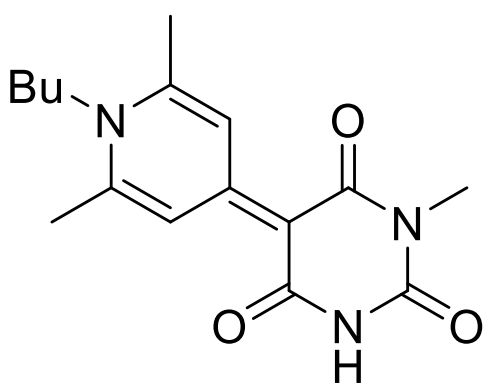

PymBA

Pyran derivative **4** (496 mg, 2.00 mmol, 1.00 eq.) and *n*-butylamine (593 µL, ρ = 740 mg/mL, 6.00 mmol, 3.00 eq.) were suspended in acetonitrile (10 mL) and stirred for 24 h at 80 °C. After cooling to r.t., the precipitate was filtrated and washed with ethanol (30 mL). The raw product was further purified by recrystallization from ethanol (50 mL) and was subsequently dried for 5 h at 45 °C under high vacuum to obtain the product as a yellow solid.

Yield: 465 mg (1.53 mmol, 77%) of a yellow solid.

M.p.: 282–284 °C.

^{1}H NMR (400 MHz, DMSO-d_6, 295 K): δ = 10.14 (s, 1H), 8.83 (s, 2H), 4.17 – 4.13 (m, 2H), 3.08 (s, 3H), 2.60 (s, 6*H*), 1.73 – 1.65 (m, 2H), 1.42 (se, ^3J = 7.4 Hz, 2H), 0.96 (t, ^3J = 7.4 Hz, 3H) ppm.

^{13}C NMR (101 MHz, DMSO-d_6, 295 K): δ = 164.2, 163.6, 153.7, 150.8, 149.0, 119.8, 86.8, 49.4, 30.2, 26.4, 20.6, 19.4, 13.4 ppm.

HRMS (APCI-DIP, pos. mode): (*m*/*z*) calculated for $C_{16}H_{22}N_3O_3^+$ [(M+H)$^+$]: 304.1656, found: 304.1655.

3 NMR Spectroscopy

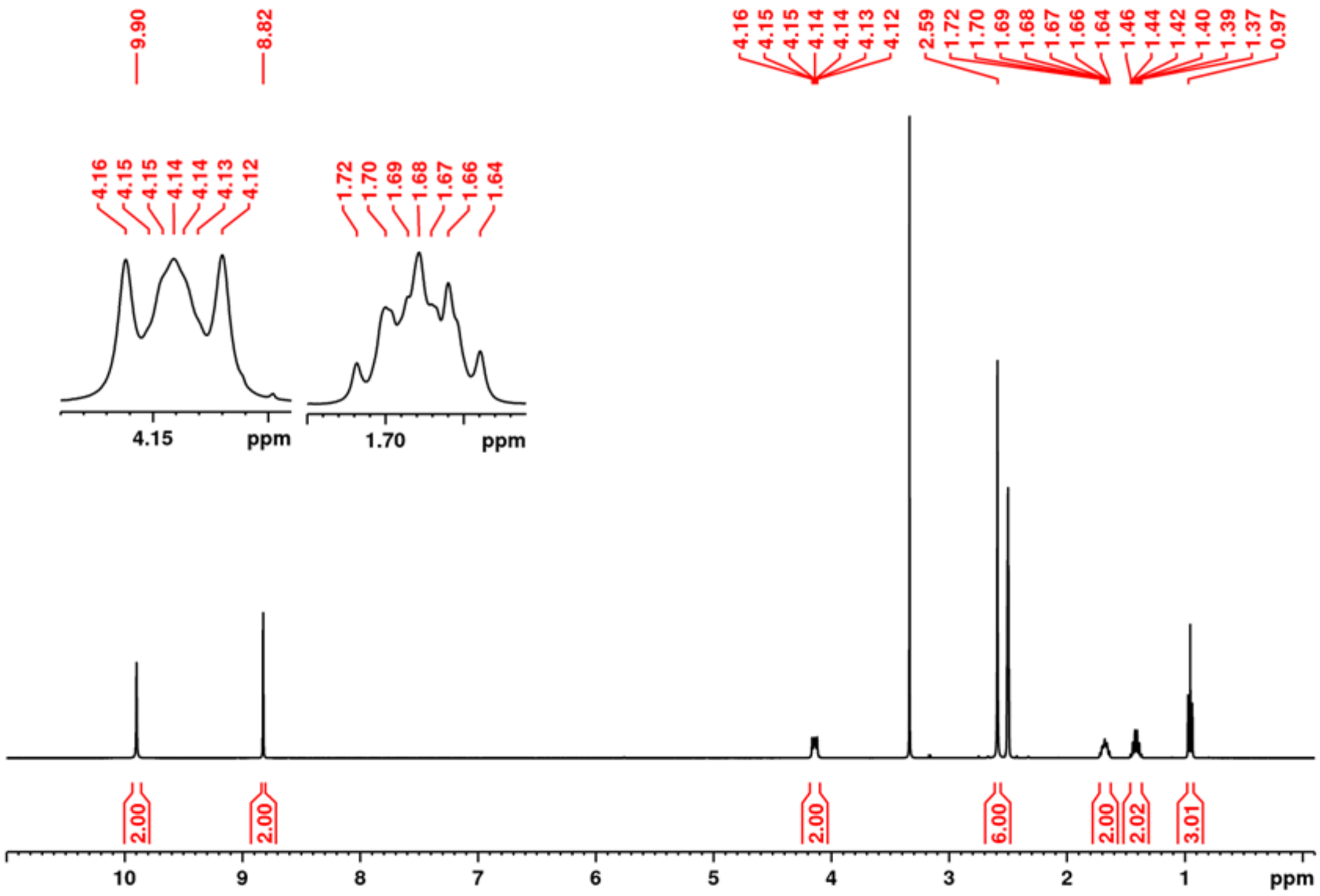

Fig. S1. ^{1}H NMR spectrum (400 MHz, DMSO-d_6, 295 K) of **PyBA**.

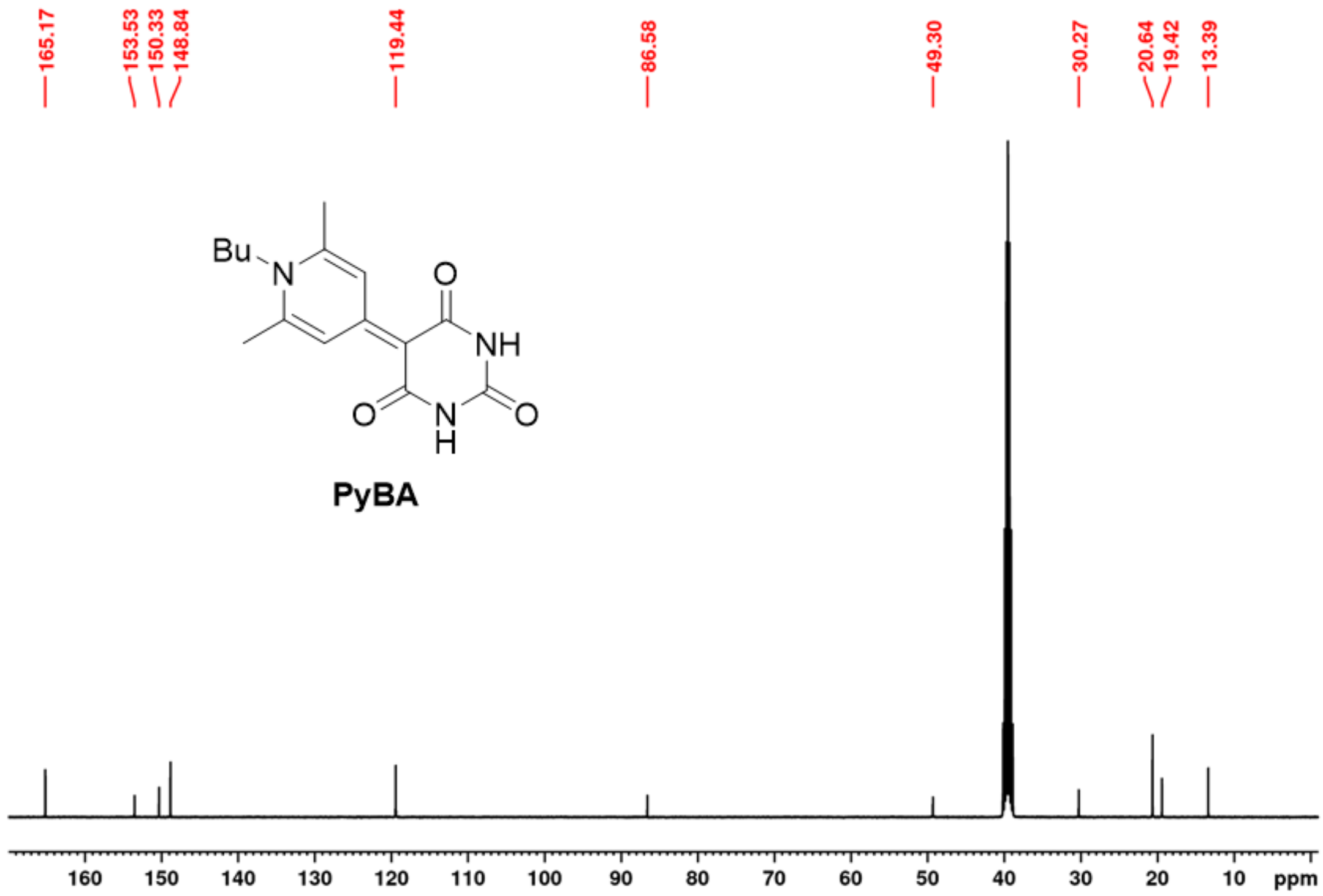

Fig. S2. ^{13}C NMR spectrum (101 MHz, DMSO-d_6, 295 K) of **PyBA**.

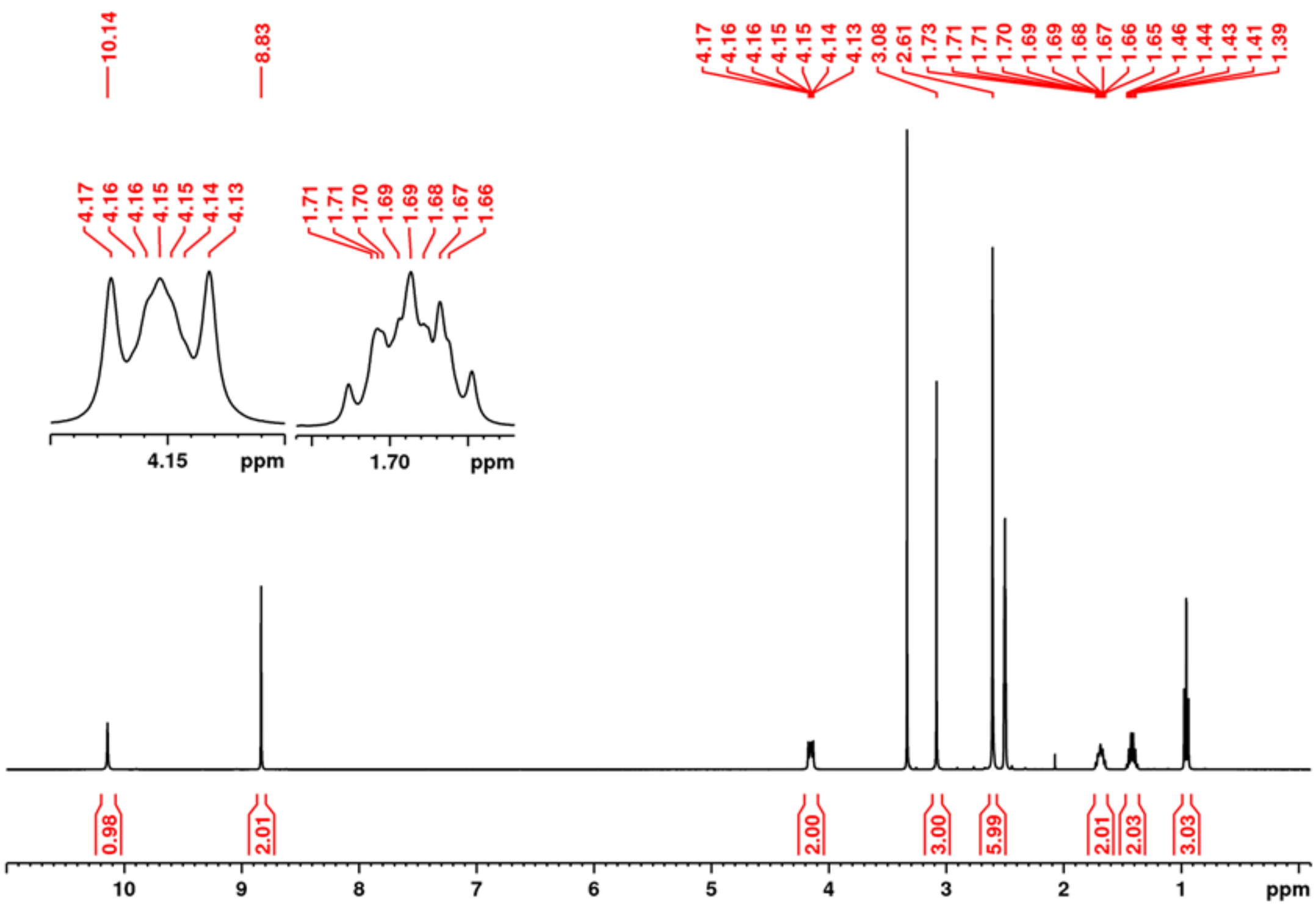

Fig. S3. ^{1}H NMR spectrum (400 MHz, DMSO-d_6, 295 K) of **PymBA**.

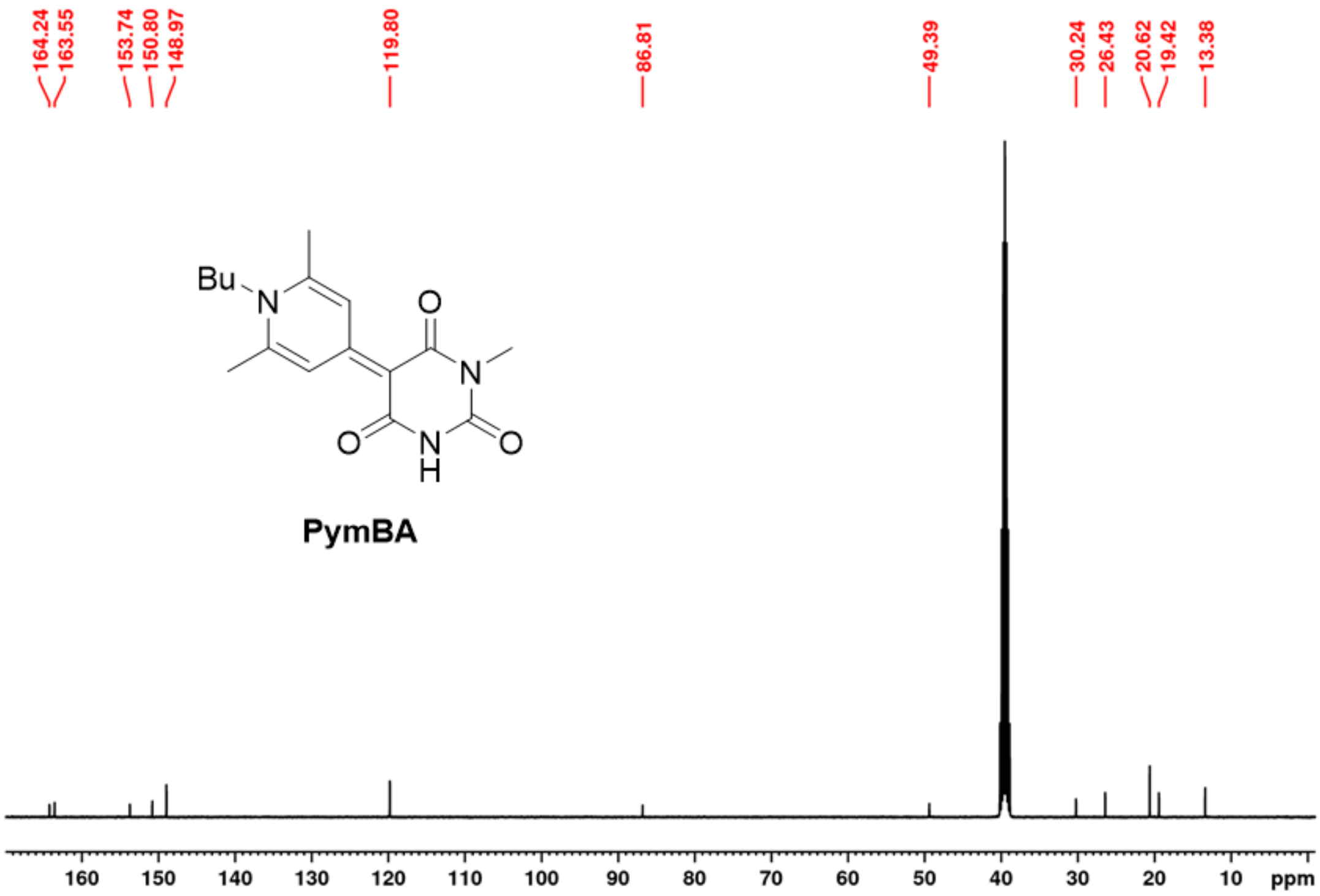

Fig. S4. ^{13}C NMR spectrum (101 MHz, DMSO-d_6, 295 K) of **PymBA**.

4 Mass Spectrometry

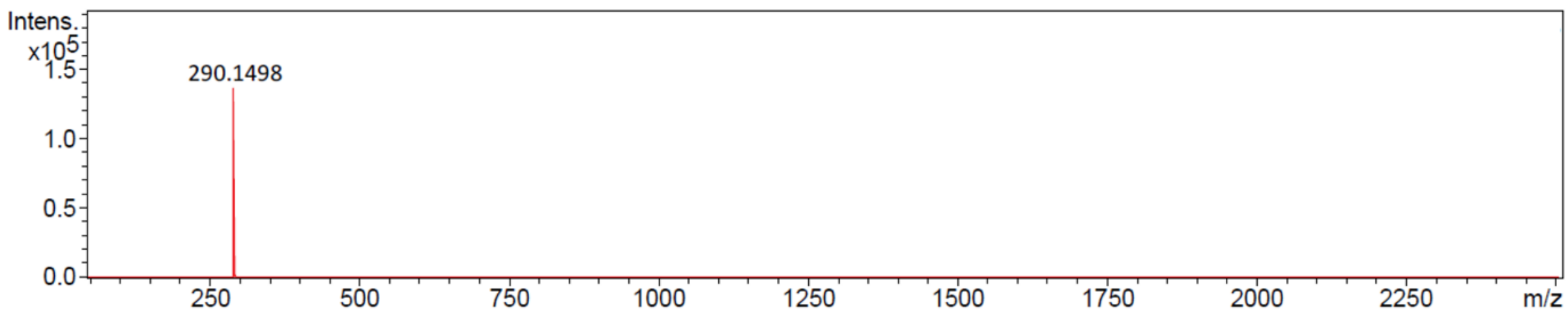

Fig. S5. APCI-DIP mass spectrum of **PyBA** in positive-ion mode.

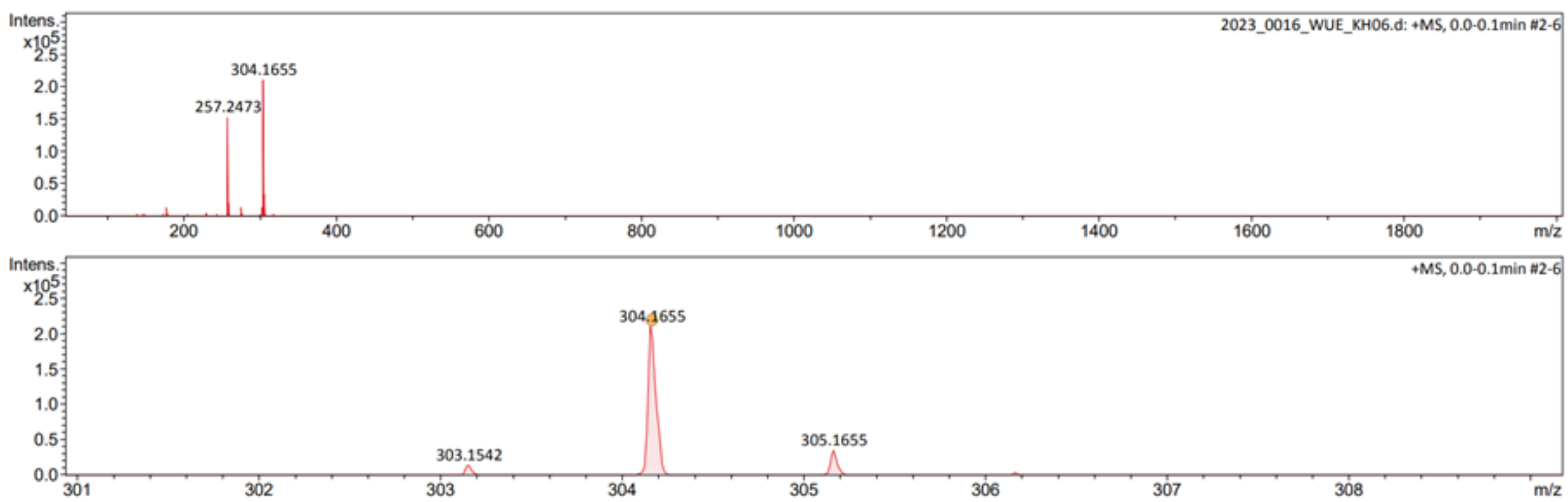

Fig. S6. APCI-DIP mass spectrum of **PymBA** in positive-ion mode.

5 Structural Analysis

5.1 Single Crystal X-Ray Crystallography

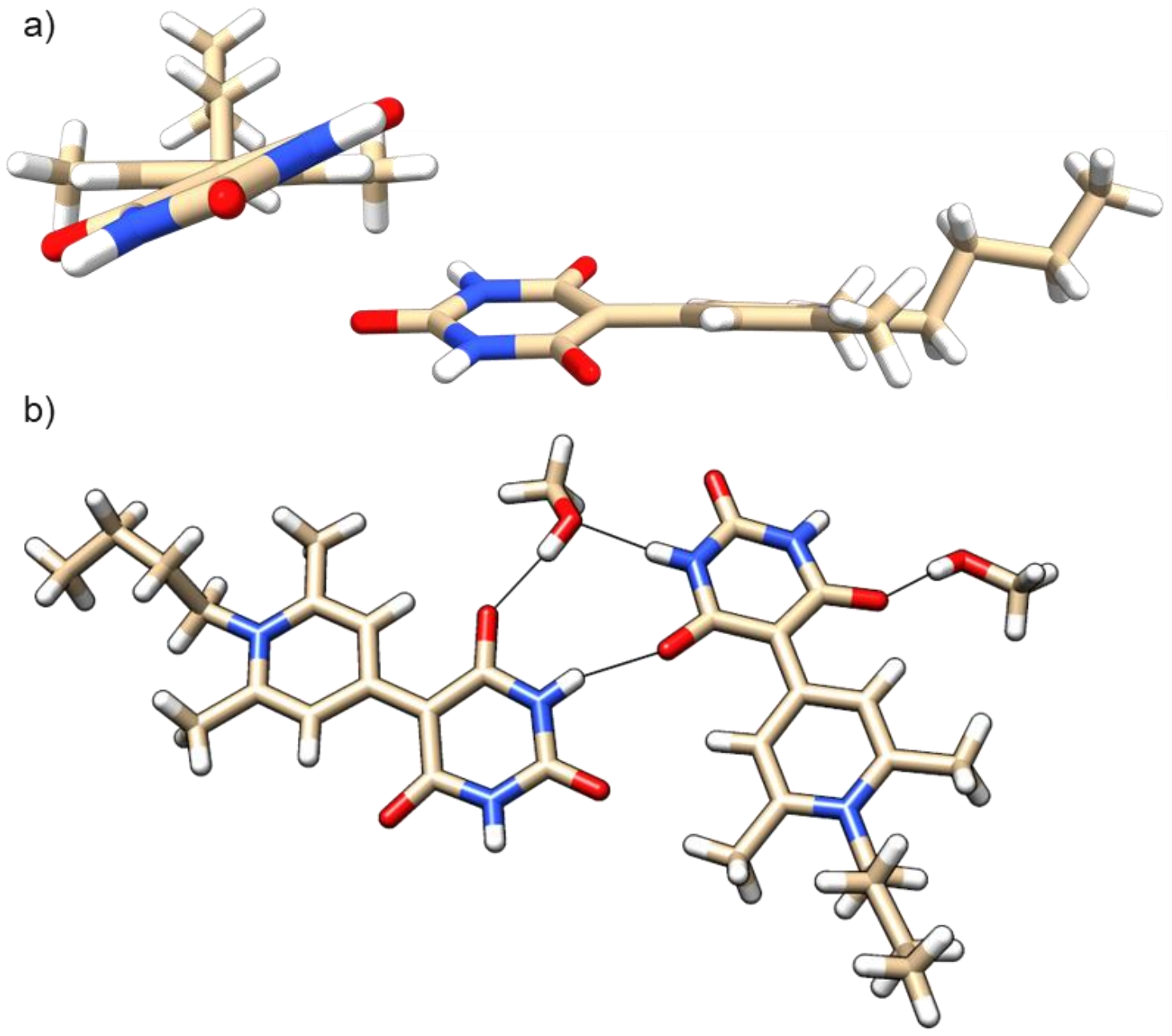

Fig. S7. a) Front- and side-view on to the π-surface of **PyBA** in the crystal structure. b) Crystal packing arrangement of **PyBA**, demonstrating hydrogen bonding to neighbouring molecules as well as surrounding methanol.

Table S2. Crystal data and structure refinement for **PyBA**.

Compound	**PyBA**
CCDC	2544677
Emipirical formula	$C_{16}H_{23}N_3O_4$
Formula weight / g mol^{-1}	321.37
Temperature / K	220(2)
Wavelength / Å	1.54179
Crystal system	Monoclinic
Space group	P2(1)/c
Unit cell dimensions	a = 13.0718(17) Å
	b = 34.036(4) Å
	c = 7.8894(12) Å
	α = 90°
	β = 105.672(6)°
	γ = 90°
Volume / Å^3	3379.6(8)
Z	8

Calculated density / Mg m^{-3}	1.263
Absorption coefficient / mm^{-1}	0.755
F(000)	1376
Theta range for data collection / °	2.596 to 72.263
Limiting indices	$-16 \le h \le 16$, $-41 \le k \le 42$, $-9 \le l \le 9$
Reflections collected	63933
Reflections unique	6652 [R(int) = 0.0271]
Completeness to theta = 67.679°	99.9%
Absorption correction	Semi-empirical from equivalents
Minimal / maximal transmission	0.6194 / 0.7536
Refinement method	Full-matrix least-squares on F^2
Data / Restraints / Parameters	6652 / 0 / 425
Goodness-of-fit on F^2	1.068
Final R indices [$I > 2\sigma(I)$]	R_1 = 0.0531, wR_2 = 0.1519
R indices (all data)	R_1 = 0.0561, wR_2 = 0.1549
Extinction coefficient	n/a
Largest difference peak and hole / e Å^3	0.654 / –0.355

Table S3. Atomic coordinates (× 10^{-4}) and equivalent isotropic displacement parameters (Å^2 × 10^3) for **PyBA**. U(eq) is defined as one third of the trace of the orthogonalized U_{ij} tensor

	x	y	z	U(eq)
O11	3321(1)	4421(1)	8205(2)	59(1)
O21	2558(1)	3156(1)	6734(3)	76(1)
O31	-231(1)	3952(1)	6417(2)	42(1)
N11	2900(1)	3795(1)	7484(2)	44(1)
N21	1184(1)	3570(1)	6624(2)	44(1)
N31	338(1)	5435(1)	7408(2)	40(1)
C11	1475(1)	4260(1)	7304(2)	36(1)
C21	2597(1)	4179(1)	7689(2)	40(1)
C31	2237(1)	3484(1)	6929(3)	47(1)
C41	753(1)	3935(1)	6787(2)	35(1)
C51	1087(1)	4658(1)	7379(2)	36(1)
C61	21(1)	4745(1)	7331(2)	39(1)
C71	-334(1)	5125(1)	7357(2)	40(1)
C81	1378(1)	5367(1)	7477(2)	43(1)
C91	1744(1)	4990(1)	7481(2)	43(1)
C101	-1473(1)	5202(1)	7303(3)	52(1)
C111	2110(2)	5709(1)	7511(3)	59(1)
C121	-54(1)	5845(1)	7399(2)	43(1)
C131	73(2)	6008(1)	9241(3)	52(1)
C141	-126(2)	6449(1)	9214(3)	58(1)
C151	-1257(2)	6563(1)	8363(4)	84(1)
O12	5019(1)	3490(1)	7829(2)	45(1)

O22	7686(1)	4281(1)	7393(2)	62(1)
O32	8100(1)	2987(1)	6493(2)	54(1)
N12	6391(1)	3864(1)	7674(2)	41(1)
N22	7845(1)	3628(1)	6897(2)	43(1)
N32	5177(1)	2013(1)	6680(2)	46(1)
C12	6486(1)	3169(1)	7151(2)	37(1)
C22	5906(1)	3499(1)	7547(2)	36(1)
C32	7331(1)	3949(1)	7317(2)	43(1)
C42	7505(1)	3240(1)	6846(2)	39(1)
C52	6042(1)	2776(1)	7020(2)	39(1)
C62	6672(1)	2435(1)	7164(2)	44(1)
C72	6247(1)	2064(1)	7006(2)	45(1)
C82	4526(1)	2333(1)	6546(3)	50(1)
C92	4947(1)	2705(1)	6733(3)	47(1)
C102	6961(2)	1713(1)	7183(3)	57(1)
C112	3350(2)	2274(1)	6158(5)	79(1)
C122	4712(2)	1612(1)	6441(3)	53(1)
C132	4614(2)	1421(1)	8106(3)	73(1)
C142	4221(2)	995(1)	7683(4)	78(1)
C152	4077(3)	773(1)	9182(5)	111(1)
O13	5452(1)	4483(1)	9002(2)	60(1)
C13	5632(2)	4849(1)	8269(4)	74(1)
O14	9704(1)	2998(1)	4976(3)	73(1)
C14	10040(2)	2615(1)	4806(5)	90(1)

Table S4. Anisotropic displacement parameters (Å^2 × 10^3) for **PyBA**.

	U_{11}	U_{22}	U_{33}	U_{23}	U_{13}	U_{12}
O11	28(1)	47(1)	96(1)	-11(1)	9(1)	-3(1)
O21	42(1)	39(1)	146(2)	-7(1)	22(1)	7(1)
O31	28(1)	38(1)	60(1)	-2(1)	14(1)	-2(1)
N11	27(1)	40(1)	64(1)	3(1)	10(1)	4(1)
N21	32(1)	33(1)	68(1)	-1(1)	14(1)	-1(1)
N31	40(1)	33(1)	46(1)	1(1)	10(1)	0(1)
C11	29(1)	36(1)	43(1)	1(1)	10(1)	0(1)
C21	31(1)	39(1)	50(1)	1(1)	11(1)	0(1)
C31	35(1)	36(1)	71(1)	3(1)	14(1)	4(1)
C41	31(1)	36(1)	41(1)	3(1)	13(1)	0(1)
C51	32(1)	36(1)	41(1)	1(1)	10(1)	-1(1)
C61	33(1)	34(1)	50(1)	0(1)	13(1)	-2(1)
C71	36(1)	37(1)	47(1)	0(1)	13(1)	-1(1)
C81	38(1)	38(1)	52(1)	1(1)	10(1)	-5(1)
C91	31(1)	41(1)	57(1)	1(1)	12(1)	-2(1)
C101	41(1)	39(1)	81(1)	-1(1)	23(1)	3(1)
C111	45(1)	41(1)	89(2)	3(1)	15(1)	-8(1)
C121	47(1)	33(1)	48(1)	5(1)	11(1)	3(1)
C131	64(1)	40(1)	52(1)	-1(1)	12(1)	4(1)
C141	63(1)	41(1)	73(1)	-5(1)	22(1)	4(1)

C151	74(2)	69(2)	108(2)	-11(1)	21(1)	22(1)
O12	33(1)	40(1)	67(1)	-3(1)	21(1)	-1(1)
O22	40(1)	36(1)	114(1)	-6(1)	29(1)	-7(1)
O32	41(1)	40(1)	88(1)	0(1)	30(1)	5(1)
N12	30(1)	34(1)	61(1)	-5(1)	15(1)	-1(1)
N22	28(1)	38(1)	66(1)	0(1)	18(1)	-2(1)
N32	54(1)	34(1)	50(1)	-3(1)	16(1)	-6(1)
C12	31(1)	34(1)	48(1)	2(1)	12(1)	1(1)
C22	29(1)	36(1)	43(1)	1(1)	9(1)	-1(1)
C32	30(1)	37(1)	62(1)	-1(1)	12(1)	-1(1)
C42	31(1)	37(1)	51(1)	2(1)	12(1)	2(1)
C52	37(1)	36(1)	45(1)	1(1)	13(1)	0(1)
C62	40(1)	37(1)	54(1)	2(1)	12(1)	2(1)
C72	51(1)	36(1)	46(1)	1(1)	12(1)	2(1)
C82	46(1)	40(1)	69(1)	-4(1)	21(1)	-6(1)
C92	39(1)	36(1)	69(1)	-1(1)	18(1)	-2(1)
C102	63(1)	38(1)	65(1)	-3(1)	7(1)	8(1)
C112	49(1)	46(1)	145(2)	-16(1)	33(1)	-13(1)
C122	67(1)	36(1)	54(1)	-7(1)	13(1)	-12(1)
C132	95(2)	59(1)	63(1)	1(1)	19(1)	-30(1)
C142	90(2)	56(1)	89(2)	-1(1)	25(1)	-21(1)
C152	159(3)	78(2)	96(2)	0(2)	31(2)	-53(2)
O13	34(1)	51(1)	94(1)	-18(1)	19(1)	-2(1)
C13	65(1)	49(1)	117(2)	-21(1)	38(1)	-9(1)
O14	55(1)	62(1)	113(1)	-24(1)	42(1)	-14(1)
C14	80(2)	71(2)	130(2)	-34(2)	45(2)	-4(1)

5.2 Electro-Optical Absorption Measurements

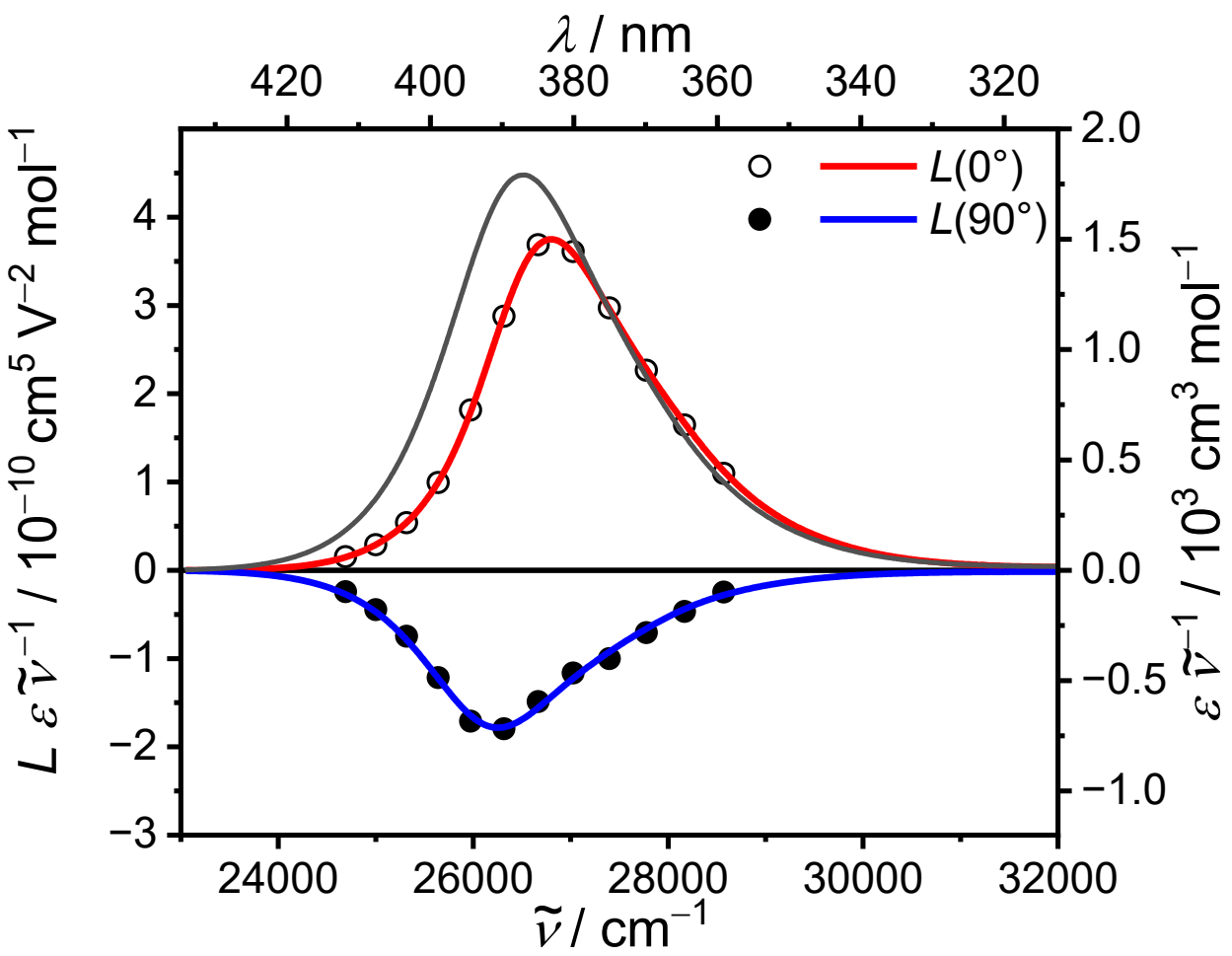

Fig. S8. EOAM (symbols) and UV-Vis absorption (black line) spectra of merocyanine dye **PyBA** in 1,4-dioxane at 298 K. The EOAM signal is shown for light parallelly (0°; red) and perpendicularly (90°; blue) polarized to the direction of the externally applied electric field **E** and includes the corresponding multilinear regressions (solid red and blue lines).

Table S5. Structural properties of **PyBA** including the bond length alternation (BLA) in the ground (g) and excited state (e), as well as the ground (μ_g) and excited state (μ_e) dipole moments and the transition dipole moment (μ_{eg}), provided by different quantum-chemical methods as well as UV/Vis and electro-optical absorption measurements (EOAM). Since EOAM can only determine μ_e of the locally excited Franck-Condon state (S_1^{FC}), the quantum-chemical methods were accordingly applied to calculate μ_e after vertical photoexcitation, but before geometrical relaxation occurs, for better comparison. Notably, the theoretically calculated dipole moments are typically overestimated[S25] and thus only demonstrate the qualitative trend. μ_{eg} reflects the value for the transition from S_0 to S_1^{FC}. The resonance parameter (c^2) has been calculated from the UV/Vis and EOAM-derived values.

Method	BLA_g / Å	BLA_e / Å	μ_g / D	μ_e (S_1^{FC}) / D	μ_{eg} / D	c^2
EOAM	-	-	9.8	6.1	6.4	0.64
(TD)-DFT	–0.02	–0.06	16.9	11.4	7.6	-
CASSCF	–0.02	–0.06	14.2	12.4	6.1	-

6 Optical Spectroscopy

6.1 UV-Vis Absorption

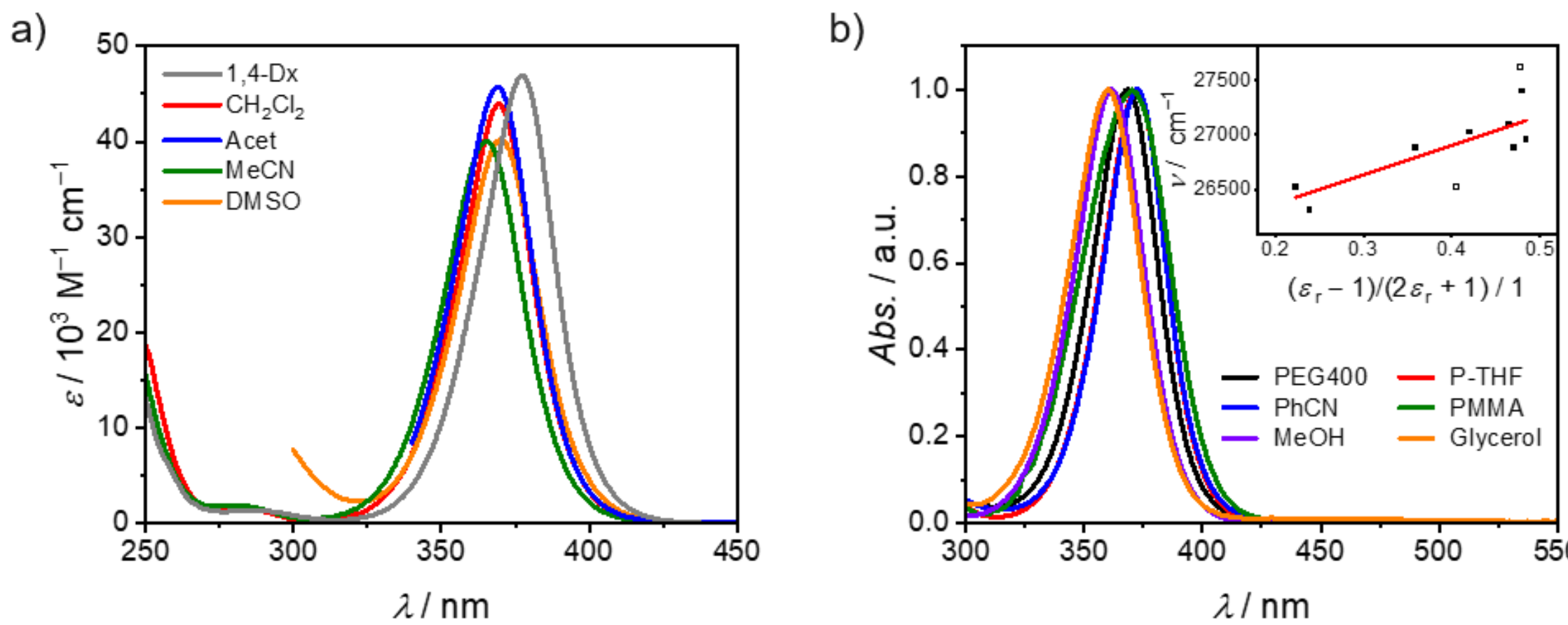

Fig. S9. UV-Vis absorption spectra of **PyBA** plotted either as (a) the molar extinction coefficient where available or as (b) the normalized absorbance, all measured at 293 K with concentrations of around 10^{-5} M. Notably, the sample used for the measurement of **PyBA** in 1,4-Dx was prepared under high dilution and addition of some drops of DMSO to dissolve the compound. Accordingly, the given extinction coefficient can only be regarded as a lower limit, and the sample was also not used for photoluminescence studies due to the presence of DMSO. Additionally, the solvent-dependent shift of the absorption band of **PyBA** was analysed within a model established by W. Liptay,[S26] describing the internal field of the solvent based on the Onsager theory,[S27] and is presented in the inset of (b). From the value of the ground state dipole moment (μ_g = 9.8 D) and an estimated Onsager radius (a = 5.05 Å, spherical approximation) derived from the crystal structure considering van-der-Waals radii, the analysis yields a dipole difference ($\Delta\mu$) of –3.0 D, which corresponds quite well to the values obtained by EOAM ($\Delta\mu$ = –3.7 D). Note that solvents like THF and MeOH which can form hydrogen bonds with **PyBA** were omitted for the analysis.

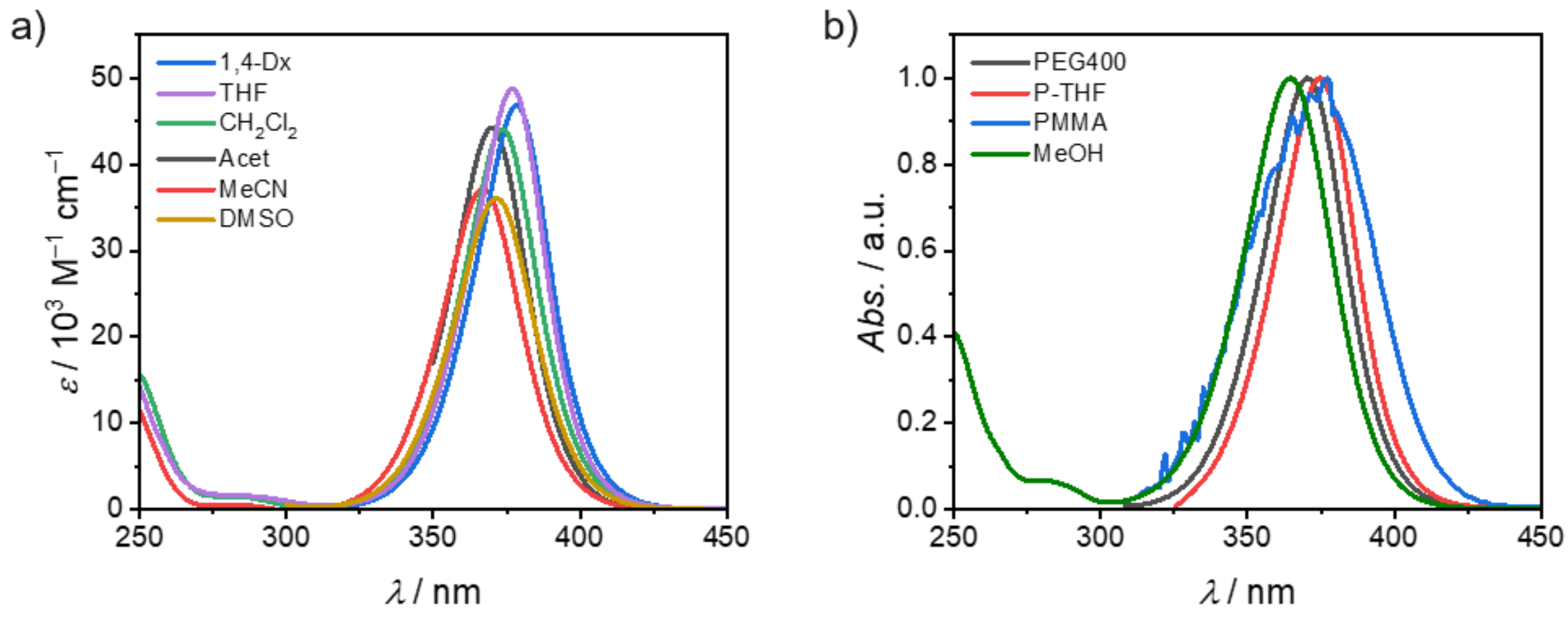

Fig. S10. UV-Vis absorption spectra of **PymBA** plotted either as (a) the molar extinction coefficient where available or as (b) the normalized absorbance, all measured at 293 K with concentrations of around 10^{-5} M.

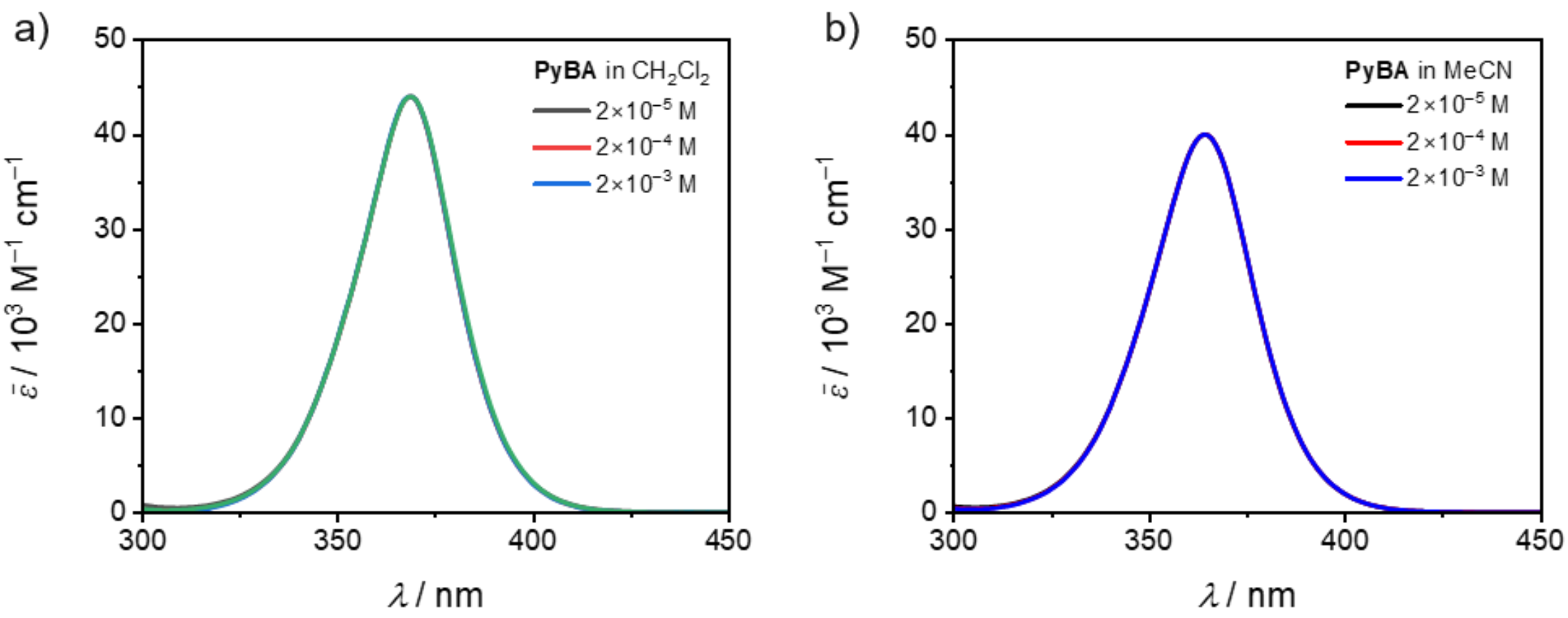

Fig. S11. Absorption spectra of **PyBA** in (a) CH_2Cl_2 and (b) MeCN at different concentrations, measured at 293 K. The selected concentrations were used throughout the manuscript for several measurements and are therefore depicted here to exclude the possibility of aggregation phenomena within the scope of this work. In accordance with literature.[S28]

6.2 Steady State Fluorescence Spectroscopy

Supplementary Photoluminescence Data of PyBA

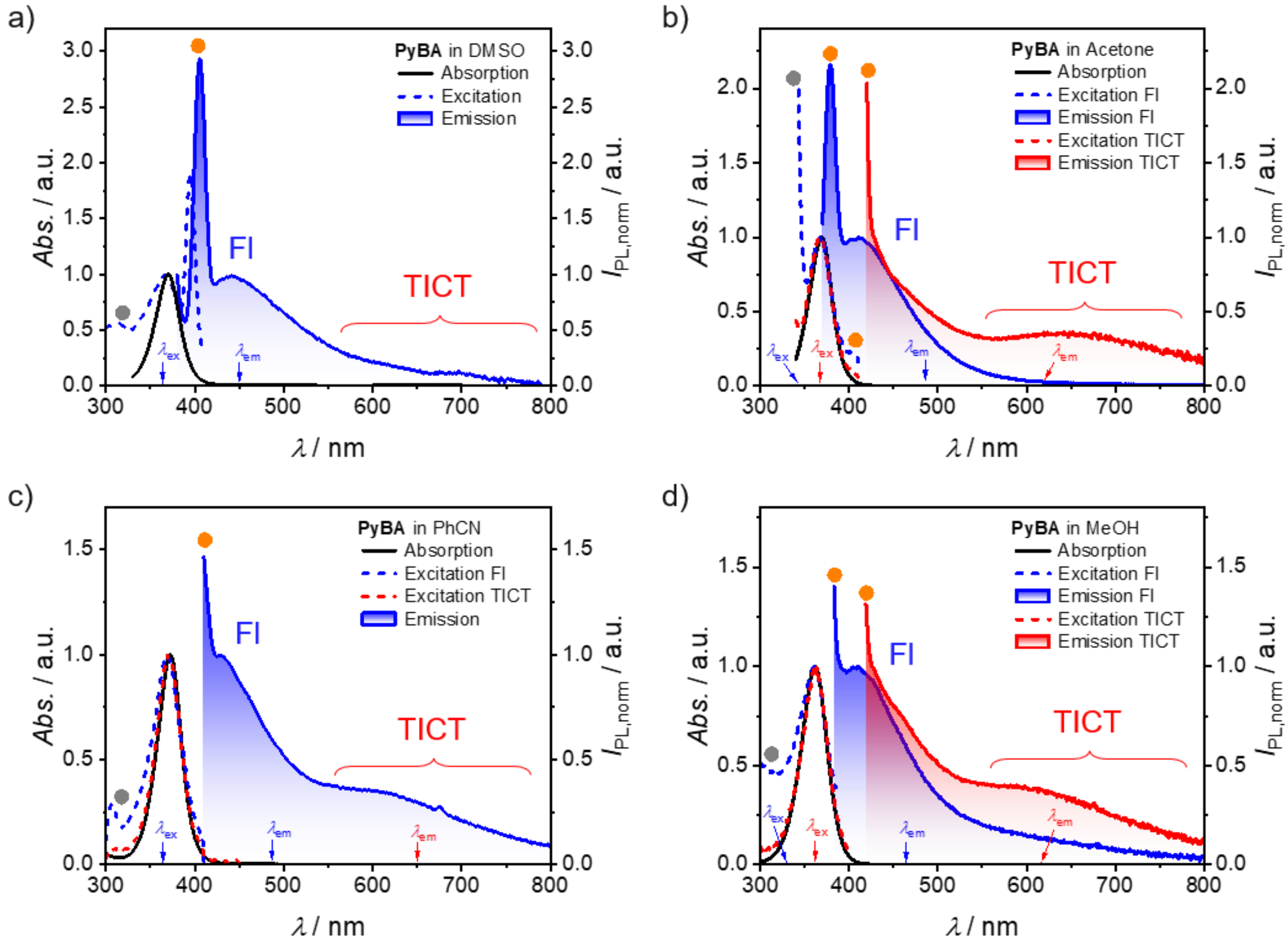

Fig. S12. Absorption (solid line), excitation (dashed line) and emission (solid, shaded line) spectra of **PyBA** in (a) DMSO, (b) acetone, (c) PhCN and (d) methanol (MeOH), measured at 293 K under ambient conditions. The orange circles indicate solvent artifacts arising from scattering phenomena, whilst the grey circles indicate signals arising due to autofluorescence of the solvent. The excitation and detection wavelengths are indicated with arrows, colorized according to the respective spectrum. In these four solvents, dual emission was observed, as depicted with TICT and Fl (fluorescence). Notably, in solvents where **PyBA** exhibits small Stokes shifts, two emission spectra at different excitation wavelengths are shown to visualize the whole emission spectrum with both the ^{1}LE maximum and the full TICT band (b and d).

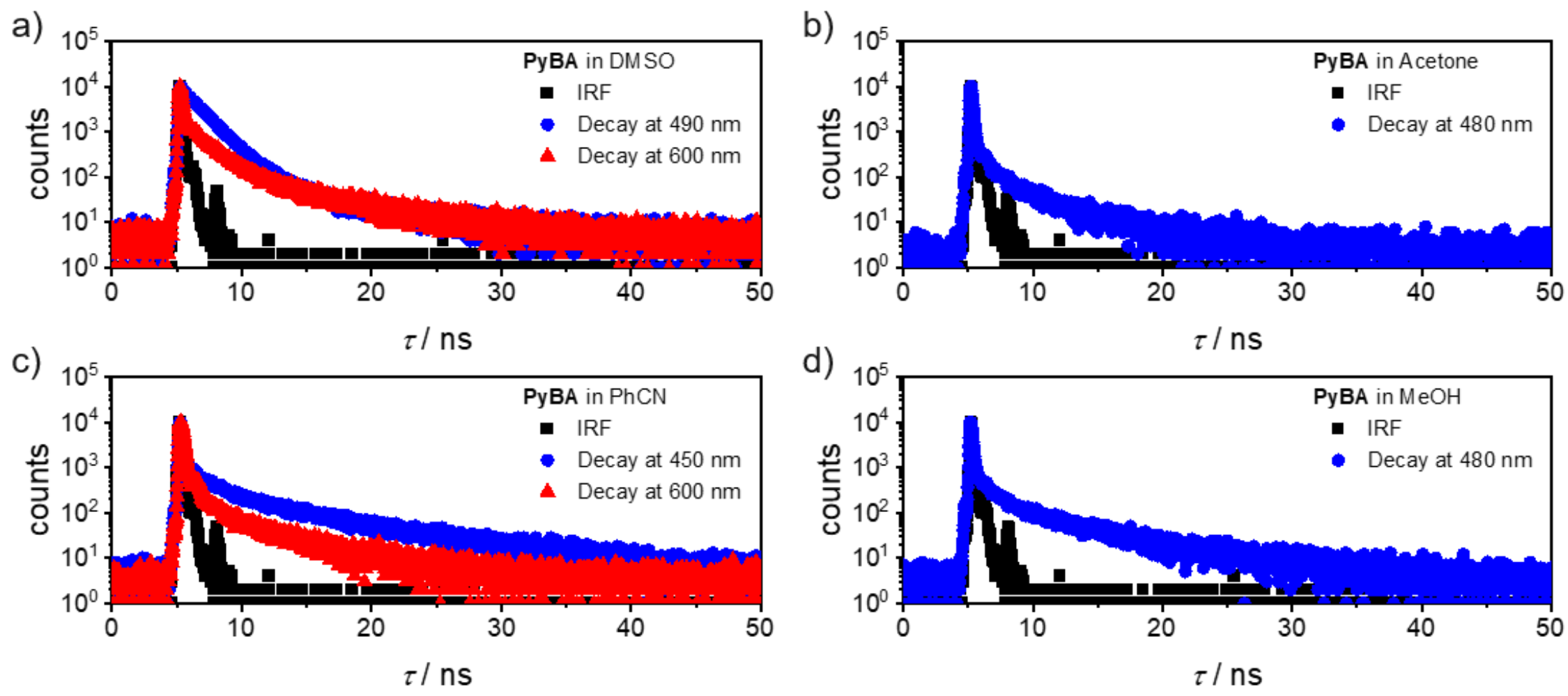

Fig. S13. Lifetime decay curves of **PyBA** in (a) DMSO, (b) acetone, (c) PhCN and (d) MeOH, collected via TCSPC at 293 K upon excitation at 378 nm with an EPL diode. Notably, the PL decays collected below 500 nm are partially affected by overlapping autofluorescence of the solvents. Thus, the extracted lifetime values represent only upper limits.

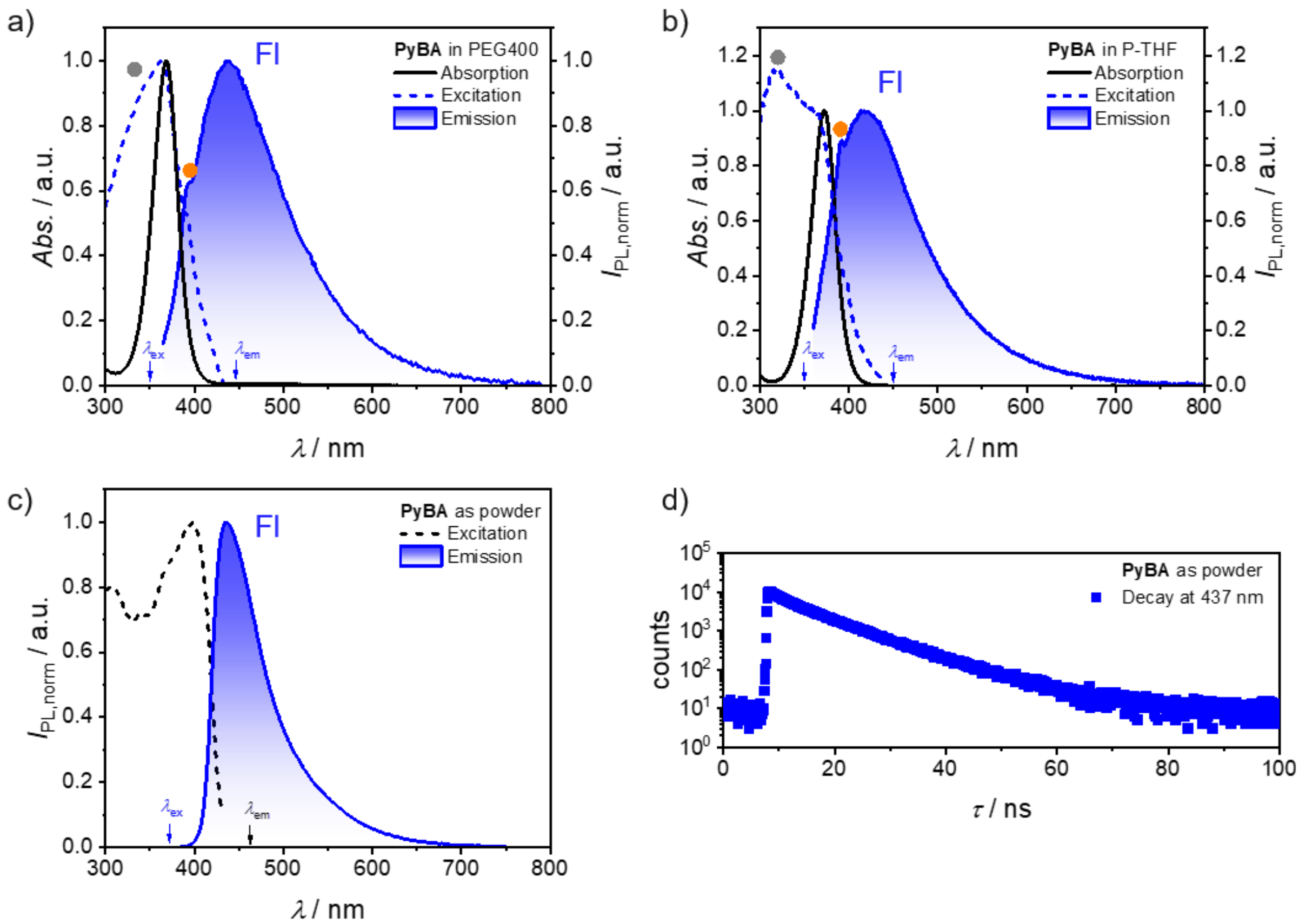

Fig. S14. Absorption (solid line), excitation (dashed line) and emission (solid, shaded line) spectra of **PyBA** in (a) poly(ethylene glycol) 400 (PEG400), (b) poly(tetrahydrofuan) (P-THF) and as (c) amorphous powder, measured at 293 K under ambient conditions. The orange circles indicate solvent artifacts arising from scattering phenomena, whilst the grey circles indicate signals arising due to autofluorescence of the solvent. The excitation and detection wavelengths are indicated with arrows, colorized according to the respective spectrum. In these three environments, only fluorescence (Fl) was observed due to the increased viscosity. (d) Lifetime decay of **PyBA** as amorphous powder, recorded via TCSPC upon excitation at 378 nm with an EPL diode. Notably, the photoluminescence data of the two polymeric liquids given in Table S7 is not complete (lifetime missing) due to the strongly overlapping signals with the solvent and possible influence of impurities/stabilizers of the solvent.

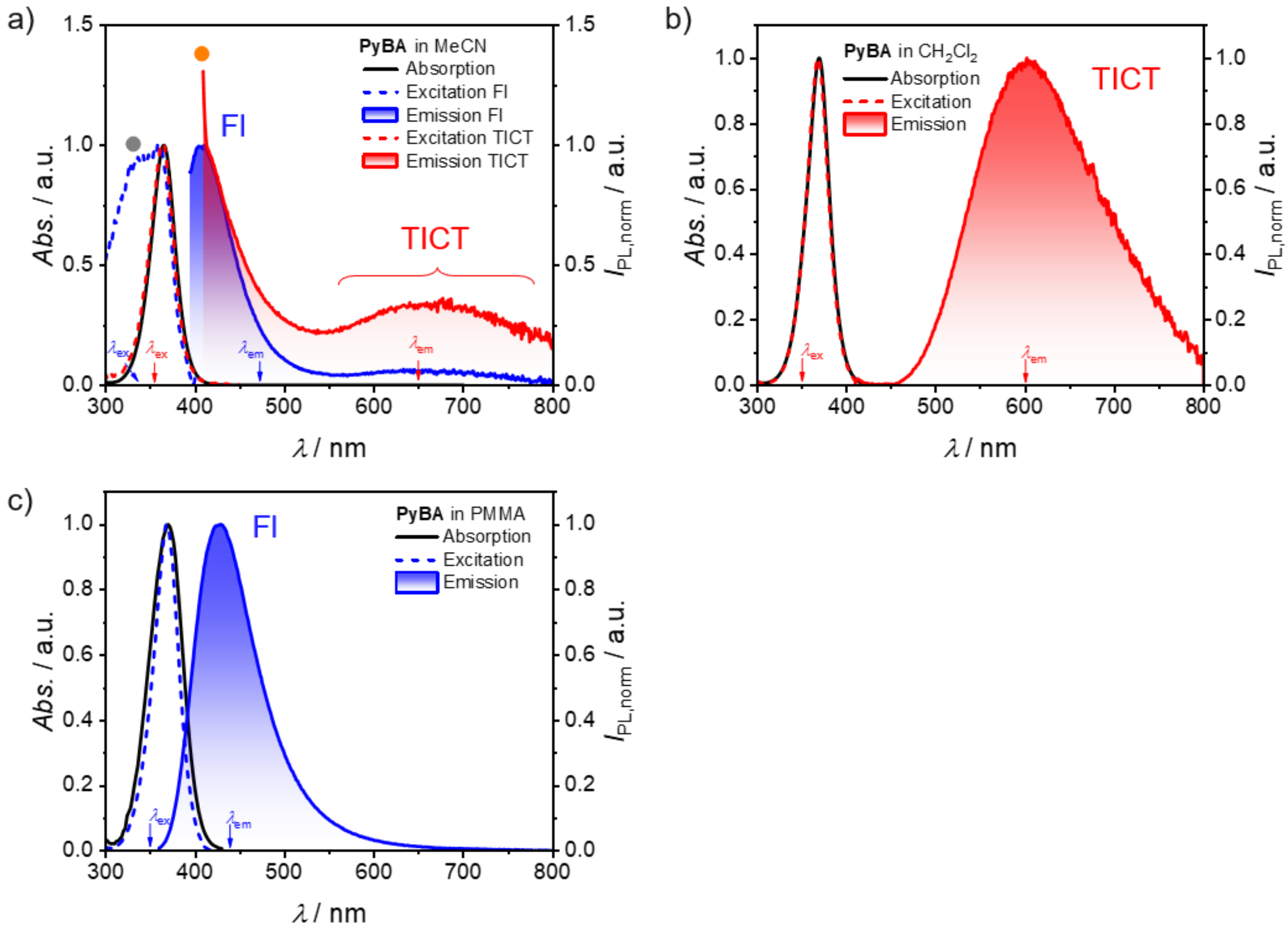

Fig. S15. Absorption (solid line), excitation (dashed line) and emission (solid, shaded line) spectra of **PyBA** in (a) MeCN, (b) CH_2Cl_2 and (c) PMMA thin film, measured at 293 K under ambient conditions. The orange circle indicates solvent artifacts arising from scattering phenomena, whilst the grey circle indicates signals arising due to autofluorescence of the solvent or contained stabilizers. The excitation and detection wavelengths are indicated with arrows, colorized according to the respective spectrum. Notably, for MeCN (a), where **PyBA** exhibits a small Stokes shift, two emission spectra at different excitation wavelengths are shown to visualize the whole emission spectrum with both the ^{1}LE maximum and the full TICT band.

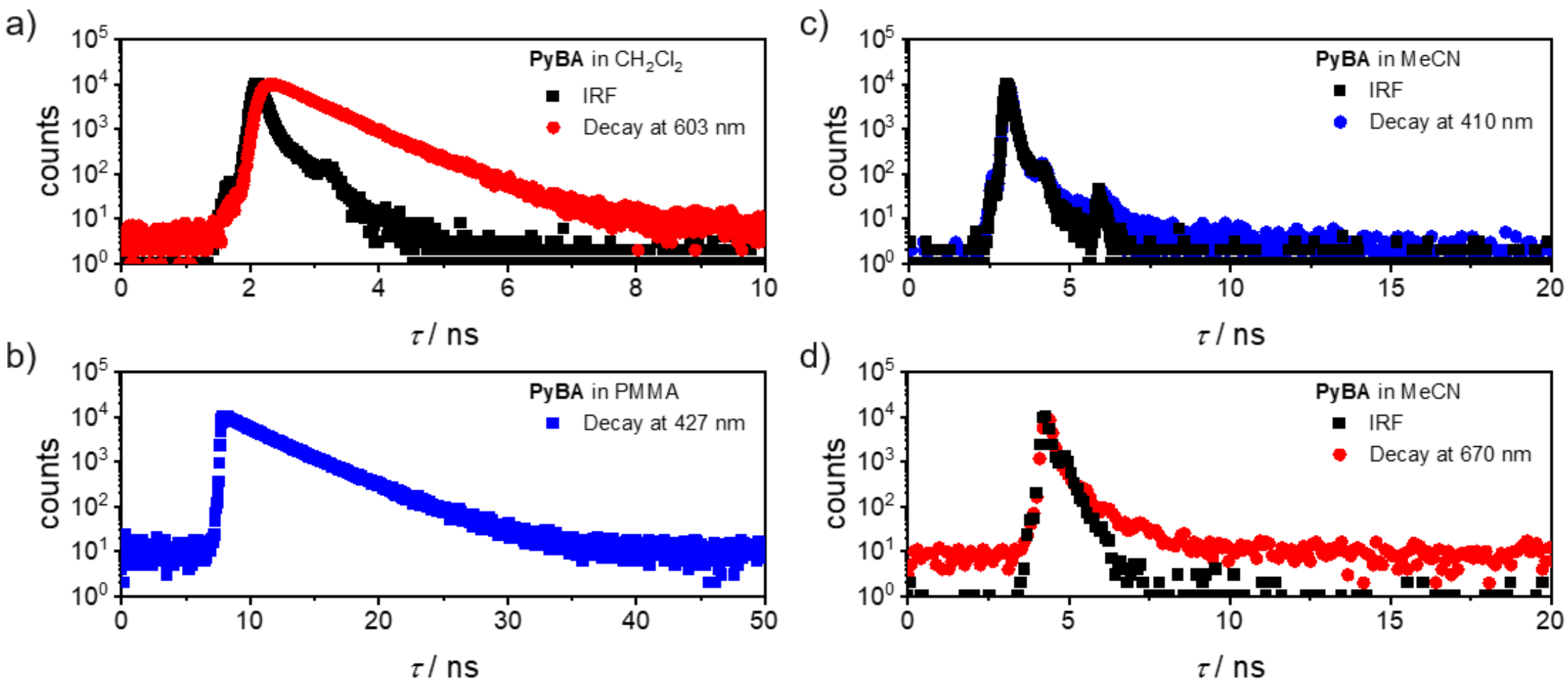

Fig. S16. Lifetime decay curves of **PyBA** in (a) CH_2Cl_2, (b) PMMA thin film and (c,d) MeCN, collected via TCSPC at 293 K upon excitation at 378 nm with an EPL diode.

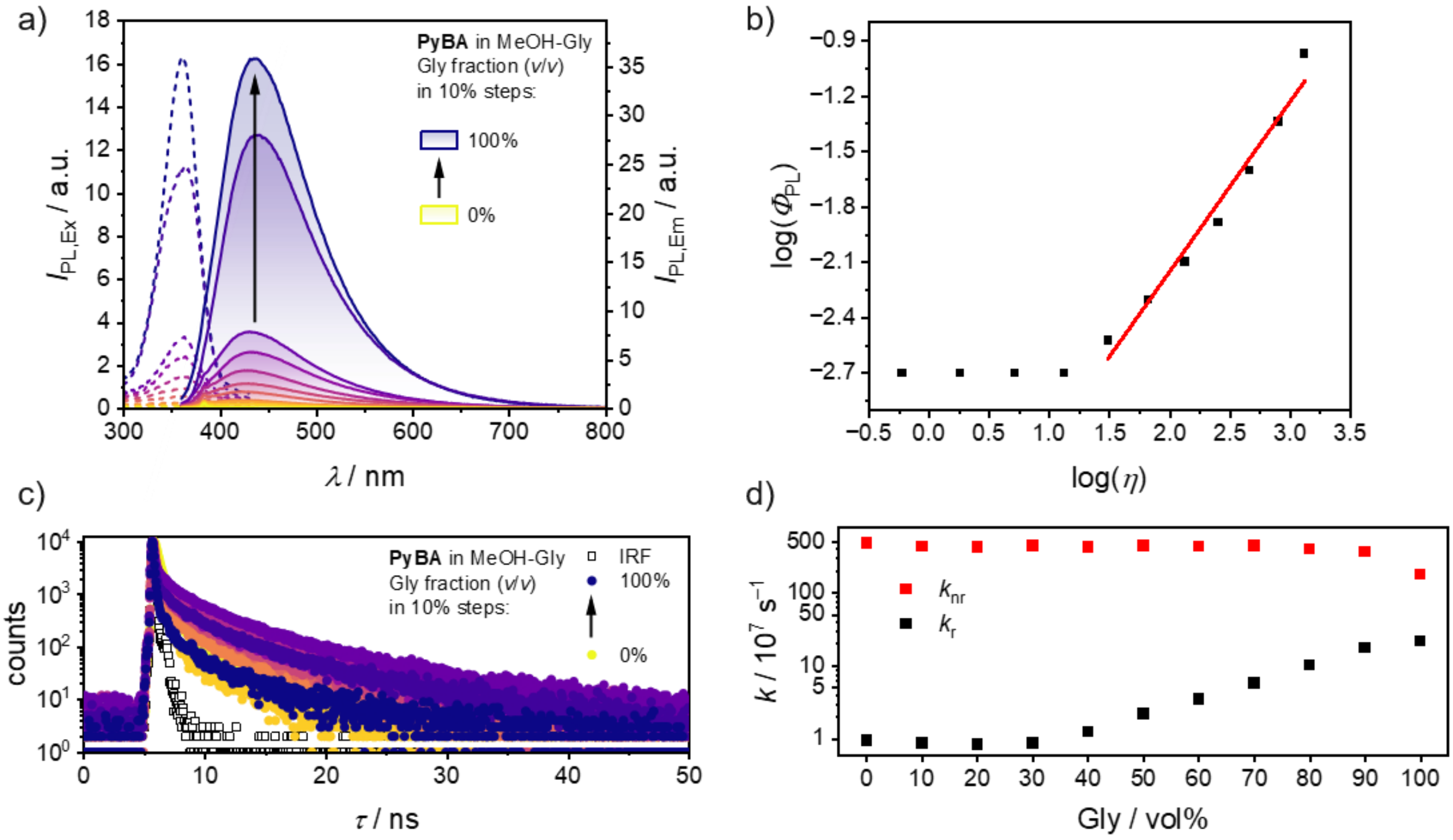

Fig. S17. a) Excitation (dashed lines) and emission (solid shaded lines) spectra of **PyBA** in binary mixtures of methanol and glycerol (Gly) at different ratios, measured at 293 K under ambient conditions. The emission and excitation spectra were collected upon excitation and detection at 345 nm and 500 nm, respectively. b) Förster-Hoffmann analysis of the viscosity-dependent Φ_{PL} of **PyBA** as a function of viscosity η in MeOH-Gly mixtures. The red line represents a least-squares linear regression of the viscosity-responsive regime (30.6 – 1317 mPa s), according to the Förster-Hoffmann relationship $\log(\Phi_{PL}) = C + x \log(\eta)$, where x is the dye-dependent constant and C is the temperature- and concentration-dependent constant. The fit yielded $x = 0.92 \pm 0.07$ and $C = -3.99 \pm 0.17$ with a fit quality R^2 of 0.971. The low-viscosity samples were excluded from the fit because their nearly constant, weak emission is affected by efficient TICT-state formation. Notably, the viscosity values of the mixtures depict only the trend of relatively increasing viscosity and are regarded as approximate regimes in this work, as the methanol-glycerol mixtures were prepared volumetrically and the exact viscosity was not measured afterwards. The values were extracted from Ref. S9. c) Corresponding Lifetime decay curves, collected via TCSPC at 293 K upon excitation at 378 nm with an EPL diode and detection at 440 nm. d) Accordingly calculated rate constants plotted against the glycerol fraction, demonstrating the plateau at low-viscosity mixtures and the gradual increase and decrease of the radiative and non-radiative rate constants in mid- to high-viscosity mixtures, respectively.

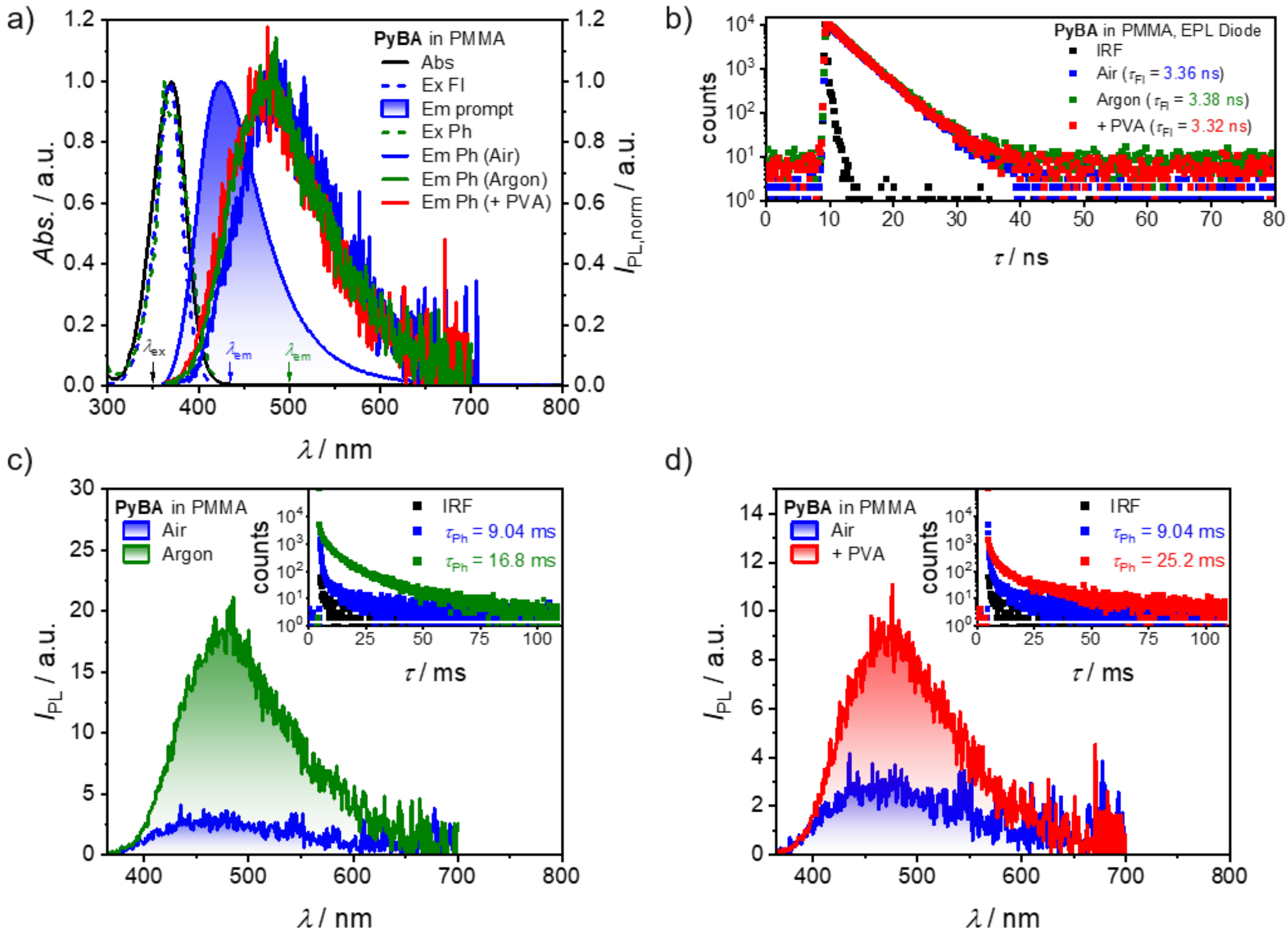

Fig. S18. a) Normalized absorption (black, Abs), excitation (dashed lines, Ex) and emission spectra (solid lines, Em) of **PyBA** in a PMMA thin film with prompt fluorescence (blue shaded) and phosphorescence, measured at 293 K under ambient conditions (blue), argon atmosphere (green) and upon coating with a PVA oxygen-barrier (red). Notably, only the prompt fluorescence spectrum under ambient conditions is shown, as the other two were identical. The phosphorescence spectra and their corresponding excitation spectrum were separately recorded with a microsecond flash lamp upon gating of the detector (delay 0.1 ms, width 9.9 ms). The excitation and detection wavelengths of each measurement are indicated with arrows, colorized according to the respective spectrum, whereas the excitation wavelength is given in black as it was used for all emission spectra. b) Lifetime decay curves corresponding to the fluorescence bands, measured upon excitation at 378 nm with an EPL diode via TCSPC. c,d) Phosphorescence spectra from (a) scaled along the relative intensity, measured with identical parameters. However, as the sample orientation and thickness differ throughout the measurements, the emission intensity does not serve as a quantitative comparison and both argon and PVA can only be compared to the measurement in air, respectively. For a better comparison, the corresponding lifetime decays, measured upon excitation at 370 nm with a microsecond flashlamp via MCS, are provided in the insets. Notably, only the longest components are given as the phosphorescence lifetimes and the decay measured for PMMA+PVA was best fitted with two components (2.1 ms and 25.2 ms), which is likely due to phase separation.

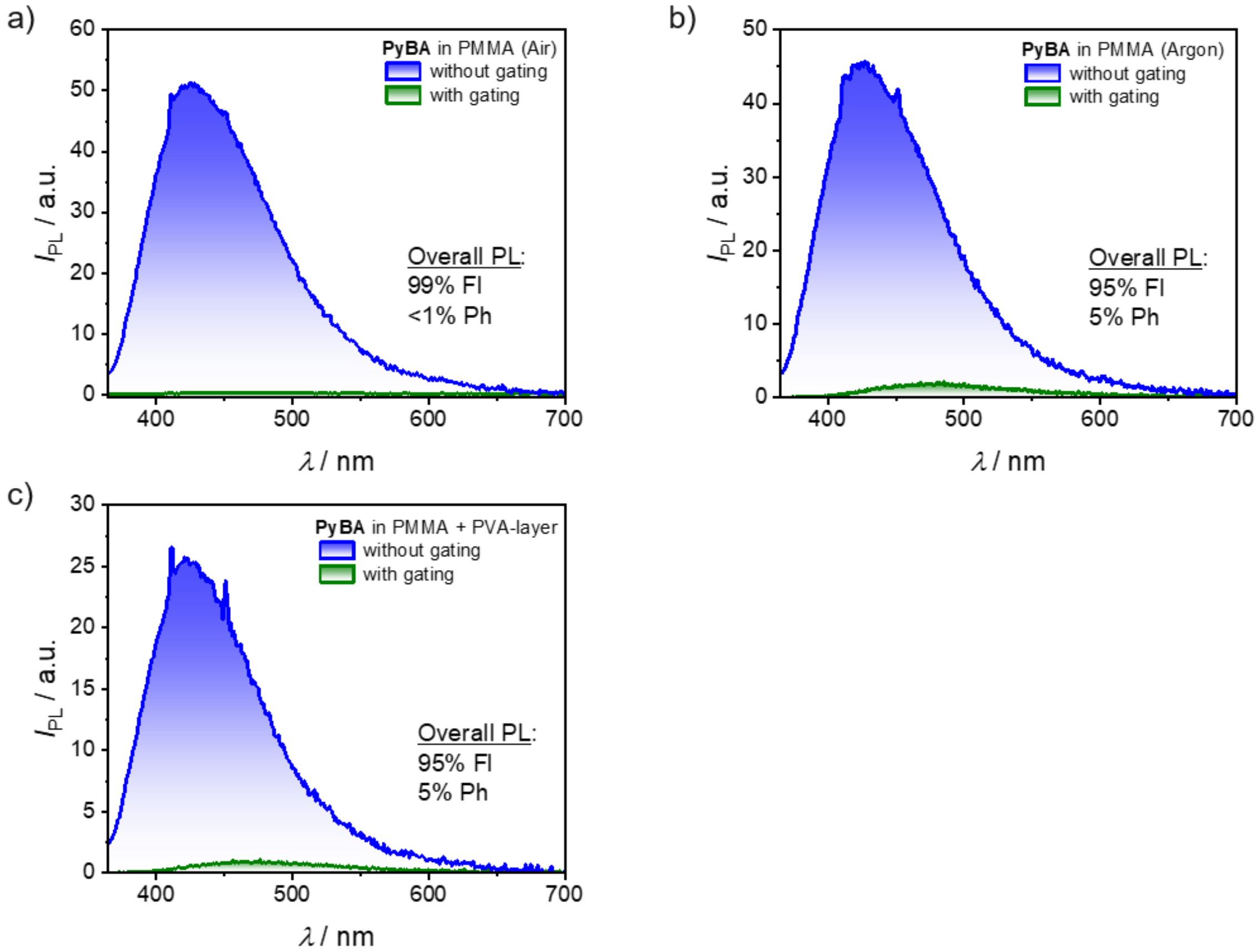

Fig. S19. Emission spectra of **PyBA** in PMMA, measured at 293 K with a microsecond flash lamp upon excitation at 350 nm under ambient conditions (a), argon atmosphere (b) and upon coating with a PVA oxygen-barrier (c). Comparison of the relative integral ratio of the spectra measured with (green) and without (blue) gating of the detector but otherwise identical parameters gives an approximate contribution of phosphorescence to the total photoluminescence. Notably, this method does not provide quantitative Φ_{PL} values but serves only as a rough comparison.

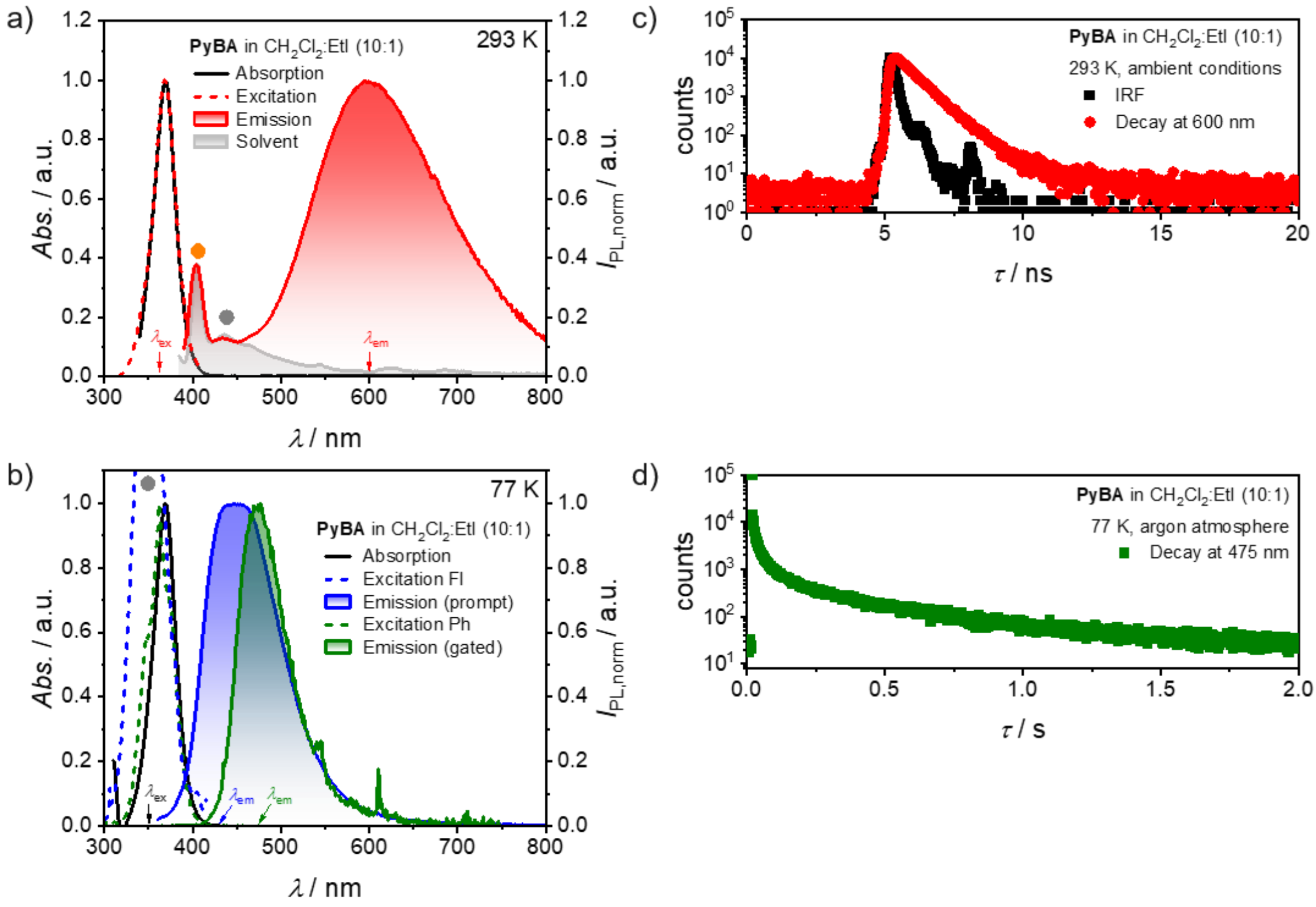

Fig. S20. Absorption (black), excitation (dotted) and emission (solid) spectra of **PyBA** in a mixture of CH_2Cl_2:EtI (10:1) at (a) 293 K and (b) 77 K with TICT emission in red, prompt fluorescence in blue and phosphorescence in green. The measurements performed at 293 K were recorded under ambient conditions, whilst the ones at 77 K were conducted under argon atmosphere. Notably, at 293 K, no phosphorescence occurred, even under argon atmosphere. The phosphorescence spectrum and its corresponding excitation spectrum were separately recorded with a microsecond flash lamp and upon gating of the detector (delay 0.1 ms, width 9.9 ms). The excitation and detection wavelengths of each measurement are indicated with arrows, colorized according to the respective spectrum, whereas the excitation wavelength in (b) is given in black as it was used for both emission spectra. The orange circles indicate solvent artifacts arising from scattering phenomena, whilst the grey circles indicate signals arising due to autofluorescence of the solvent. Furthermore, the emission spectrum of the pure solvent is given in (a) in grey to demonstrate the origin of the band at around 450 nm, confirming that TICT emission is the dominant emissive pathway of **PyBA** in CH_2Cl_2 (with or without ethyl iodide). Accordingly, this autofluorescence of the solvent is also the reason for the lacking shape complementarity of the blue excitation spectrum with the absorption spectrum in (b), where only the red edge is matching. Nonetheless, upon gating of the detector (green excitation spectrum), the spectra match again, indicating that the solvent alone does not exhibit phosphorescence. (c) Lifetime decay curve corresponding to the TICT band, measured at 293 K upon excitation at 378 nm with an EPL diode via TCSPC, providing similar lifetimes as in pure CH_2Cl_2. (d) Lifetime decay curve corresponding to the phosphorescence band, measured at 77 K upon excitation at 362 nm with a microsecond flashlamp via MCS and best fitted with a time constant of 318 ms for the longest component.

Table S6. Summary of experimentally determined energy values of **PyBA** from steady-state spectra, measured under an argon atmosphere, and respectively calculated singlet-triplet gaps. The values have been derived from the onset of each spectrum's tangent and then converting the wavelength into energy.

Environment	*T* / K	S_1–S_0 / eV	T_1–S_0 / eV	TICT–S_0 / eV	ΔE_{ST} / eV
CH_2Cl_2	293	-	-	2.56	-
	77	3.23	2.85	-	0.38
CH_2Cl_2:EtI (10:1)	293	-	-	2.58	-
	77	3.18	2.87	-	0.31
PMMA	293	3.29	3.10	-	0.19
	77	3.34	2.90	-	0.44

Table S7. Summary of optical properties of **PyBA**, measured at 293 K under ambient conditions and providing values for the absorption maximum (λ_{abs}), emission maximum (λ_{em}), Stokes shift ($\Delta\tilde{\nu}_{St}$), fluorescence lifetime (τ_{Fl}), amplitude averaged fluorescence lifetime ($\tau_{Fl,avg}$), photoluminescence quantum yield (Φ_{PL}), radiative rate (k_r) and non-radiative rate (k_{nr}). Notably, the reported rate constants relate to all radiative or non-radiative processes. The quantum yield values were measured absolutely and are not corrected for reabsorption, if below 1%. τ_{Fl} was usually best fitted using a bi- or tri-exponential fit function, thus giving two or three components, whose relative contribution to the overall intensity of the decay is given in brackets after each value. Accordingly, the amplitude averaged fluorescence lifetime $\tau_{Fl,avg}$ was calculated to provide rate constants. Furthermore, for PhCN and DMSO, the decay curves along both fluorescence and TICT pathways were recorded and thus two sets of lifetime parameters are provided (see Fig. S12).

Solvent	λ_{abs}/nm	λ_{em}/nm	$\Delta\tilde{\nu}_{St}$/cm^{-1}	$\tau_{Fl,1}$/ns	$\tau_{Fl,2}$/ns	$\tau_{Fl,3}$/ns	$\tau_{Fl,avg}$/ns	Φ_{PL}/%	k_r[a]/ 10^7 s^{-1}	k_{nr}[a]/ 10^7 s^{-1}
Acet[b]	369	410 (+642)[c]	2710 (+11520)[c]	< 0.20 (91%)	3.17 (9%)	-	< 0.20	0.3	> 1.47	> 489
PhCN[b]	372	430	3630	< 0.20 (64%)	1.46 (13%)	7.76 (23%)	< 0.21	0.4	> 1.92	> 479
		(+607)[c]	(+10410)[c]	< 0.20 (77%)	1.17 (12%)	5.77 (11%)	< 0.22		> 1.79	> 445
DMSO[b]	371	442	4330	1.38[d] (96%)	5.78[d] (4%)	-	1.42[d]	0.3	0.21[d]	70.1[d]
		(+696)[c]	(+12590)[c]	< 0.20 (72%)	1.55 (20%)	7.04 (8%)	< 0.20		> 1.47	> 488
MeOH[b]	362	410 (+594)[c]	3230 (+10790)[c]	< 0.20 (88%)	3.75 (12%)	-	< 0.21	0.2	> 0.96	> 478
Glycerol	360	435	4790	0.45 (88%)	3.34 (12%)	-	0.50	10.7	21.5	179
CH_2Cl_2:EtI (10:1)	369	598	10380	0.58 (99%)	3.18 (1%)	-	0.58	n.d.[e]	n.d.[e]	n.d.[e]
PEG400	368	437	4290	n.d.[e]	-	-	-	1.0[d]	n.d.[e]	n.d.[e]
P-THF	372	417	2970	n.d.[e]	-	-	-	0.6[d]	n.d.[e]	n.d.[e]
Powder	398[f]	435	-	3.22 (17%)	8.95 (83%)	-	6.85	24	3.50	11.1

[a] Determined according to $k_r = \Phi_{PL}\ \tau_{avg}^{-1}$ and $k_{nr} = \tau_{avg}^{-1} - k_r$. [b] The denoted lifetime values in this solvent can only be recognized as upper limits as such short-lived components are below the instrument response time of the TCSPC setup. Accordingly, thereby calculated rate constants reflect only lower limits. [c] Values corresponding to the TICT emission band. Note that the Stokes shift is formally not correct to calculate with the TICT maximum, it is just provided for comparison with CH_2Cl_2. [d] The real value is presumed to be lower (rate constants higher) due to strong overlapping autofluorescence of the solvent and its impurities/stabilizers in the excitation and detection region. [e] n.d. = not determined, often due to insufficient emission of the chromophore in this environment or overlapping signals from autofluorescence of the solvent. [f] This value corresponds to the maximum of the excitation spectrum, not the absorption spectrum.

Table S8. Summary of optical properties of **PyBA**, measured at 293 K under ambient conditions in binary mixtures of methanol and glycerol (Gly) at different ratios, providing values for the absorption maximum (λ_{abs}), emission maximum (λ_{em}), amplitude averaged fluorescence lifetime ($\tau_{Fl,avg}$), photoluminescence quantum yield (Φ_{PL}), radiative rate (k_r) and non-radiative rate (k_{nr}). Notably, the reported rate constants relate to all radiative or non-radiative processes. The quantum yield values were measured absolutely.

Gly fraction (v/v)	η^a / mPa s	λ_{abs} / nm	λ_{em} / nm	$\tau_{Fl,avg}{}^c$ / ns	Φ_{PL} / %	$k_r{}^{b,c}$ / 10^7 s^{-1}	$k_{nr}{}^{b,c}$ / 10^7 s^{-1}
0	0.60	362	410	0.21	0.2	0.96	478
10	1.80	360	418	0.23	0.2	0.87	432
20	5.20	360	419	0.24	0.2	0.84	420
30	13.2	360	421	0.23	0.2	0.87	436
40	30.6	360	422	0.23	0.3	1.26	418
50	66.0	360	424	0.23	0.5	2.20	438
60	133.2	360	428	0.23	0.8	3.47	431
70	253.5	360	430	0.23	1.3	5.75	437
80	458.6	360	430	0.25	2.5	10.1	393
90	793.0	360	436	0.26	4.6	17.4	361
100	1317.0	360	435	0.50	10.7	21.5	179

[a] Viscosity values extracted from Ref. S9, measured at 20 °C. Notably, the values of the mixtures depict only the trend of relatively increasing viscosity and are regarded as approximate regimes in this work, as the methanol-glycerol mixtures were prepared volumetrically and the exact viscosity was not measured. [b] Determined according to $k_r = \Phi_{PL}\ \tau_{avg}^{-1}$ and $k_{nr} = \tau_{avg}^{-1} - k_r$. [c] The denoted lifetime values can only be recognized as upper limits as the short-lived components (τ < 0.2 ns) are below the instrument response time of the TCSPC setup. Accordingly, thereby calculated rate constants reflect only lower limits.

Photostability Measurements

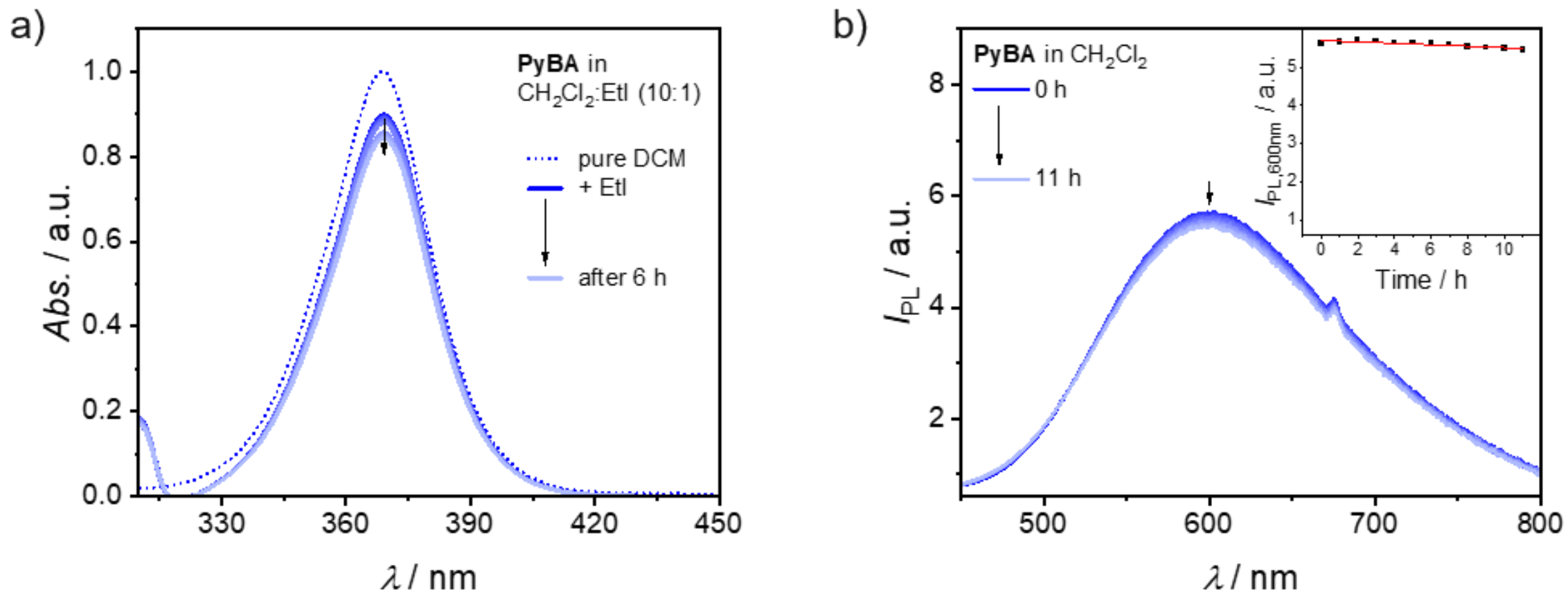

Fig. S21. a) Time-dependent UV-Vis absorption spectra of **PyBA** in CH_2Cl_2:EtI (10:1) to investigate the dyes' stability in this solvent mixture after continuous irradiation with a deuterium lamp at 370 nm over the course of 6 h. No new bands, indicative of adduct formation or decomposition, were forming and the absorbance was almost consistent over time, decreasing only slightly as indicated with the black arrows. For reference, the absorption spectrum of **PyBA** in pure CH_2Cl_2 before the addition of ethyl iodide is provided as a dotted line. b) Photodegradation assay of **PyBA** in CH_2Cl_2 solution (c ≈ 1 µM) over the course of 11 h whilst the sample was continuously illuminated at 370 nm (absorption maximum) by a 450 W Xenon lamp (bandwidth = 8 nm) under ambient conditions. During the experiment, an emission spectrum was recorded every hour and the intensity at the emission maximum is plotted over time (inset).

Temperature-Dependent Emission Spectra of PyBA

For modulation of the excited state dynamics of **PyBA** by inhibition of TICT formation, temperature-dependent emission spectra in a CH_2Cl_2 solution have been recorded. Upon cooling the sample, the emission band associated with fluorescence from the ^{1}LE state increases in intensity. However, as the temperature was kept above the melting point of CH_2Cl_2 to remain in the condensed phase, TICT emission was not inhibited completely. Thus, for a full inhibition of TICT formation, the sample either needs to be frozen completely or a more viscous environment like a PMMA matrix has to be chosen. The quantum yield at lower temperatures was determined by choosing an excitation wavelength that showed no changes of the optical density upon cooling and comparing the integral of the resulting emission spectra at different temperatures, whilst keeping all other measurement parameters identical.

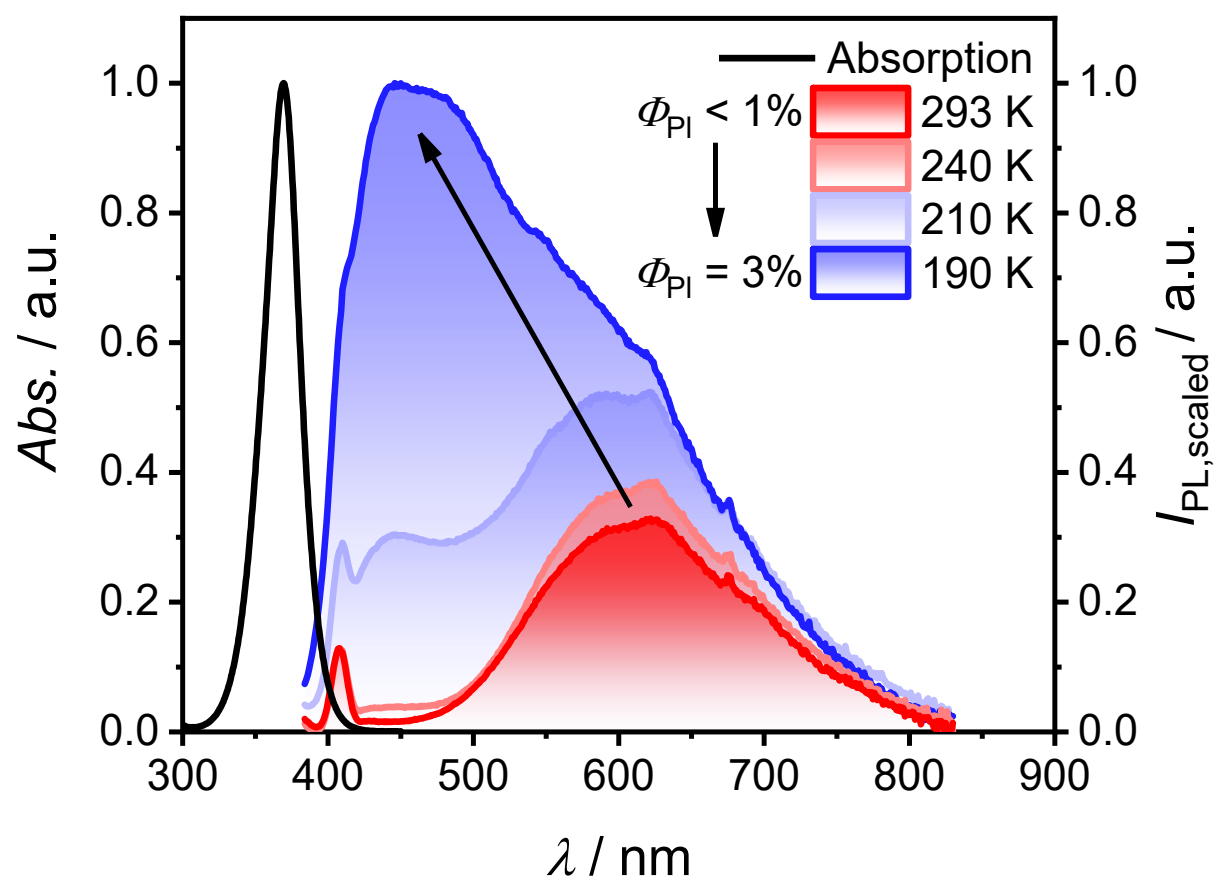

Fig. S22. Absorption (black) and temperature-dependent emission spectra of **PyBA** in CH_2Cl_2 upon excitation at 362 nm and cooling from 293 K to 190 K. The emission spectra have been plotted respective to the relative integral ratio and thus give rise to the increase in photoluminescence quantum yield by inducing ^{1}LE emission, whilst still partially observing TICT emission.

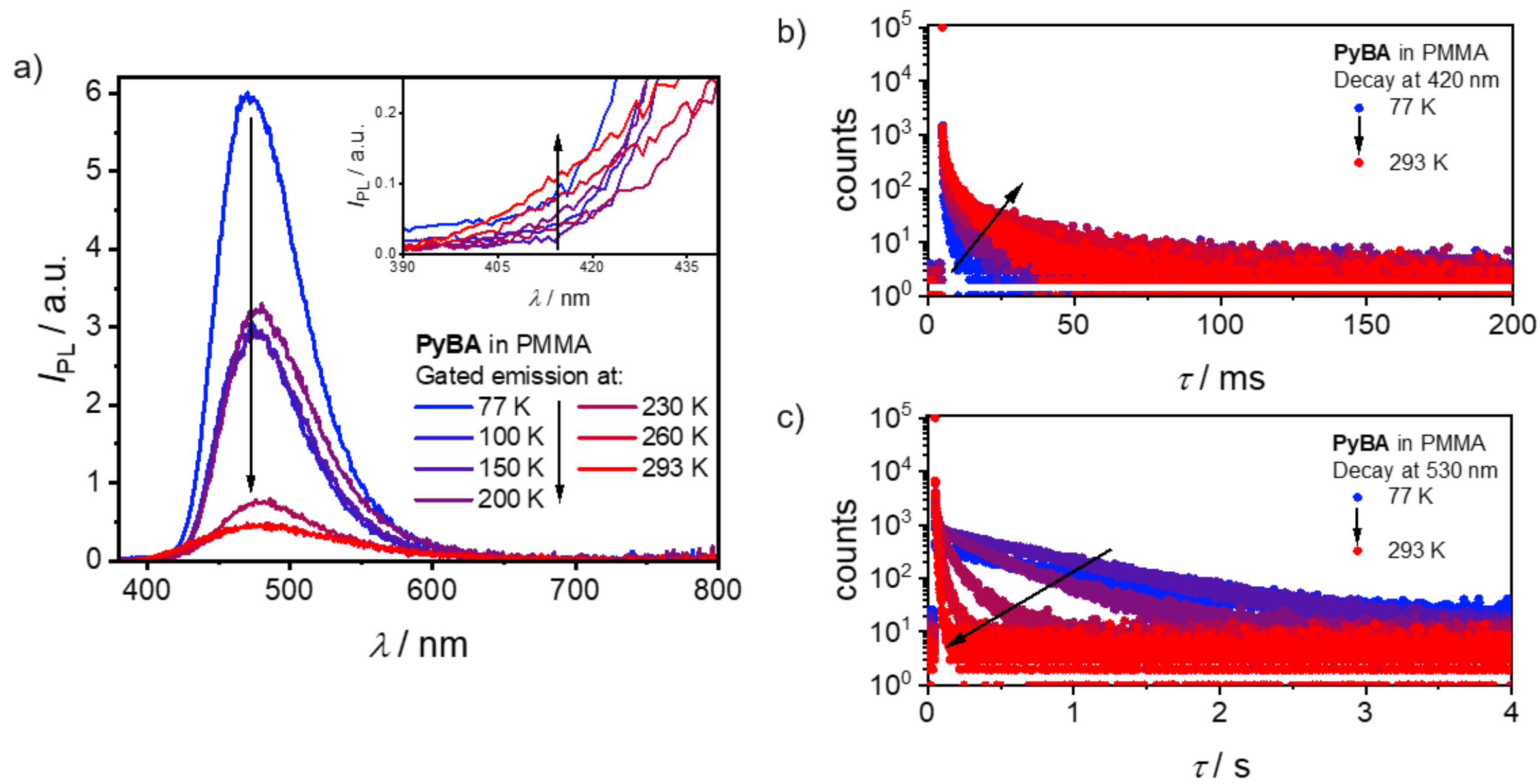

Fig. S23. a) Temperature-dependent emission spectra of **PyBA** in a PMMA thin film under argon atmosphere upon gating of the detector (delay 0.1 ms, width 9.9 ms), excitation at 350 nm with the microsecond flash lamp and heating from 77 K to 293 K (blue to red). Whilst the phosphorescence band is decreasing with increasing temperature, the spectrum is also

broadening and exhibits a slight shoulder at the blue edge of the spectrum, indicative of delayed fluorescence. Additionally, at each temperature step, lifetime decay curves were collected at (b) 420 nm and (c) 530 nm upon excitation at 370 nm with the microsecond flash lamp via MCS, thus providing proof for the existence of thermally activated delayed fluorescence (TADF) with the extension of decays in (b) and phosphorescence with the shortening of decays in (c) upon increasing temperatures.

Comparison of PymBA with PyBA

For comparison of **PymBA** with **PyBA** and verification of their similar photophysical pathways, measurements in CH_2Cl_2 solution and PMMA thin film have been carried out, which is depicted below in Fig. **S24**. Accordingly, TICT emission in the dynamic solution and fluorescence from ^{1}LE in the rigid matrix are observed. Additionally, photoluminescence studies in 1,4-Dx and THF were conducted to investigate the excited state dynamics of **PymBA** in low polarity solvents. In both solvents, dual emission was observed with both fluorescence and TICT emission arising. The same result can presumably also be expected for **PyBA**, considering their matching properties and behaviour in the other environments.

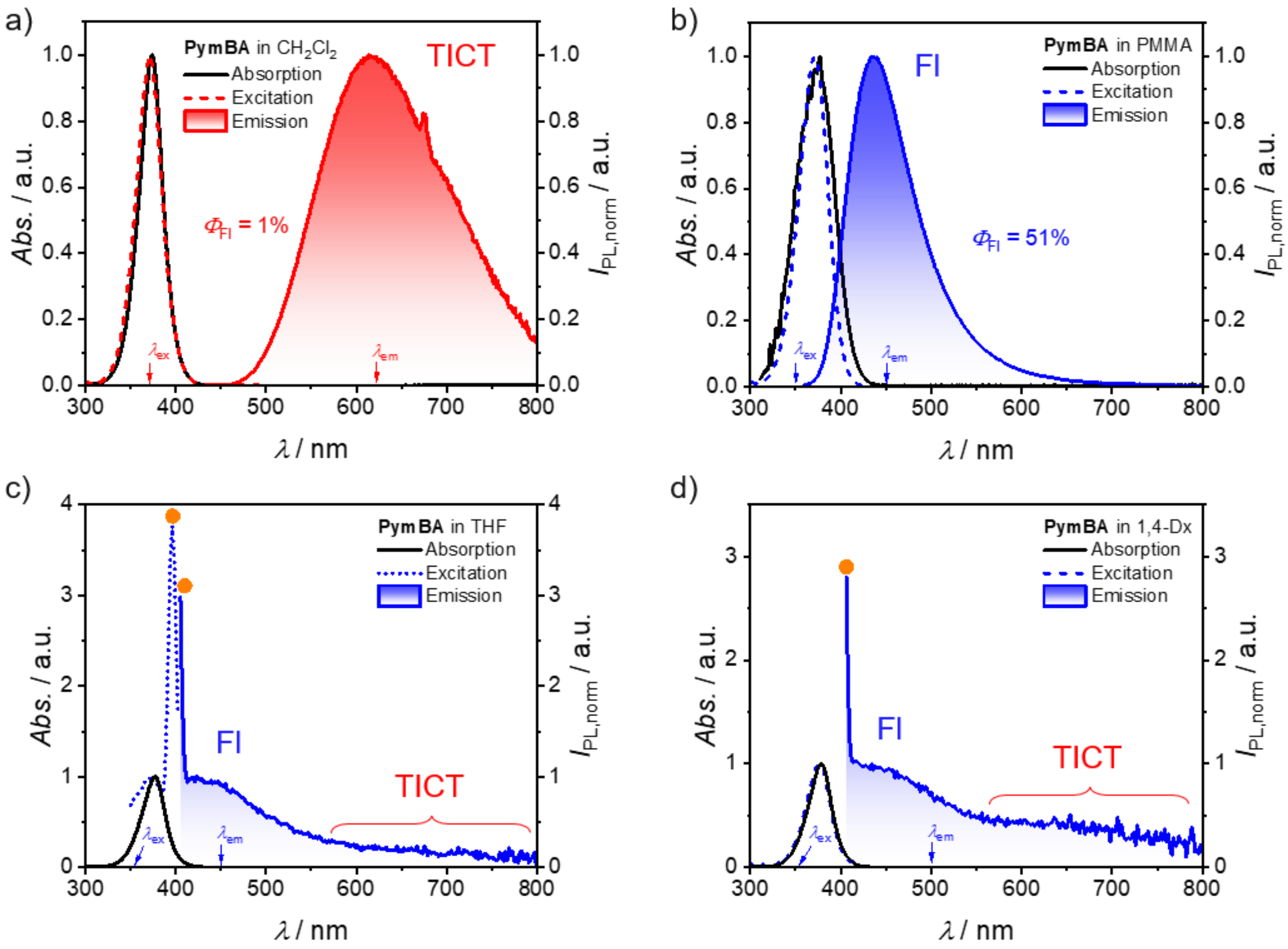

Fig. S24. Absorption (black line), excitation (dashed line) and emission spectra (solid shaded line) of **PymBA** in (a) CH_2Cl_2, (b) PMMA matrix, (c) THF and (d) 1,4-dioxane, measured at 293 K. The excitation and detection wavelengths are indicated with arrows. Fluorescence is depicted in blue, whereas TICT emission is indicated in red. The orange circles indicate solvent artifacts arising from scattering phenomena.

6.3 Time-resolved fs-Transient Absorption

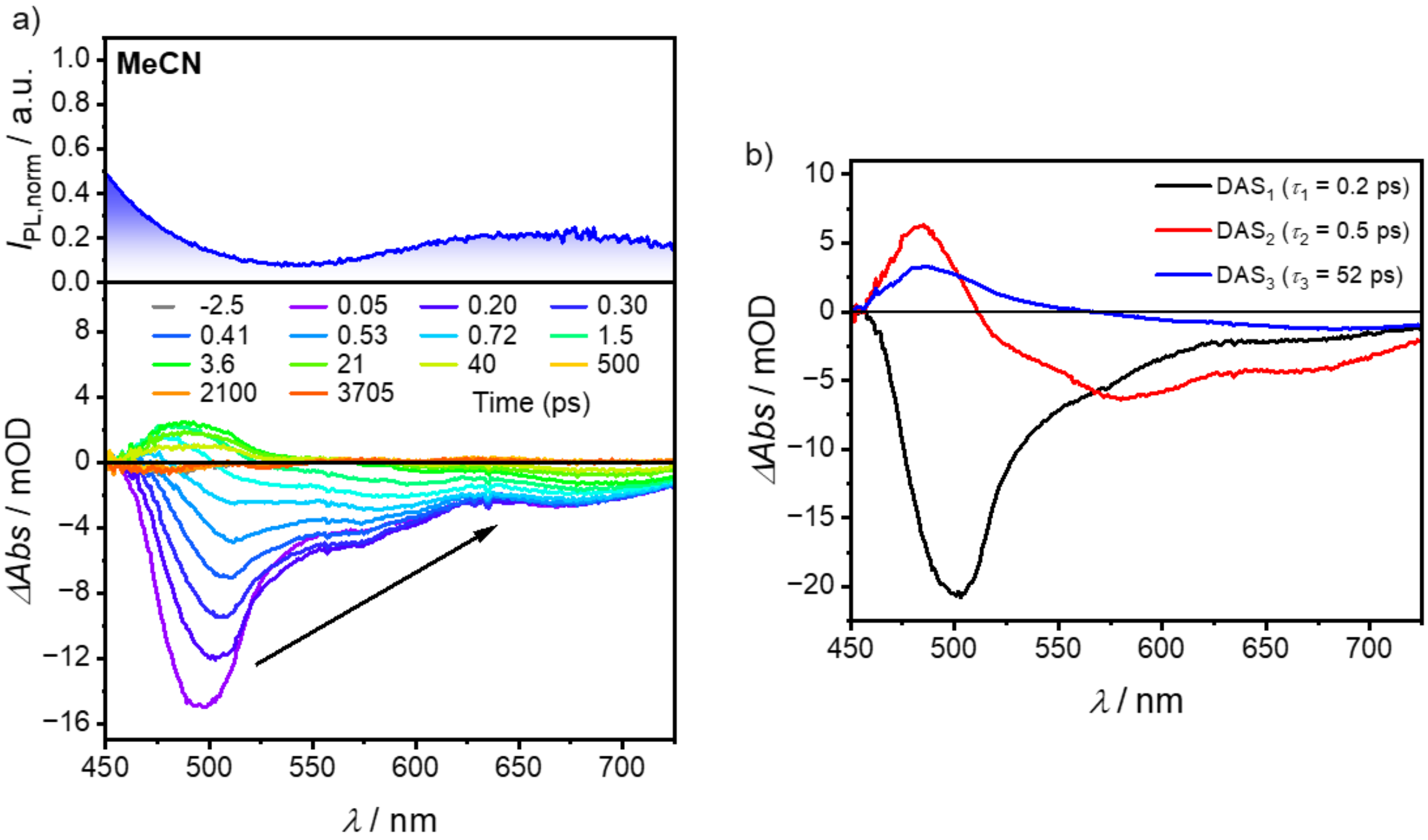

Fig. S25. a) Steady state emission spectrum upon excitation at 350 nm (top panel), fs-TA spectra upon excitation at 370 nm with respective time delay steps (bottom panel) and b) decay-associated spectra (DAS) with respective time constants of **PyBA** in MeCN, recorded at 293 K under ambient conditions. The arrow indicates the progression of the stimulated emission band. The concentration of the sample was 0.5 mM.

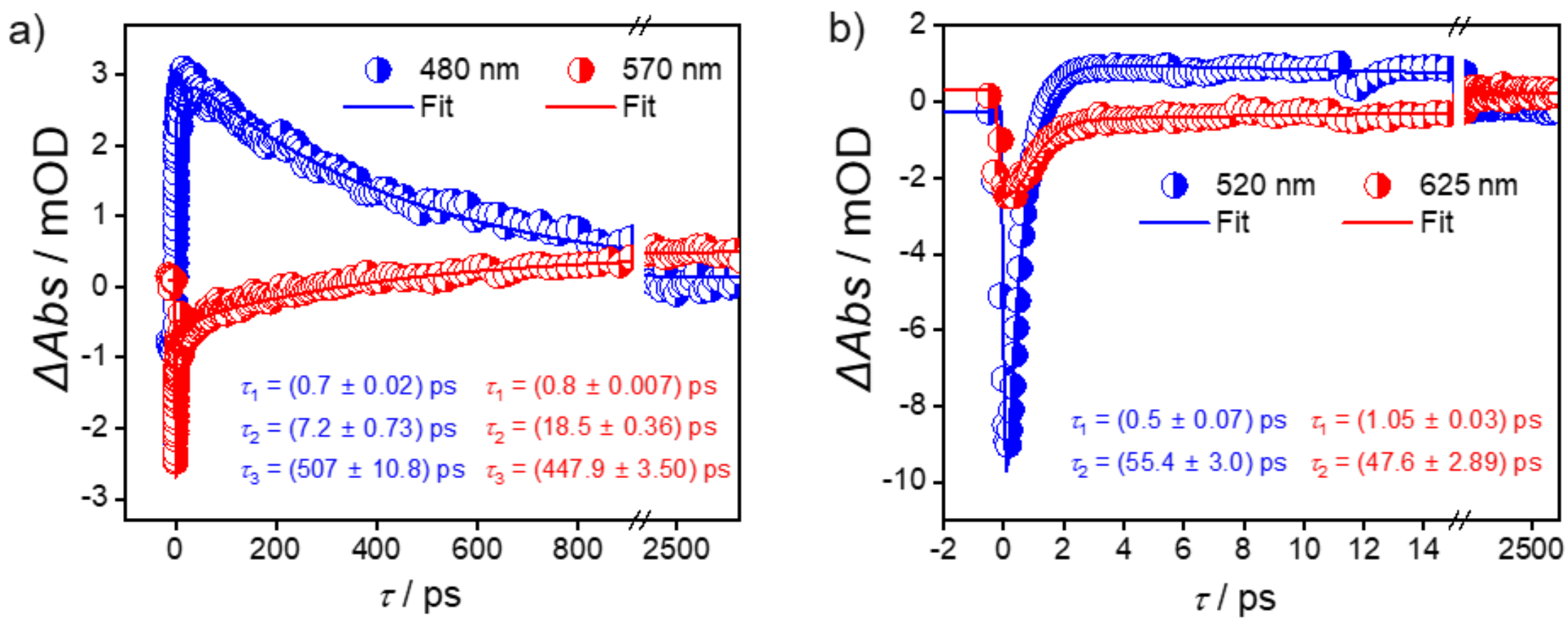

Fig. S26. Single wavelength kinetics of **PyBA** at various probe wavelengths of fs-TA in (a) CH_2Cl_2 and (b) MeCN. The corresponding time constants are given.

6.4 Time-resolved fs-Fluorescence Up-Conversion

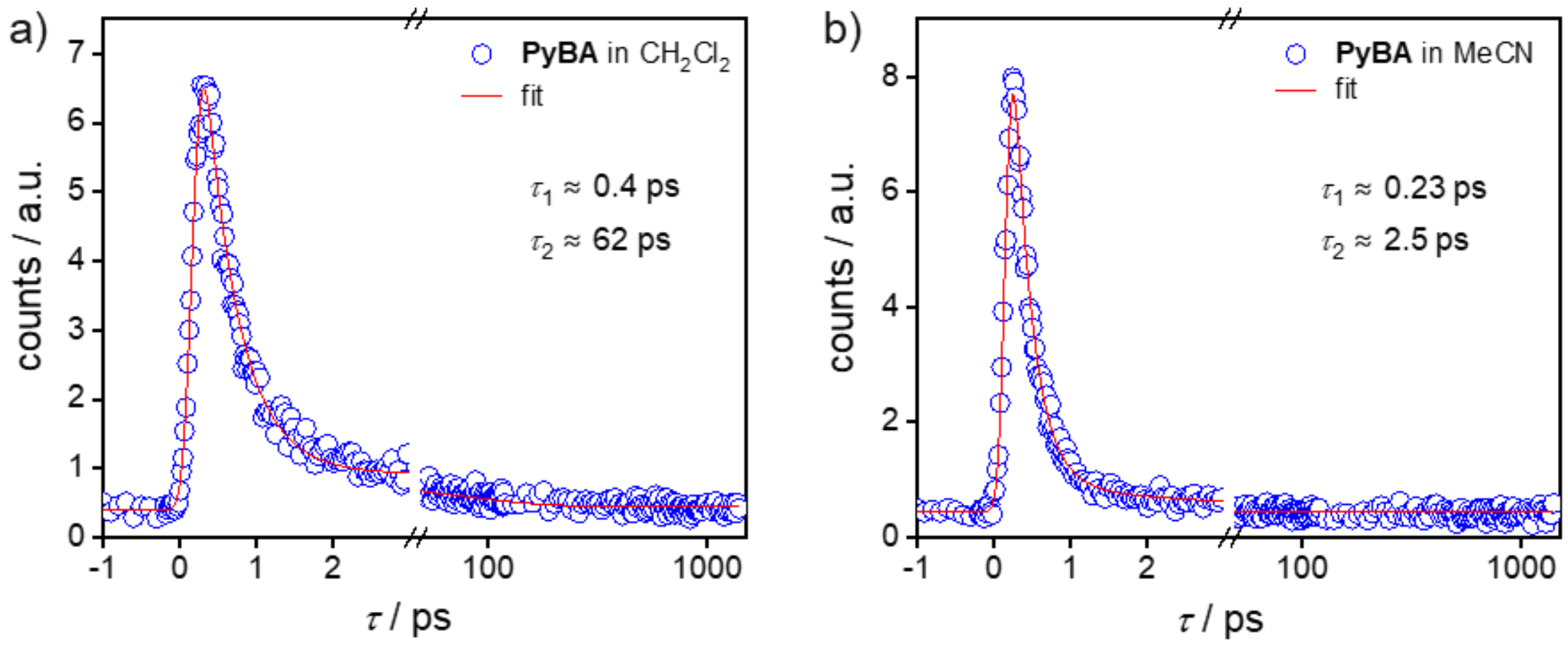

Fig. S27. fs-Fluorescence up-conversion plots of **PyBA** in (a) CH_2Cl_2 and (b) MeCN upon excitation at 400 nm and probing at 550 nm. The corresponding time constants are given. Notably, 95% of the excited state population decayed within sub-picosecond timescales, which can be attributed to the ultrafast relaxation of the ICT state. The concentration of the sample was 2.0 mM.

7 Quantum-Chemical Calculations

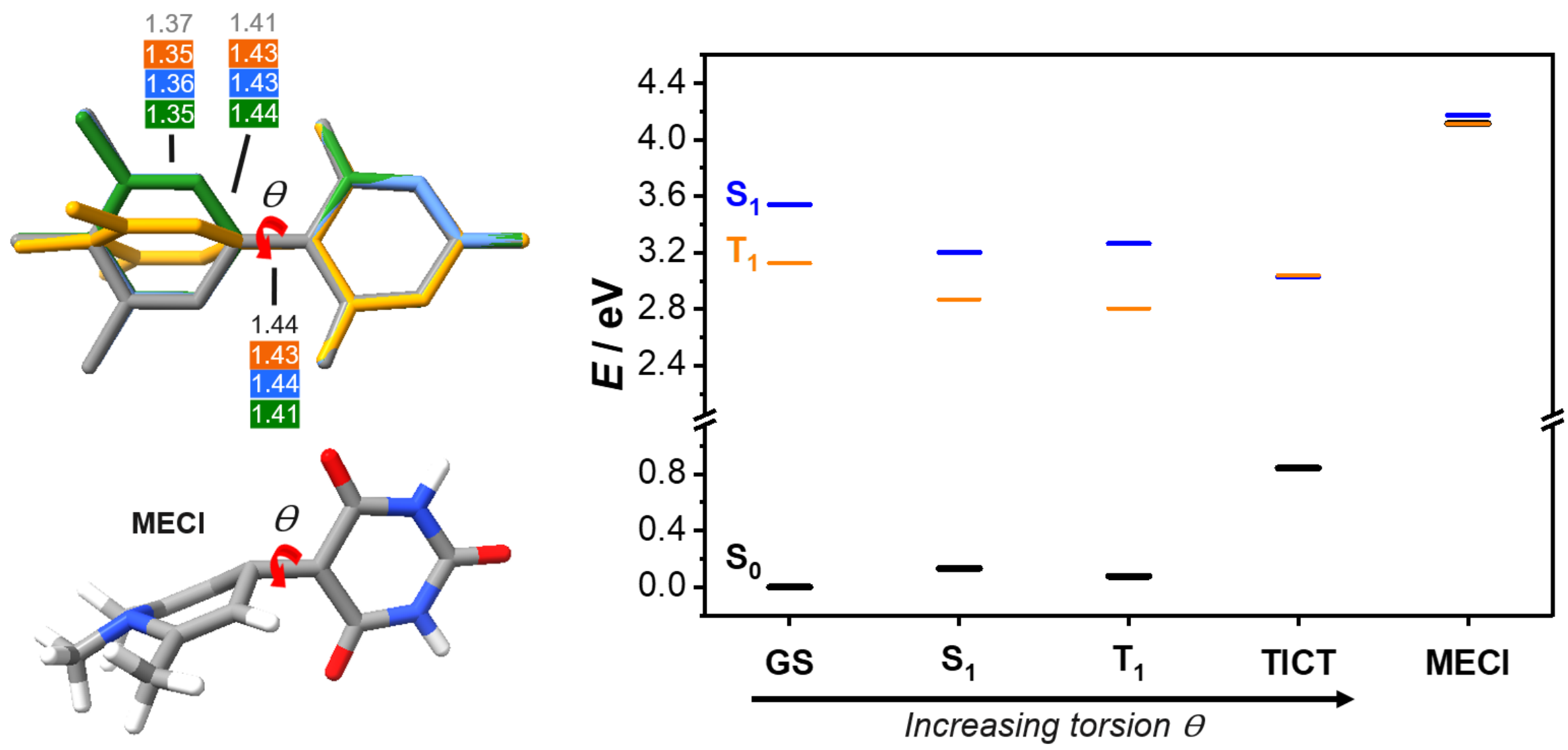

Fig. S28. Optimized geometries of **PyBA** in the ground state (GS, gray), approximated constrained S_1 excited state (blue), T_1 triplet excited state (green), TICT singlet excited state (orange), and at a S_0/S_1 minimum energy conical intersection (MECI) point (coloured by element). The structures were optimized at the (TD-)ωB97X-D3/def2-TZVP level of theory within the solvent continuum model in CH_2Cl_2, except the MECI geometry, which was optimized with mixed-reference spin-flip TD-DFT at the BH&HLYP/6-31+G* level of theory in the gas phase. The distances of the selected carbon-carbon bonds are marked in the respective colour of each structure on the left and given in Å. The excited state energy levels were further refined with SA-CASSCF(8,8)/def2-SVP//NEVPT2 within the solvent continuum model in CH_2Cl_2, computed independently for the different geometries.

a) S_0 geom. (θ = 18°)

SOCME and E_{ES}		T_1	T_2
		3.02	4.05
S_0	0.0	0	6
S_1	3.95	0	11

b) T_1 geom. (θ = 33°)

SOCME and E_{ES}		T_1	T_2
		2.52	3.96
S_0	0.0	0	12
S_1	3.48	0	21

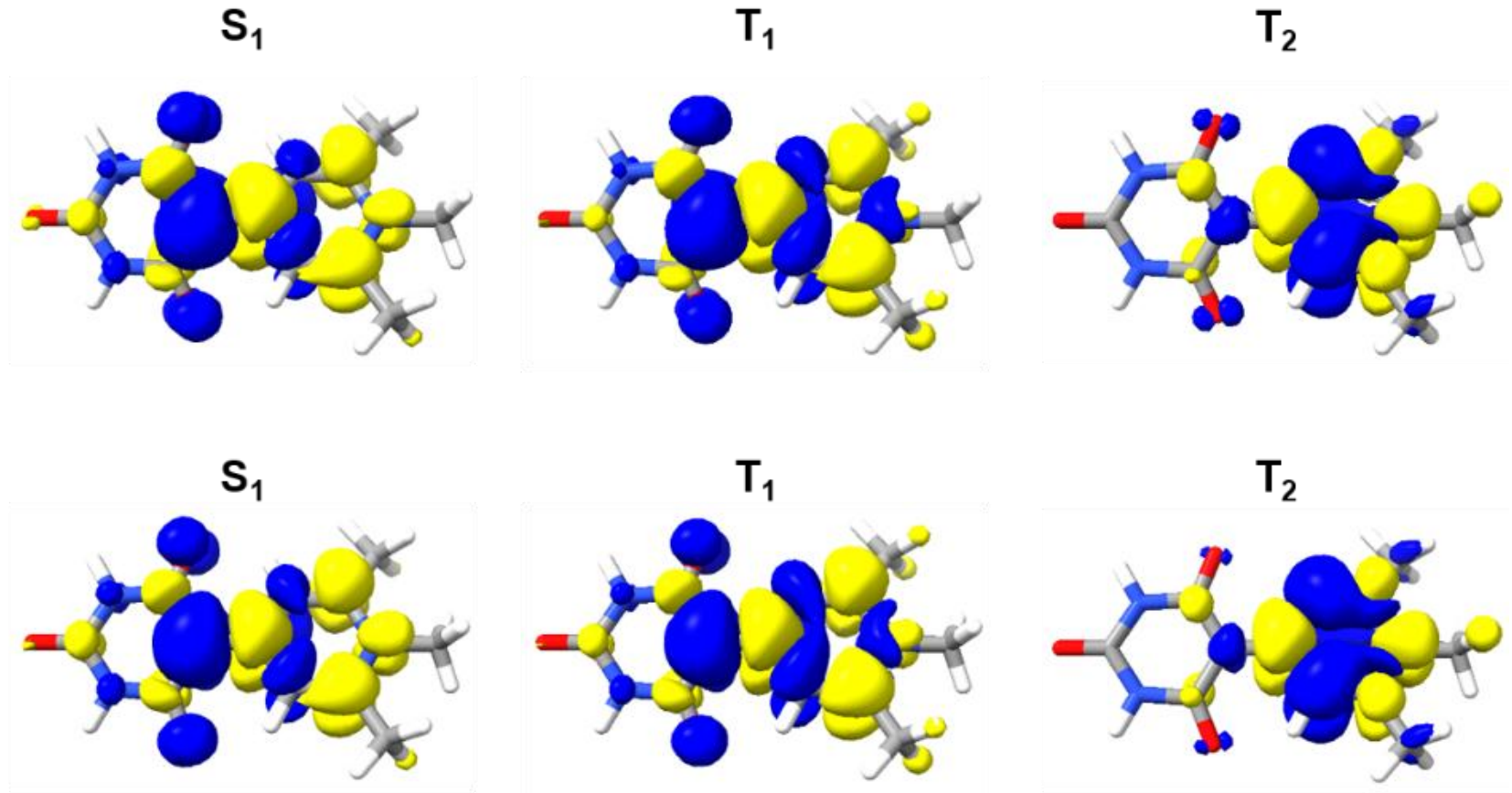

Fig. S29. TD-ωB97X-D3/def2-TZVP//CPCM(CH_2Cl_2) squared reduced SOC matrix elements (SOCME) between triplet and singlet states (in $(cm^{-1})^2$), excited state energies E_{ES} (in eV, relative to S_0) and difference densities of the S_1, T_1 and T_2 states, computed for the (a) S_0 geometry and (b) relaxed T_1 geometry of **PyBA**.

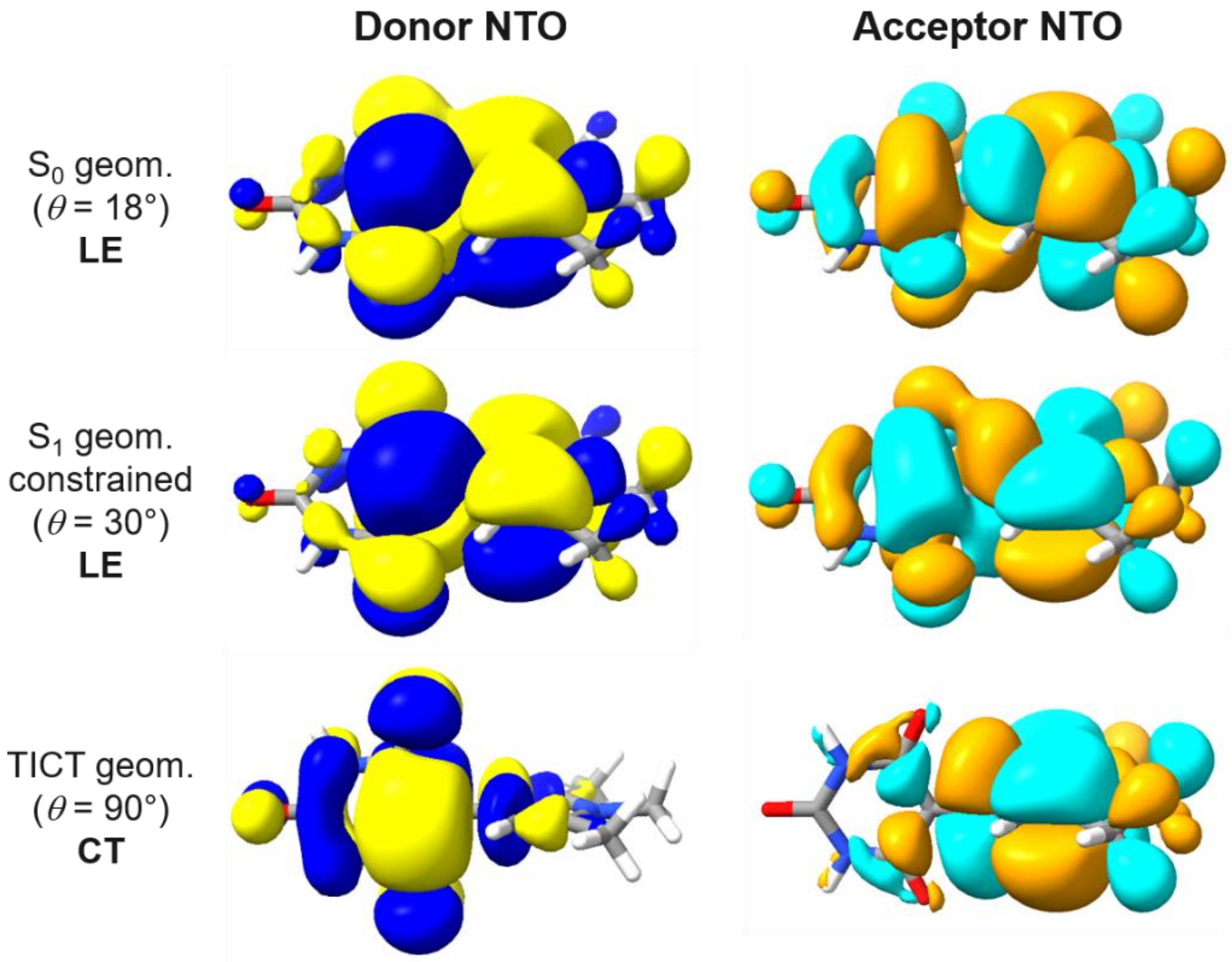

Fig. S30. Natural transition orbital (NTO) analysis of **PyBA** at the CASSCF(8,8)/def2-SVP//CPCM(DCM) level in its relaxed S_0, constrained S_1 and TICT geometries, demonstrating their respective LE or CT character. The structures were optimized at the (TD-)ωB97X-D3/def2-TZVP level of theory within the solvent continuum model in CH_2Cl_2.

8 Supporting References

S1 „Physical Properties of Solvents" datasheet from Sigma-Aldrich, https://www.sigmaaldrich.com/deepweb/assets/sigmaaldrich/marketing/global/documents/614/456/labbasics_pg144.pdf?srsltid=AfmBOorBj7h3KvLgcZ5fU7A71ql0jkSn4n1V8mUpVVD93aw7zZw-VxT8, accessed on April 2026.

S2 Professor Murov's organic solvent table, http://murov.info/orgsolvents.htm, accessed on April 2026.

S3 Sisco research laboratories product datasheet for PEG400, https://www.srlchem.com/product/product-datasheet/productId/79133#:~:text=Specifications,5%20Kg, accessed on April 2026.

S4 K. Arnold, A. Herrmann, L. Pratsch, K. Gawrisch, *Biochim. Biophys. Acta Biomembr.*, 1985, **815**, 515–518, DOI: 10.1016/0005-2736(85)90381-5.

S5 Tokyo Chemical Industry (TCI) product datasheet for PEG400, https://www.tcichemicals.com/IN/en/p/N0443, accessed on April 2026.

S6 BASF product datasheet for PolyTHF, https://www.btc-europe.com/fileadmin/user_upload/Downloads/Brochures-Information/Chemicals/TDSPolyTHF_EU_en.PDF, accessed on April 2026.

S7 F. Cataldo, *Solid State Ion.*, 2001, **139**, 281–291, DOI: 10.1016/S0167-2738(01)00693-2.

S8 Thomas Scientific product datasheet for PolyTHF, https://www.thomassci.com/p/polytetrahydrofuran-1000-stabilised-with-26-di-tert-butyl-4-methylphenol-for-synthesis#:~:text=CAS%20No.:%2025190%2D06,/cm3%20(30%C2%B0C), accessed on April 2026.

S9 J. Karpenko, Y. Niko, V. P. Yakubovskyi, A. O. Gerasov, D. Bonnet, Y. P. Kovtun and A. S. Klymchenko, *J. Mater. Chem. C*, 2016, **4**, 3002–3009, DOI: 10.1039/c5tc03411f.

S10 G. R. Fulmer, A. J. M. Miller, N. H. Sherden, H. E. Gottlieb, A. Nudelman, B. M. Stoltz, J. E. Bercaw and K. I. Goldberg, *Organometallics*, 2010, **29**, 2176–2179, DOI: 10.1021/om100106e.

S11 G. M. Sheldrick, *Acta Crystallog. A: Found. Adv.*, 2015, **71**, 3–8, DOI: 10.1107/s2053273314026370.

S12 G. M. Sheldrick, *Acta Crystallog. A: Found. Adv.*, 2008, **64**, 112–122, DOI: 10.1107/s0108767307043930.

S13 H.-G. Kuball and M. Stolte, in *Comprehensive Chiroptical Spectroscopy: Instrumentation, Methodologies, and Theoretical Simulations*, ed. N. Berova, P. L. Polavarapu, K. Nakanishi and R. W. Woody, John Wiley & Sons, Inc., 2012, Vol. 1, pp. 525-540.

S14 W. Baumann, *Physical Methods of Chemistry*, ed. B. W. Rossiter, J.F. Hamilton, Wiley, New York, 1989, Vol. 3B, p 45.

S15 F. Würthner, S. Yao, J. Schilling, R. Wortmann, M. Redi-Abshiro, E. Mecher, F. Gallego-Gomez and K. Meerholz, *J. Am. Chem. Soc.*, 2001, **123**, 2810–2824, DOI: 10.1021/ja002321g

S16 T.-S. Ahn, R. O. Al-Kaysi, A. M. Müller, K. M. Wentz and C. J. Bardeen, *Rev. Sci. Instrum.*, 2007, **78**, 086105, DOI: 10.1063/1.2768926.

S17 P. Verma, M. Dinesh, P. K. Mitra, A. Santhosh and Y. A. Lakshmanna, *Phys. Chem. Chem. Phys.*, 2025, **27**, 24747–24758, DOI: 10.1039/D5CP03053F

S18 F. Neese, *WIRES Comput. Molec. Sci.*, 2025, **15**, e70019, DOI: 10.1002/wcms.7019.

S19 G. M. J. Barca, C. Bertoni, L. Carrington, D. Datta, N. De Silva, J. E. Deustua, D. G. Fedorov, J. R. Gour, A. O. Gunina, E. Guidez, T. Harville, S. Irle, J. Ivanic, K. Kowalski, S. S. Leang, H. Li, W. Li, J. J. Lutz, I. Magoulas, J. Mato, V. Mironov, H. Nakata, B. Q. Pham, P. Piecuch, D. Poole, S. R. Pruitt, A. P. Rendell, L. B. Roskop, K. Ruedenberg, T. Sattasathuchana, M. W. Schmidt, J. Shen, L. Slipchenko, M. Sosonkina, V. Sundriyal, A. Tiwari, J. L. Galvez Vallejo, B. Westheimer, M. Włoch, P. Xu, F. Zahariev and M. S. Gordon, *J. Chem. Phys.*, 2020, **152**, 154102, DOI: 10.1063/5.0005188.

S20 E. C. Meng, T. D. Goddard, E. F. Pettersen, G. S. Couch, Z. J. Pearson, J. H. Morris and T. E. Ferrin, *Protein Sci.*, 2023, **32**, e4792, DOI: 10.1002/pro.4792.
S21 M. Wang, L. Qian, Y. Guo, H. Wu, M. Liu, W. Gao, G. Li, J. Ding and X. Huang, *Dyes Pigments*, 2019, **160**, 378–385, DOI: 10.1016/j.dyepig.2018.08.018.
S22 Y. Mao, W. Tian, Z. Huang and J. An, *J. Heterocycl. Chem.*, 2014, **51**, 594-597, DOI: 10.1002/jhet.1111.
S23 R. B. K. Siram, K. Tandy, M. Horecha, P. Formanek, M. Stamm, S. Gevorgyan, F. C. Krebs, A. Kiriy, P. Meredith, P. L. Burn, E. B. Namdas and S. Patil, *J. Phys. Chem. C*, 2011, **115**, 14369–14376, DOI: 10.1021/jp204271c.
S24 F. Eiden, *Arch. Pharm.*, 1960, **293**, 404–414, DOI: 10.1002/ardp.19602930407.
S25 C. Kaufmann, D. Bialas, M. Stolte and F. Würthner, *J. Am. Chem. Soc.* 2018, **140**, 9986–9995, DOI: 10.1021/jacs.8b05490.
S26 W. Liptay, *Angew. Chem. Int. Ed.* 1969, **8**, 177–188, DOI: 10.1002/anie.196901771.
S27 L. Onsager, *J. Am. Chem. Soc.* 1936, **58**, 1486–1493, DOI: 10.1021/ja01299a050.
S28 F. Würthner, *Acc. Chem. Res.* 2016, **49**, 868–876, DOI: 10.1021/acs.accounts.6b00042.
